# Supplementary figures and images for: Within-plant genetic drift to control virus adaptation to host resistance genes
Source: PLoS Pathog. 2024 Aug 5;20(8):e1012424. doi: 10.1371/journal.ppat.1012424 (PMC11326801; doi:10.1371/journal.ppat.1012424)

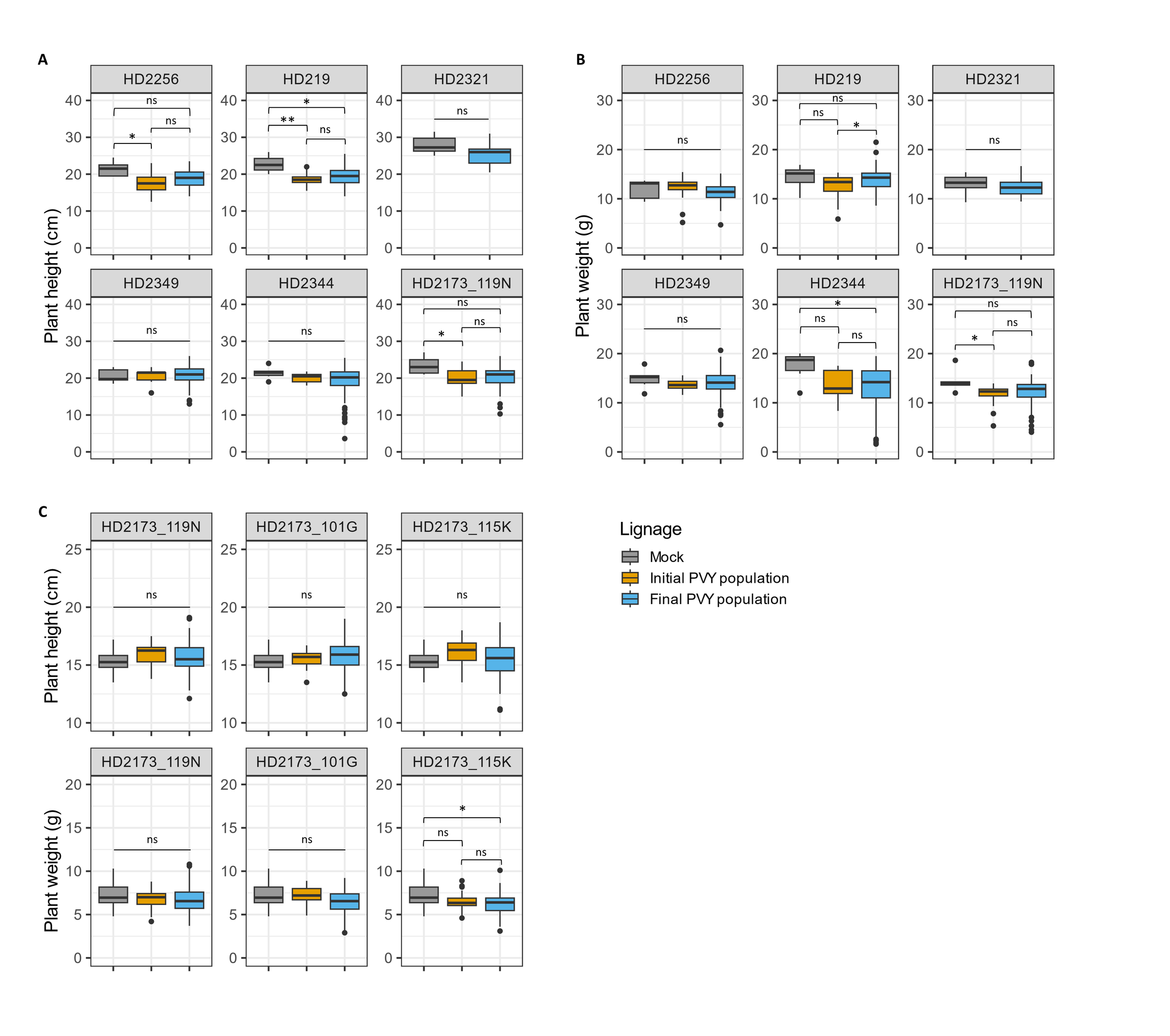

Supplement: S1 Fig — Six plants per DH line were mock-inoculated (gray), while 20 plants per DH line were inoculated either with the variant SON41-119N (orange) or with each of the PVY populations derived from SON41-119N that were serially inoculated on these lines (blue). Boxplots of (A) plant height and (B) fresh weight at 30 days post inoculation are represented. The same experiment (C) was performed independently with the three initial variants SON41-119N, SON41-101G and SON41-115K as well as the resulting populations that were serially inoculated onto HD2173. Significance levels were obtained with the Kruskal–Wallis test followed by the Nemenyi post hoc test (ns: not significant, * p < 0.05, ** p < 0.01). (TIF) [file ppat.1012424.s001.tif]

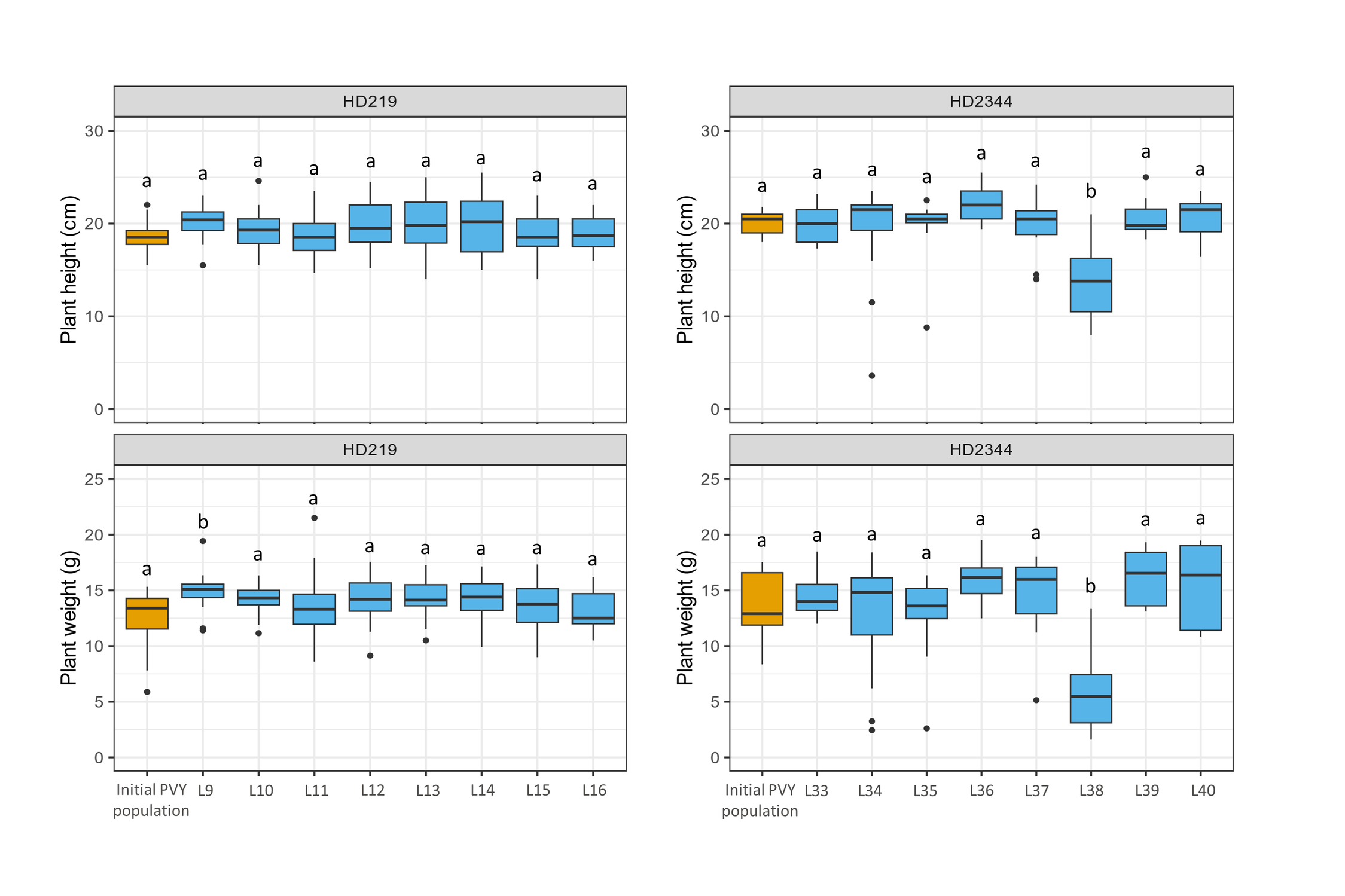

Supplement: S2 Fig — The initial variant SON41-119N (orange) and each of the final PVY populations (blue) were inoculated onto 20 plants. Boxplots of plant height (A) and fresh weight (B) at 30 days post inoculation are represented. The letters a and b indicate the different groups obtained after the comparison of each final PVY population to the initial variant using Dunnett test (p < 0.05). (TIF) [file ppat.1012424.s002.tif]
