# Supplementary material for: Within-plant genetic drift to control virus adaptation to host resistance genes
Source: PLoS Pathog. 2024 Aug 5;20(8):e1012424. doi: 10.1371/journal.ppat.1012424 (PMC11326801; doi:10.1371/journal.ppat.1012424)
Supplement: S1 Table — When possible, the broad-sense heritability (h2) of the trait among plant DH lines was calculated. (DOCX) [file ppat.1012424.s003.docx]

**Table S1.** Traits characterizing each pepper DH line (virus effective population size *N_e_*, corresponding to the effective population size at the onset of systemic infection, estimated from 7 to 10 dpi) or DH line – initial PVY variant combination (virus initial replicative fitness *W*_i_) used for the experimental evolution. When possible, the broad-sense heritability (*h²*) of the trait among plant DH lines was calculated.

| **DH line** | ***N_e_* (*h²*= 0.63)** | ***W*_i_**  **(SON41-119N) (*h²*= 0.85)** | ***W*_i_**  **(SON41-101G)** | ***W*_i_**  **(SON41-115K)** |
| --- | --- | --- | --- | --- |
| **HD2256** | 43 | 0.39 | - | - |
| **HD219** | 42 | 1.08 | - | - |
| **HD2321** | 20 | 0 | - | - |
| **HD2349** | 462 | 0.40 | - | - |
| **HD2344** | 201 | 0.15 | - | - |
| **HD2173** | 459 | 0.79 | 0.33 | 1.33 |
