# Supplementary material for: Within-plant genetic drift to control virus adaptation to host resistance genes
Source: PLoS Pathog. 2024 Aug 5;20(8):e1012424. doi: 10.1371/journal.ppat.1012424 (PMC11326801; doi:10.1371/journal.ppat.1012424)
Supplement: S2 Table — The inoculum was composed of a 2:1 ratio mixture of the two variants in competition, the single mutant SON41-119N being the most concentrated. Thirty days after inoculation, the relative proportions of each variant were estimated by sequencing. Plants were grouped in two categories where either the single (SON41-119N) or double mutant (either SON41-115K-119N or SON41-115M-119N) predominated or was fixed in the population. The plant segregation according to the predominant virus was compared to the frequencies expected in the absence of selection (2:1 ratio of single vs. double PVY mutant) with a chi-squared test and Bonferroni correction for multiple testing (* p < 0.05, *** p < 0.001). (DOCX) [file ppat.1012424.s004.docx]

| **Viruses in competition (inoculum)** | **DH line** | **Number of plants with the double mutant predominant or fixed** | **Number of plants with the single mutant SON41-119N predominant or fixed** | **P-values (chi-squared test) of the experimental frequencies against the expected frequency in the absence of competition** |
| --- | --- | --- | --- | --- |
| SON41-119N *vs.* SON41-115K-119N | HD2173 | 10 | 1 | 1.21x10^-04^ *** |
|  | HD219 | 19 | 1 | 5.00x10^-07^ *** |
|  | HD2256 | 8 | 2 | 3.17x10^-03^ * |
|  | HD2321 | 21 | 0 | 5.00x10^-07^ *** |
|  | HD2344 | 19 | 1 | 5.00x10^-07^ *** |
|  | HD2349 | 19 | 0 | 5.00x10^-07^ *** |
| SON41-119N *vs.* SON41-115M-119N | HD2173 | 17 | 0 | 5.00x10^-07^ *** |
|  | HD219 | 20 | 7 | 1.30x10^-05^ *** |
|  | HD2256 | 7 | 7 | 0.25 |
|  | HD2321 | 17 | 1 | 5.00x10^-07^ *** |
|  | HD2344 | 25 | 1 | 5.00x10^-07^ *** |
|  | HD2349 | 30 | 0 | 5.00x10^-07^ *** |

**Table S2.** Competition experiments between PVY variants in pepper DH lines. The inoculum was composed of a 2:1 ratio mixture of the two variants in competition, the single mutant SON41-119N being the most concentrated. Thirty days after inoculation, the relative proportions of each variant were estimated by sequencing. Plants were grouped in two categories where either the single (SON41-119N) or double mutant (either SON41-115K-119N or SON41-115M-119N) predominated or was fixed in the population. The plant segregation according to the predominant virus was compared to the frequencies expected in the absence of selection (2:1 ratio of single *vs*. double PVY mutant) with a chi-squared test and Bonferroni correction for multiple testing (* p < 0.05, ** p < 0.01, *** p < 0.001).
