# Supplementary material for: Within-plant genetic drift to control virus adaptation to host resistance genes
Source: PLoS Pathog. 2024 Aug 5;20(8):e1012424. doi: 10.1371/journal.ppat.1012424 (PMC11326801; doi:10.1371/journal.ppat.1012424)

**Table S3.** Comparison of virus variant distributions in the plants after one month of competition. Plants belonging to six DH lines were inoculated with a 2:1 ratio mixture of the PVY single mutant SON41-119N and either the double mutant SON41-115K-119N or the double mutant SON41-115M-119N. One month after inoculation, the composition of the virus population in each plant was assessed by sequencing. Plants were grouped in two categories where either (i) SON41-119N or (ii) the double mutant predominated or was fixed in the population. The table displays the non-corrected p-values obtained after performing chi-squared tests to compare the final frequencies of the two variants between the DH lines. The final frequencies were also compared to the null hypothesis (H0), which states that the expected frequencies of the two variants in the absence of competition should correspond to 2/3 of plants where the single mutant SON41-119N is predominant or fixed, and 1/3 of plants where the double mutants SON41-115K-119N or SON41-115M-119N is predominant or fixed. Bonferroni correction was used to correct for multiple testing (* p < 0.05, ** p < 0.01, *** p < 0.001). Significant p-values according to Bonferroni correction are highlighted in grey. Notably, the increase in frequency of mutants SON41-115K-119N or SON41-115M-119N at the expense of mutant SON41-119N in competition experiments was lower in the two DH lines characterized by low *N_e_* and low *s* (HD219 and HD2256; Fig. S1).


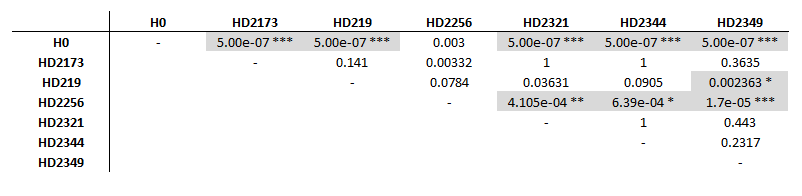

Supplement: S3 Table — Plants belonging to six DH lines were inoculated with a 2:1 ratio mixture of the PVY single mutant SON41-119N and either the double mutant SON41-115K-119N or the double mutant SON41-115M-119N. One month after inoculation, the composition of the virus population in each plant was assessed by sequencing. Plants were grouped in two categories where either (i) SON41-119N or (ii) the double mutant predominated or was fixed in the population. The table displays the non-corrected p-values obtained after performing chi-squared tests to compare the final frequencies of the two variants between the DH lines. The final frequencies were also compared to the null hypothesis (H0), which states that the expected frequencies of the two variants in the absence of competition should correspond to 2/3 of plants where the single mutant SON41-119N is predominant or fixed, and 1/3 of plants where the double mutants SON41-115K-119N or SON41-115M-119N is predominant or fixed. Bonferroni correction was used to correct for multiple testing (* p < 0.05, ** p < 0.01, *** p < 0.001). Significant p-values according to Bonferroni correction are highlighted in grey. (DOCX) [file ppat.1012424.s005.docx]
