# Supplementary material for: Within-plant genetic drift to control virus adaptation to host resistance genes
Source: PLoS Pathog. 2024 Aug 5;20(8):e1012424. doi: 10.1371/journal.ppat.1012424 (PMC11326801; doi:10.1371/journal.ppat.1012424)
Supplement: S2 File — (HTML) [file ppat.1012424.s010.html]

Statistical analyses performed in: Within-plant genetic drift to control virus adaptation to host-resistance genes


Code 

- Show All Code
- Hide All Code

# Statistical analyses performed in: **Within-plant genetic drift to control virus adaptation to host-resistance genes**

#### 2024-05-21

# **1. Packages and datasets loading**

## Packages

```
library(car) # VIF
library(corrplot) # Correlation analyses
library(dplyr) # Dataframe manipulation
library(ggplot2) # Graphics production
library(ggpubr) # qqplot
library(MuMIn) # Model selection
library(PerformanceAnalytics) # Correlation analyses
library(PMCMRplus) # Dunnett and Nemenyi tests
library(pscl) # McFadden's R-squared and R-squared
library(ResourceSelection) # Hosmer-Lemeshow goodness-of-fit test 
library(visreg) # GLM plots
```

## Datasets

All files used must be saved in the same folder as the R Markdown
file.

```
# Fitness
#----------------------------
#----------------------------

# Dataset with Wi and Wf. One value per plant.

Data_Fitness <- read.csv("S1_Dataset.csv", strip.white=T, sep=";")

Data_Fitness$ViralPop <- as.factor(Data_Fitness$ViralPop)
Data_Fitness$Time <- as.factor(Data_Fitness$Time)
Data_Fitness$PlantGeno <- as.factor(Data_Fitness$PlantGeno)
Data_Fitness$PlantGeno2 <- as.factor(Data_Fitness$PlantGeno2)

# Dataset with the mean Wi and Wf measured per viral population.

data_summary <- function(data, varname, groupnames){
  require(plyr)
  
  summary_func <- function(x, col){
    c(mean = mean(x[[col]], na.rm=TRUE),
      se = sd(x[[col]], na.rm=TRUE) / sqrt(length(x[[col]])))
  }
  
  data_sum <- ddply(data, groupnames, .fun=summary_func, col = varname)
  colnames(data_sum)[colnames(data_sum) == "mean"] <- "Fitness"
  
  return(data_sum)
}

Data_Fitness_summary <- data_summary(Data_Fitness, varname = "Fitness", groupnames = c("PlantGeno","Time"))


# Dataset with Wi, Wf and all Ne values. One value per viral population.

Data_GLM <- read.csv("S2_Dataset.csv", strip.white=T, sep=";")

Data_GLM<-cbind(Data_GLM,DeltaFitness=Data_GLM$Wf-Data_GLM$Wi)
GainFit<-rep(0,nrow(Data_GLM)) # Creation of "The probability of replicative fitness gain" variable (GaintFit)
GainFit[which(Data_GLM$DeltaFitness>0)]<-1
Data_GLM<-cbind(Data_GLM,GainFit=GainFit)

# Same dataset without the lignages extincted

DataNoExtinction <- Data_GLM[-which(Data_GLM$Extinction==1),]

# Virulence
#----------------------------
#----------------------------

# Dataset with plant height and fresh weight. One value per plant.

Data_Virulence <- read.csv("S3_Dataset.csv", strip.white=T, sep=";")

Data_Virulence$Height <- as.numeric(Data_Virulence$Height)
Data_Virulence$Weight <- as.numeric(Data_Virulence$Weight)
Data_Virulence$PlantGeno <- as.factor(Data_Virulence$PlantGeno)
Data_Virulence$Time <- as.factor(Data_Virulence$Time)
Data_Virulence$ViralPop <- as.factor(Data_Virulence$ViralPop)

# Separation of datasets by experiment

Data_Virulence_A <- filter(Data_Virulence, Exp=="A")
Data_Virulence_B <- filter(Data_Virulence, Exp=="B")
```

# **2. Figures, tables and statistics**

All the figures and tables generated in R and the associated
statistics are shown below.

## **Figure 1:** Features of the pepper DH line - initial PVY variant combinations used for the evolution experiment

The two DH line - initial PVY variant combinations with both low Wi
and Ne levels are highlighted in blue.

```
HD2173_115K <- c(paste0("HD2173"), paste0("SON41-115K"))
HD2173_119N <- c(paste0("HD2173"), paste0("SON41-119N"))
HD2173_101G <- c(paste0("HD2173"), paste0("SON41-101G"))

ggplot(Data_GLM, aes(x=Ne_7_10, y=Wi, color= factor(Ne_7_10))) +
  geom_point(size=6) + 
  xlab(expression(italic(N[e]))) +
   ylab(bquote(italic(W)[i])) +
  scale_color_manual(values=c("#93CDDD", "black", "#93CDDD", "black", "black", "black", "black", "black"))+
  annotate("text", x=45, y=1.18, label= "HD219", size=4) +
  annotate("text", x=45, y=0.50, label= "HD2256", size=4) +
  annotate("text", x=33, y=0.10, label= "HD2321", size=4) +
  annotate("text", x=200, y=0.25, label= "HD2344", size=4) +
  annotate("text", x=450, y=0.50, label= "HD2349", size=4) +
  annotate("text", x=410, y=c(1.33, 1.25), label=HD2173_115K, size=4) +
  annotate("text", x=410, y=c(0.78, 0.70), label=HD2173_119N, size=4) +
  annotate("text", x=410, y=c(0.32, 0.24), label=HD2173_101G, size=4) +
  theme_bw(base_size = 16) +
  theme(legend.position="none")
```

```
# ggsave("./Figure1.svg")
```

## **Figure 4:** Replicative fitness changes during the evolution experiment

The sub-sections of Figure 4 and the associated statistical analyses
are presented bellow. The figures and statistical analyses of the DH
lines not presented in the article are also shown below.

### **Figure 4A: All pepper lines**

#### Figure 4A

**Replicative fitness** of the initial PVY variant (Wi)
(**black**) and of the
final populations (Wf) (**grey**) for the 8 pepper DH
line - initial PVY variant combinations:

```
Data_Fitness_summary$PlantGeno = factor(Data_Fitness_summary$PlantGeno, 
    levels=c("HD2256","HD219","HD2321","HD2349","HD2344","HD2173N","HD2173G","HD2173K"))

Data_Fitness_summary$Time = factor(Data_Fitness_summary$Time, 
    levels=c("Initial","Final"))

ggplot(Data_Fitness_summary, aes(x=PlantGeno, y=Fitness, fill=Time))+
geom_bar(stat="identity", position=position_dodge(), color="black", width = 0.7)+
  scale_fill_manual(values=c("black","grey")) +
  geom_errorbar(aes(ymin = Fitness, ymax=Fitness+se), width=.2,position=position_dodge(.7)) +
  ylab("PVY replicative fitness") +
  xlab("") +
  theme_bw(base_size = 13)
```

```
# ggsave("./Figure4A.svg")
```

#### Statistics

**Wilcoxon tests** comparing the mean replicative
fitness of the initial and final PVY populations in each DH line:

**HD2256**

```
Data_Fitness_HD2256_Wi <- filter(Data_Fitness, PlantGeno2=="HD2256_Initial")
Data_Fitness_HD2256_Wf <- filter(Data_Fitness, PlantGeno2=="HD2256_Final")
wilcox.test(Data_Fitness_HD2256_Wi$Fitness, Data_Fitness_HD2256_Wf$Fitness)
```

```
## 
##  Wilcoxon rank sum test with continuity correction
## 
## data:  Data_Fitness_HD2256_Wi$Fitness and Data_Fitness_HD2256_Wf$Fitness
## W = 894, p-value = 0.1562
## alternative hypothesis: true location shift is not equal to 0
```

**HD219**

```
Data_Fitness_HD219_Wi <- filter(Data_Fitness, PlantGeno2=="HD219_Initial")
Data_Fitness_HD219_Wf <- filter(Data_Fitness, PlantGeno2=="HD219_Final")
wilcox.test(Data_Fitness_HD219_Wi$Fitness, Data_Fitness_HD219_Wf$Fitness)
```

```
## 
##  Wilcoxon rank sum test with continuity correction
## 
## data:  Data_Fitness_HD219_Wi$Fitness and Data_Fitness_HD219_Wf$Fitness
## W = 1725, p-value = 0.09618
## alternative hypothesis: true location shift is not equal to 0
```

**HD2321**

```
Data_Fitness_HD2321_Wi <- filter(Data_Fitness, PlantGeno2=="HD2321_Initial")
Data_Fitness_HD2321_Wf <- filter(Data_Fitness, PlantGeno2=="HD2321_Final")
wilcox.test(Data_Fitness_HD2321_Wi$Fitness, Data_Fitness_HD2321_Wf$Fitness)
```

```
## 
##  Wilcoxon rank sum test with continuity correction
## 
## data:  Data_Fitness_HD2321_Wi$Fitness and Data_Fitness_HD2321_Wf$Fitness
## W = 0, p-value = 1.175e-08
## alternative hypothesis: true location shift is not equal to 0
```

**HD2349**

```
Data_Fitness_HD2349_Wi <- filter(Data_Fitness, PlantGeno2=="HD2349_Initial")
Data_Fitness_HD2349_Wf <- filter(Data_Fitness, PlantGeno2=="HD2349_Final")
wilcox.test(Data_Fitness_HD2349_Wi$Fitness, Data_Fitness_HD2349_Wf$Fitness)
```

```
## 
##  Wilcoxon rank sum test with continuity correction
## 
## data:  Data_Fitness_HD2349_Wi$Fitness and Data_Fitness_HD2349_Wf$Fitness
## W = 502, p-value = 2.897e-05
## alternative hypothesis: true location shift is not equal to 0
```

**HD2344**

```
Data_Fitness_HD2344_Wi <- filter(Data_Fitness, PlantGeno2=="HD2344_Initial")
Data_Fitness_HD2344_Wf <- filter(Data_Fitness, PlantGeno2=="HD2344_Final")
wilcox.test(Data_Fitness_HD2344_Wi$Fitness, Data_Fitness_HD2344_Wf$Fitness)
```

```
## 
##  Wilcoxon rank sum test with continuity correction
## 
## data:  Data_Fitness_HD2344_Wi$Fitness and Data_Fitness_HD2344_Wf$Fitness
## W = 556.5, p-value = 0.0001644
## alternative hypothesis: true location shift is not equal to 0
```

**HD2173\_119N**

```
Data_Fitness_HD2173N_Wi <- filter(Data_Fitness, PlantGeno2=="HD2173N_Initial")
Data_Fitness_HD2173N_Wf <- filter(Data_Fitness, PlantGeno2=="HD2173N_Final")
wilcox.test(Data_Fitness_HD2173N_Wi$Fitness, Data_Fitness_HD2173N_Wf$Fitness)
```

```
## 
##  Wilcoxon rank sum test with continuity correction
## 
## data:  Data_Fitness_HD2173N_Wi$Fitness and Data_Fitness_HD2173N_Wf$Fitness
## W = 1451, p-value = 0.4528
## alternative hypothesis: true location shift is not equal to 0
```

**HD2173\_101G**

```
Data_Fitness_HD2173G_Wi <- filter(Data_Fitness, PlantGeno2=="HD2173G_Initial")
Data_Fitness_HD2173G_Wf <- filter(Data_Fitness, PlantGeno2=="HD2173G_Final")
wilcox.test(Data_Fitness_HD2173G_Wi$Fitness, Data_Fitness_HD2173G_Wf$Fitness)
```

```
## 
##  Wilcoxon rank sum test with continuity correction
## 
## data:  Data_Fitness_HD2173G_Wi$Fitness and Data_Fitness_HD2173G_Wf$Fitness
## W = 498, p-value = 6.314e-07
## alternative hypothesis: true location shift is not equal to 0
```

**HD2173\_115K**

```
Data_Fitness_HD2173K_Wi <- filter(Data_Fitness, PlantGeno2=="HD2173K_Initial")
Data_Fitness_HD2173K_Wf <- filter(Data_Fitness, PlantGeno2=="HD2173K_Final")
wilcox.test(Data_Fitness_HD2173K_Wi$Fitness, Data_Fitness_HD2173K_Wf$Fitness)
```

```
## 
##  Wilcoxon rank sum test with continuity correction
## 
## data:  Data_Fitness_HD2173K_Wi$Fitness and Data_Fitness_HD2173K_Wf$Fitness
## W = 1692, p-value = 0.6771
## alternative hypothesis: true location shift is not equal to 0
```

### **Figure 4B: HD2321**

#### Figure 4B

**Replicative fitness** of the initial PVY variants
(**black**) and the
eight derived final populations (**grey**) in HD2321:

```
Data_Fitness_HD2321 <- filter(Data_Fitness, PlantGeno=="HD2321")
Data_Fitness_summary_HD2321 <- data_summary(Data_Fitness_HD2321, varname = "Fitness", groupnames = c("ViralPop"))

Data_Fitness_summary_HD2321$ViralPop = factor(Data_Fitness_summary_HD2321$ViralPop, 
    levels=c("Initial","L23","L24","L22","L17","L18","L19","L20","L21"))

ggplot(Data_Fitness_summary_HD2321, aes(x=ViralPop, y=Fitness, fill=ViralPop))+
geom_bar(stat="identity", position=position_dodge(), color="black")+
  scale_fill_manual(values=c("black", rep("grey",8))) +
  geom_errorbar(aes(ymin = Fitness, ymax=Fitness+se), width=.2,position=position_dodge(.9)) +
  ylab("PVY replicative fitness") +
  xlab("") +
  ylim(0,1) +
  theme_bw(base_size = 15) + 
  theme(legend.position="none")
```

```
# ggsave("./Figure4B.svg")
```

#### Statistics

**Dunnet test** comparing the mean replicative fitness
of the final viral populations against the mean replicative fitness of
the initial viral population in HD2321:

```
dunnettTest(x=Data_Fitness_HD2321$Fitness, g=Data_Fitness_HD2321$ViralPop, alternative="greater")
```

```
##     Initial
## L23 0.0025 
## L24 0.0198
```

### **Figure 4C: HD2349**

#### Figure 4C

Replicative fitness of the initial PVY variants (**black**) and the eight
derived final populations (**grey**) in HD2349:

```
Data_Fitness_HD2349 <- filter(Data_Fitness, PlantGeno=="HD2349")
Data_Fitness_summary_HD2349 <- data_summary(Data_Fitness_HD2349, varname = "Fitness", groupnames = c("ViralPop"))

Data_Fitness_summary_HD2349$ViralPop = factor(Data_Fitness_summary_HD2349$ViralPop, 
    levels=c("Initial","L26","L28","L27","L25","L29","L30","L31","L32"))

ggplot(Data_Fitness_summary_HD2349, aes(x=ViralPop, y=Fitness, fill=ViralPop))+
geom_bar(stat="identity", position=position_dodge(), color="black")+
  scale_fill_manual(values=c("black", rep("grey",8))) +
  geom_errorbar(aes(ymin = Fitness, ymax=Fitness+se), width=.2,position=position_dodge(.9)) +
  ylab("PVY replicative fitness") +
  xlab("") +
  theme_bw(base_size = 13) + 
  theme(legend.position="none")
```

```
# ggsave("./Figure4C.svg")
```

#### Statistics

**Dunnet test** comparing the mean replicative fitness
of the final viral populations against the mean replicative fitness of
the initial viral population in HD2349:

```
dunnettTest(x=Data_Fitness_HD2349$Fitness, g=Data_Fitness_HD2349$ViralPop, alternative="greater")
```

```
##     Initial
## L25 0.063  
## L26 0.015  
## L27 0.046  
## L28 0.021  
## L29 0.140  
## L30 0.475  
## L31 0.718  
## L32 0.908
```

### **Figure 4D: HD2344**

#### Figure 4D

Replicative fitness of the initial PVY variants (**black**) and the eight
derived final populations (**grey**) in HD2344:

```
Data_Fitness_HD2344 <- filter(Data_Fitness, PlantGeno=="HD2344")
Data_Fitness_summary_HD2344 <- data_summary(Data_Fitness_HD2344, varname = "Fitness", groupnames = c("ViralPop"))

Data_Fitness_summary_HD2344$ViralPop = factor(Data_Fitness_summary_HD2344$ViralPop, 
    levels=c("Initial","L38","L34","L35","L36","L33","L37","L39","L40"))

ggplot(Data_Fitness_summary_HD2344, aes(x=ViralPop, y=Fitness, fill=ViralPop))+
geom_bar(stat="identity", position=position_dodge(), color="black")+
  scale_fill_manual(values=c("black", rep("grey",8))) +
  geom_errorbar(aes(ymin = Fitness, ymax=Fitness+se), width=.2,position=position_dodge(.9)) +
  ylab("PVY replicative fitness") +
  xlab("") +
  theme_bw(base_size = 13) + 
  theme(legend.position="none")
```

```
# ggsave("./Figure4D.svg")
```

#### Statistics

**Dunnet test** comparing the mean replicative fitness
of the final viral populations against the mean replicative fitness of
the initial viral population in HD2344:

```
dunnettTest(x=Data_Fitness_HD2344$Fitness, g=Data_Fitness_HD2344$ViralPop, alternative="greater")
```

```
##     Initial
## L33 0.2933 
## L34 0.0024 
## L35 0.0498 
## L36 0.1222 
## L37 0.5357 
## L38 0.0028 
## L39 0.9248 
## L40 0.9646
```

### **Figure 4E: HD2173\_101G**

#### Figure 4E

**Replicative fitness** of the initial PVY variants
(**black**) and the
eight derived final populations (**grey**) in HD2173 - 101G:

```
Data_Fitness_HD2173G <- filter(Data_Fitness, PlantGeno=="HD2173G")
Data_Fitness_summary_HD2173G <- data_summary(Data_Fitness_HD2173G, varname = "Fitness", groupnames = c("ViralPop"))

Data_Fitness_summary_HD2173G$ViralPop = factor(Data_Fitness_summary_HD2173G$ViralPop, 
    levels=c("Initial","L52","L54","L51","L53","L56","L55","L49","L50"))

ggplot(Data_Fitness_summary_HD2173G, aes(x=ViralPop, y=Fitness, fill=ViralPop))+
geom_bar(stat="identity", position=position_dodge(), color="black")+
  scale_fill_manual(values=c("black", rep("grey",8))) +
  geom_errorbar(aes(ymin = Fitness, ymax=Fitness+se), width=.2,position=position_dodge(.9)) +
  ylab("PVY replicative fitness") +
  xlab("") +
  theme_bw(base_size = 13) + 
  theme(legend.position="none")
```

```
# ggsave("./Figure4E.svg")
```

#### Statistics

**Dunnet test** comparing the mean replicative fitness
of the final viral populations against the mean replicative fitness of
the initial viral population in HD2173 - 101G:

```
dunnettTest(x=Data_Fitness_HD2173G$Fitness, g=Data_Fitness_HD2173G$ViralPop, alternative="greater")
```

```
##     Initial
## L49 0.01250
## L50 0.02169
## L51 0.00031
## L52 1.1e-07
## L53 0.00262
## L54 0.00012
## L55 0.00974
## L56 0.00244
```

### **Additional figure: HD2173\_119N**

#### Additional figure

**Replicative fitness** of the initial PVY variants
(**black**) and the
eight derived final populations (**grey**) in HD2173 - 119N:

```
Data_Fitness_HD2173N <- filter(Data_Fitness, PlantGeno=="HD2173N")
Data_Fitness_summary_HD2173N <- data_summary(Data_Fitness_HD2173N, varname = "Fitness", groupnames = c("ViralPop"))

Data_Fitness_summary_HD2173N$ViralPop = factor(Data_Fitness_summary_HD2173N$ViralPop, 
    levels=c("Initial","L47","L41","L43","L45","L42","L48","L44","L46"))

ggplot(Data_Fitness_summary_HD2173N, aes(x=ViralPop, y=Fitness, fill=ViralPop))+
geom_bar(stat="identity", position=position_dodge(), color="black")+
  scale_fill_manual(values=c("black", rep("grey",8))) +
  geom_errorbar(aes(ymin = Fitness, ymax=Fitness+se), width=.2,position=position_dodge(.9)) +
  ylab("PVY replicative fitness") +
  xlab("") +
  theme_bw(base_size = 13) + 
  theme(legend.position="none")
```

#### Statistics

**Dunnet test** comparing the mean replicative fitness
of the final viral populations against the mean replicative fitness of
the initial viral population in HD2173 - 119N:

```
dunnettTest(x=Data_Fitness_HD2173N$Fitness, g=Data_Fitness_HD2173N$ViralPop, alternative="less")
```

```
##     Initial
## L41 0.98   
## L42 0.74   
## L43 0.86   
## L44 0.35   
## L45 0.83   
## L46 0.20   
## L47 0.99   
## L48 0.46
```

### **Additional figure: HD2173\_115K**

#### Additional figure

**Replicative fitness** of the initial PVY variants
(**black**) and the
eight derived final populations (**grey**) in HD2173 - 115K:

```
Data_Fitness_HD2173K <- filter(Data_Fitness, PlantGeno=="HD2173K")
Data_Fitness_summary_HD2173K <- data_summary(Data_Fitness_HD2173K, varname = "Fitness", groupnames = c("ViralPop"))

Data_Fitness_summary_HD2173K$ViralPop = factor(Data_Fitness_summary_HD2173K$ViralPop, 
    levels=c("Initial","L64","L63","L60","L62","L57","L58","L59","L61"))

ggplot(Data_Fitness_summary_HD2173K, aes(x=ViralPop, y=Fitness, fill=ViralPop))+
geom_bar(stat="identity", position=position_dodge(), color="black")+
  scale_fill_manual(values=c("black", rep("grey",8))) +
  geom_errorbar(aes(ymin = Fitness, ymax=Fitness+se), width=.2,position=position_dodge(.9)) +
  ylab("PVY replicative fitness") +
  xlab("") +
  theme_bw(base_size = 13) + 
  theme(legend.position="none")
```

#### Statistics

**Dunnet test** comparing the mean replicative fitness
of the final viral populations against the mean replicative fitness of
the initial viral population in HD2173 - 115K:

```
dunnettTest(x=Data_Fitness_HD2173K$Fitness, g=Data_Fitness_HD2173K$ViralPop)
```

```
##     Initial
## L57 1.00   
## L58 1.00   
## L59 1.00   
## L60 1.00   
## L61 0.71   
## L62 1.00   
## L63 1.00   
## L64 1.00
```

### **Additional figure: HD219**

#### Additional figure

**Replicative fitness** of the initial PVY variants
(**black**) and the
eight derived final populations (**grey**) in HD219:

```
Data_Fitness_HD219 <- filter(Data_Fitness, PlantGeno=="HD219")
Data_Fitness_summary_HD219 <- data_summary(Data_Fitness_HD219, varname = "Fitness", groupnames = c("ViralPop"))

Data_Fitness_summary_HD219$ViralPop = factor(Data_Fitness_summary_HD219$ViralPop, 
    levels=c("Initial","L16","L12","L15","L9","L11","L10","L13","L14"))

ggplot(Data_Fitness_summary_HD219, aes(x=ViralPop, y=Fitness, fill=ViralPop))+
geom_bar(stat="identity", position=position_dodge(), color="black")+
  scale_fill_manual(values=c("black", rep("grey",8))) +
  geom_errorbar(aes(ymin = Fitness, ymax=Fitness+se), width=.2,position=position_dodge(.9)) +
  ylab("PVY replicative fitness") +
  xlab("") +
  theme_bw(base_size = 13) + 
  theme(legend.position="none")
```

#### Statistics

**Dunnet test** comparing the mean replicative fitness
of the final viral populations against the mean replicative fitness of
the initial viral population in HD219:

```
dunnettTest(x=Data_Fitness_HD219$Fitness, g=Data_Fitness_HD219$ViralPop)
```

```
##     Initial
## L10 1.0    
## L11 1.0    
## L12 1.0    
## L13 1.0    
## L14 1.0    
## L15 1.0    
## L16 0.4    
## L9  1.0
```

### **Additional figure: HD2256**

#### Additional figure

Replicative fitness of the initial PVY variants (**black**) and the eight
derived final populations (**grey**) in HD2256:

```
Data_Fitness_HD2256 <- filter(Data_Fitness, PlantGeno=="HD2256")
Data_Fitness_summary_HD2256 <- data_summary(Data_Fitness_HD2256, varname = "Fitness", groupnames = c("ViralPop"))

Data_Fitness_summary_HD2256$ViralPop = factor(Data_Fitness_summary_HD2256$ViralPop, 
    levels=c("Initial","L6","L4","L7","L5","L8","L1","L2","L3"))

ggplot(Data_Fitness_summary_HD2256, aes(x=ViralPop, y=Fitness, fill=ViralPop))+
geom_bar(stat="identity", position=position_dodge(), color="black")+
  scale_fill_manual(values=c("black", rep("grey",8))) +
  geom_errorbar(aes(ymin = Fitness, ymax=Fitness+se), width=.2,position=position_dodge(.9)) +
  ylab("PVY replicative fitness") +
  xlab("") +
  theme_bw(base_size = 13) + 
  theme(legend.position="none")
```

#### Statistics

**Dunnet test** comparing the mean replicative fitness
of the final viral populations against the mean replicative fitness of
the initial viral population in HD2256:

```
dunnettTest(x=Data_Fitness_HD2256$Fitness, g=Data_Fitness_HD2256$ViralPop)
```

```
##    Initial
## L4 1.00   
## L5 1.00   
## L6 1.00   
## L7 1.00   
## L8 0.42
```

## **Figure 5:** Responses of the probability of replicative fitness gain (A) and final replicative fitness (Wf) (B) to the initial virus replicative fitness (Wi) and the bottleneck size at the onset of systemic infection (Ne)

### **1. Statistical analysis**

#### **1.1 Effect of pepper genotype on PVY evolution**

```
# Effect of Pepper genotype on the number of fixed VPg mutations 
#----------------------------------------------------------------------

Nb_mutations <- c(8,0,1,1,0,4,7,6)
expected <- c(rep(1/8,8))

chisq.test(Nb_mutations, p=expected)
```

```
## 
##  Chi-squared test for given probabilities
## 
## data:  Nb_mutations
## X-squared = 22.481, df = 7, p-value = 0.002097
```

```
# Effect of Pepper genotype on change in PVY replicative fitness
#----------------------------------------------------------------------

summary(aov(Wf ~ PlantGeno2, data=DataNoExtinction))
```

```
##             Df Sum Sq Mean Sq F value   Pr(>F)    
## PlantGeno2   7  7.385  1.0550   13.68 1.71e-09 ***
## Residuals   47  3.625  0.0771                     
## ---
## Signif. codes:  0 '***' 0.001 '**' 0.01 '*' 0.05 '.' 0.1 ' ' 1
```

#### **1.2 Analysis of explanatory variables**

We have 7 potential explanatory variables:

- Ne\_inoc: Ne estimated at the inoculation step in each pepper
  line.
- Ne\_1\_6: Ne estimated from 1 to 6 dpi in each pepper line.
- Ne\_7\_10: Ne estimated from 7 to 10 dpi in each pepper line.
- Ne\_11\_14: Ne estimated from 11 to 14 dpi in each pepper line.
- Ne\_15\_34: Ne estimated from 15 to 34 dpi in each pepper line.
- Ne\_harmo: the harmonic mean of Ne at each dpi in each pepper
  line.
- Wi: the initial replicative fitness of PVY in each pepper line.

Ne\_harmo, Ne\_7\_10, Ne\_11\_14, Ne\_15\_34 and Ne\_inoc are strongly
positively correlated (Spearman’s coefficient ranging between 0.83 and
0.39, pvalues < 0.01). Ne\_1\_6 and Wi are strongly negatively
correlated (Spearman’s coefficient = -0.55, pvalue < 0.001).

##### Correlation plot 1

```
Mcor <- cor(Data_GLM[,c("Wi","Ne_harmo", "Ne_1_6","Ne_7_10","Ne_11_14","Ne_15_34", "Ne_inoc")], method= "spearman")
corrplot.mixed(Mcor, lower.col = "black", number.cex = .7, tl.cex=0.65)
```

##### Correlation plot 2

```
chart.Correlation(Data_GLM [,c("Wi","Ne_harmo", "Ne_1_6","Ne_7_10","Ne_11_14","Ne_15_34", "Ne_inoc")], histogram=F, method= "spearman")
```

#### **1.3 GLM**

Two response variables were tested: (i) the probability of PVY
replicative fitness gain during the evolution experiment, which
distinguishes the PVY lineages showing replicative fitness gain (n=33
out of 64 lineages) or replicative fitness loss (n=31 out of 64,
including lineages that get extinct) after 7 infection cycles and (ii)
the final replicative fitness Wf of the 55 lineages that did not go to
extinction during the experiment.

##### *1.3.1 Probability of PVY replicative fitness gain (GaintFit)*

We assumed a binomial distribution for the probability of replicative
fitness gain.

###### VIF 3 variables

**VIF:** If three explanatory variables are retained in
the model, the Variance Inflation Factors (Vif) values are extremely
high, because as we have seen, several explanatory variables are highly
correlated.

```
# Models with 3 explanatory variables
#-----------------------------------------------------

## Wi and two Ne estimates

Mod_GainFit_1 <- glm(GainFit ~ Wi*Ne_1_6 + Wi*Ne_7_10 + Ne_1_6*Ne_7_10, data=Data_GLM, family=binomial)
Mod_GainFit_2 <- glm(GainFit ~ Wi*Ne_1_6 + Wi*Ne_11_14 + Ne_1_6*Ne_11_14, data=Data_GLM, family=binomial)
Mod_GainFit_3 <- glm(GainFit ~ Wi*Ne_1_6 + Wi*Ne_15_34 + Ne_1_6*Ne_15_34, data=Data_GLM, family=binomial)
Mod_GainFit_4 <- glm(GainFit ~ Wi*Ne_1_6 + Wi*Ne_harmo + Ne_1_6*Ne_harmo, data=Data_GLM, family=binomial)
Mod_GainFit_5 <- glm(GainFit ~ Wi*Ne_1_6 + Wi*Ne_inoc + Ne_1_6*Ne_inoc, data=Data_GLM, family=binomial)
Mod_GainFit_6 <- glm(GainFit ~ Wi*Ne_7_10 + Wi*Ne_11_14 + Ne_7_10*Ne_11_14, data=Data_GLM, family=binomial)
Mod_GainFit_7 <- glm(GainFit ~ Wi*Ne_7_10 + Wi*Ne_15_34 + Ne_7_10*Ne_15_34, data=Data_GLM, family=binomial)
Mod_GainFit_8 <- glm(GainFit ~ Wi*Ne_7_10 + Wi*Ne_harmo + Ne_7_10*Ne_harmo, data=Data_GLM, family=binomial)
Mod_GainFit_9 <- glm(GainFit ~ Wi*Ne_7_10 + Wi*Ne_inoc + Ne_7_10*Ne_inoc, data=Data_GLM, family=binomial)
Mod_GainFit_10 <- glm(GainFit ~ Wi*Ne_11_14 + Wi*Ne_15_34 + Ne_11_14*Ne_15_34, data=Data_GLM, family=binomial)
Mod_GainFit_11 <- glm(GainFit ~ Wi*Ne_11_14 + Wi*Ne_harmo + Ne_11_14*Ne_harmo, data=Data_GLM, family=binomial)
Mod_GainFit_12 <- glm(GainFit ~ Wi*Ne_11_14 + Wi*Ne_inoc + Ne_11_14*Ne_inoc, data=Data_GLM, family=binomial)
Mod_GainFit_13 <- glm(GainFit ~ Wi*Ne_15_34 + Wi*Ne_harmo + Ne_15_34*Ne_harmo, data=Data_GLM, family=binomial)
Mod_GainFit_14 <- glm(GainFit ~ Wi*Ne_15_34 + Wi*Ne_inoc + Ne_15_34*Ne_inoc, data=Data_GLM, family=binomial)
Mod_GainFit_15 <- glm(GainFit ~ Wi*Ne_harmo + Wi*Ne_inoc + Ne_harmo*Ne_inoc, data=Data_GLM, family=binomial)

## Three Ne estimates

Mod_GainFit_16 <- glm(GainFit ~ Ne_1_6*Ne_7_10 + Ne_1_6*Ne_11_14 + Ne_7_10*Ne_11_14, data=Data_GLM, family=binomial)
Mod_GainFit_17 <- glm(GainFit ~ Ne_1_6*Ne_7_10 + Ne_1_6*Ne_15_34 + Ne_7_10*Ne_15_34, data=Data_GLM, family=binomial)
Mod_GainFit_18 <- glm(GainFit ~ Ne_1_6*Ne_7_10 + Ne_1_6*Ne_harmo + Ne_7_10*Ne_harmo, data=Data_GLM, family=binomial)
Mod_GainFit_19 <- glm(GainFit ~ Ne_1_6*Ne_7_10 + Ne_1_6*Ne_inoc + Ne_7_10*Ne_inoc, data=Data_GLM, family=binomial)
Mod_GainFit_20 <- glm(GainFit ~ Ne_1_6*Ne_11_14 + Ne_1_6*Ne_15_34 + Ne_11_14*Ne_15_34, data=Data_GLM, family=binomial)
Mod_GainFit_21 <- glm(GainFit ~ Ne_1_6*Ne_11_14 + Ne_1_6*Ne_harmo + Ne_11_14*Ne_harmo, data=Data_GLM, family=binomial)
Mod_GainFit_22 <- glm(GainFit ~ Ne_1_6*Ne_11_14 + Ne_1_6*Ne_inoc + Ne_11_14*Ne_inoc, data=Data_GLM, family=binomial)
Mod_GainFit_23 <- glm(GainFit ~ Ne_1_6*Ne_15_34 + Ne_1_6*Ne_harmo + Ne_15_34*Ne_harmo, data=Data_GLM, family=binomial)
Mod_GainFit_24 <- glm(GainFit ~ Ne_1_6*Ne_15_34 + Ne_1_6*Ne_inoc + Ne_15_34*Ne_inoc, data=Data_GLM, family=binomial)
Mod_GainFit_25 <- glm(GainFit ~ Ne_1_6*Ne_harmo + Ne_1_6*Ne_inoc + Ne_harmo*Ne_inoc, data=Data_GLM, family=binomial)
Mod_GainFit_26 <- glm(GainFit ~ Ne_7_10*Ne_11_14 + Ne_7_10*Ne_15_34 + Ne_11_14*Ne_15_34, data=Data_GLM, family=binomial)
Mod_GainFit_27 <- glm(GainFit ~ Ne_7_10*Ne_11_14 + Ne_7_10*Ne_harmo + Ne_11_14*Ne_harmo, data=Data_GLM, family=binomial)
Mod_GainFit_28 <- glm(GainFit ~ Ne_7_10*Ne_11_14 + Ne_7_10*Ne_inoc + Ne_11_14*Ne_inoc, data=Data_GLM, family=binomial)
Mod_GainFit_29 <- glm(GainFit ~ Ne_7_10*Ne_15_34 + Ne_7_10*Ne_harmo + Ne_15_34*Ne_harmo, data=Data_GLM, family=binomial)
Mod_GainFit_30 <- glm(GainFit ~ Ne_7_10*Ne_15_34 + Ne_7_10*Ne_inoc + Ne_15_34*Ne_inoc, data=Data_GLM, family=binomial)
Mod_GainFit_31 <- glm(GainFit ~ Ne_7_10*Ne_harmo + Ne_7_10*Ne_inoc + Ne_harmo*Ne_inoc, data=Data_GLM, family=binomial)
Mod_GainFit_32 <- glm(GainFit ~ Ne_11_14*Ne_15_34 + Ne_11_14*Ne_harmo + Ne_15_34*Ne_harmo, data=Data_GLM, family=binomial)
Mod_GainFit_33 <- glm(GainFit ~ Ne_11_14*Ne_15_34 + Ne_11_14*Ne_inoc + Ne_15_34*Ne_inoc, data=Data_GLM, family=binomial)
Mod_GainFit_34 <- glm(GainFit ~ Ne_11_14*Ne_harmo + Ne_11_14*Ne_inoc + Ne_harmo*Ne_inoc, data=Data_GLM, family=binomial)
Mod_GainFit_35 <- glm(GainFit ~ Ne_15_34*Ne_harmo + Ne_15_34*Ne_inoc + Ne_harmo*Ne_inoc, data=Data_GLM, family=binomial)
```

```
# VIF
#-----------------------------------------------------

## Wi and two Ne estimates

vif(Mod_GainFit_1)
```

```
##             Wi         Ne_1_6        Ne_7_10      Wi:Ne_1_6     Wi:Ne_7_10 
##       450674.4      5126268.0    105011394.1    167780941.8    186105559.7 
## Ne_1_6:Ne_7_10 
##    108750971.3
```

```
vif(Mod_GainFit_2)
```

```
##              Wi          Ne_1_6        Ne_11_14       Wi:Ne_1_6     Wi:Ne_11_14 
##      29126506.0       9387982.4        877307.8     800562360.1     942968581.9 
## Ne_1_6:Ne_11_14 
##      14424470.3
```

```
vif(Mod_GainFit_3)
```

```
##              Wi          Ne_1_6        Ne_15_34       Wi:Ne_1_6     Wi:Ne_15_34 
##         2123087         9517731         2997948        15610211        25172854 
## Ne_1_6:Ne_15_34 
##        11580164
```

```
vif(Mod_GainFit_4)
```

```
##              Wi          Ne_1_6        Ne_harmo       Wi:Ne_1_6     Wi:Ne_harmo 
##        395454.9       3345906.6       3276584.2      85304150.3      93796095.5 
## Ne_1_6:Ne_harmo 
##       8511660.0
```

```
vif(Mod_GainFit_5)
```

```
##             Wi         Ne_1_6        Ne_inoc      Wi:Ne_1_6     Wi:Ne_inoc 
##       9.557076       7.110152     132.933222      12.081314      82.487409 
## Ne_1_6:Ne_inoc 
##      65.972254
```

```
vif(Mod_GainFit_6)
```

```
##               Wi          Ne_7_10         Ne_11_14       Wi:Ne_7_10 
##         703553.3       44772600.6        1614058.5       40863255.7 
##      Wi:Ne_11_14 Ne_7_10:Ne_11_14 
##       43347308.6       60236255.2
```

```
vif(Mod_GainFit_7)
```

```
##               Wi          Ne_7_10         Ne_15_34       Wi:Ne_7_10 
##      18138565919      36895786768      17615143844     168663367247 
##      Wi:Ne_15_34 Ne_7_10:Ne_15_34 
##     254269292676       9716267532
```

```
vif(Mod_GainFit_8)
```

```
##               Wi          Ne_7_10         Ne_harmo       Wi:Ne_7_10 
##         734280.7      691612523.5      197090594.4     2253244584.9 
##      Wi:Ne_harmo Ne_7_10:Ne_harmo 
##     2243451160.2     1444581970.0
```

```
vif(Mod_GainFit_9)
```

```
##              Wi         Ne_7_10         Ne_inoc      Wi:Ne_7_10      Wi:Ne_inoc 
##        75152391      2966796279       177867996      1312861048       928697836 
## Ne_7_10:Ne_inoc 
##      1279521947
```

```
vif(Mod_GainFit_10)
```

```
##                Wi          Ne_11_14          Ne_15_34       Wi:Ne_11_14 
##          361822.6          845126.9         2551100.8        12298696.8 
##       Wi:Ne_15_34 Ne_11_14:Ne_15_34 
##        17014058.5         6016796.7
```

```
vif(Mod_GainFit_11)
```

```
##                Wi          Ne_11_14          Ne_harmo       Wi:Ne_11_14 
##         716193.62          60415.33        2649011.02       55666514.43 
##       Wi:Ne_harmo Ne_11_14:Ne_harmo 
##       51927323.58        5763980.11
```

```
vif(Mod_GainFit_12)
```

```
##               Wi         Ne_11_14          Ne_inoc      Wi:Ne_11_14 
##         6.074368        26.752172       250.681156        87.743076 
##       Wi:Ne_inoc Ne_11_14:Ne_inoc 
##       220.959931        87.908533
```

```
vif(Mod_GainFit_13)
```

```
##                Wi          Ne_15_34          Ne_harmo       Wi:Ne_15_34 
##        15313606.4        33541676.2          704293.3       280088308.0 
##       Wi:Ne_harmo Ne_15_34:Ne_harmo 
##       192133750.4        18301473.8
```

```
vif(Mod_GainFit_14)
```

```
##               Wi         Ne_15_34          Ne_inoc      Wi:Ne_15_34 
##         7.032829         7.586350        15.015710        18.139316 
##       Wi:Ne_inoc Ne_15_34:Ne_inoc 
##        21.883313        25.105360
```

```
vif(Mod_GainFit_15)
```

```
##               Wi         Ne_harmo          Ne_inoc      Wi:Ne_harmo 
##         11.63056         11.00851        147.15798         28.60209 
##       Wi:Ne_inoc Ne_harmo:Ne_inoc 
##         25.76983        166.35322
```

```
## Three Ne estimates

vif(Mod_GainFit_19)
```

```
##          Ne_1_6         Ne_7_10         Ne_inoc  Ne_1_6:Ne_7_10  Ne_1_6:Ne_inoc 
##      268646.787    33159270.882        8890.566    31412687.147     3783637.894 
## Ne_7_10:Ne_inoc 
##     1776661.960
```

```
vif(Mod_GainFit_22)
```

```
##           Ne_1_6         Ne_11_14          Ne_inoc  Ne_1_6:Ne_11_14 
##       1560319137          4741292         41961129        624158510 
##   Ne_1_6:Ne_inoc Ne_11_14:Ne_inoc 
##       3465704189       5597226703
```

```
vif(Mod_GainFit_24)
```

```
##           Ne_1_6         Ne_15_34          Ne_inoc  Ne_1_6:Ne_15_34 
##      38038769.72      13703850.30       5352896.87      81261855.84 
##   Ne_1_6:Ne_inoc Ne_15_34:Ne_inoc 
##      14491884.80         55837.98
```

```
vif(Mod_GainFit_25)
```

```
##           Ne_1_6         Ne_harmo          Ne_inoc  Ne_1_6:Ne_harmo 
##       8614890.77      50404237.36         25619.12     112996649.98 
##   Ne_1_6:Ne_inoc Ne_harmo:Ne_inoc 
##     101779996.12      57964994.97
```

```
vif(Mod_GainFit_28)
```

```
##          Ne_7_10         Ne_11_14          Ne_inoc Ne_7_10:Ne_11_14 
##     19933631.367       367357.725         4150.807     24716589.859 
##  Ne_7_10:Ne_inoc Ne_11_14:Ne_inoc 
##       592378.296      2014281.825
```

```
vif(Mod_GainFit_30)
```

```
##          Ne_7_10         Ne_15_34          Ne_inoc Ne_7_10:Ne_15_34 
##      18170134.92        842451.79          9818.37      26980547.57 
##  Ne_7_10:Ne_inoc Ne_15_34:Ne_inoc 
##       1400177.95       1379434.52
```

```
vif(Mod_GainFit_31)
```

```
##          Ne_7_10         Ne_harmo          Ne_inoc Ne_7_10:Ne_harmo 
##     1850725739.7      247892761.4         202802.1     3271360346.7 
##  Ne_7_10:Ne_inoc Ne_harmo:Ne_inoc 
##       65008374.0       85259752.4
```

```
vif(Mod_GainFit_33)
```

```
##          Ne_11_14          Ne_15_34           Ne_inoc Ne_11_14:Ne_15_34 
##           5930304          22853072          10035175          24547053 
##  Ne_11_14:Ne_inoc  Ne_15_34:Ne_inoc 
##          59657930           2592834
```

```
vif(Mod_GainFit_34)
```

```
##          Ne_11_14          Ne_harmo           Ne_inoc Ne_11_14:Ne_harmo 
##           3503176          52629606           1354038          72629910 
##  Ne_11_14:Ne_inoc  Ne_harmo:Ne_inoc 
##         127775847          44379302
```

```
vif(Mod_GainFit_35)
```

```
##          Ne_15_34          Ne_harmo           Ne_inoc Ne_15_34:Ne_harmo 
##        1051868641        6254613644          47573230        1511003515 
##  Ne_15_34:Ne_inoc  Ne_harmo:Ne_inoc 
##        4030809194        6269796602
```

```
## For some models with only Ne explanatory variables, VIF cannot be calculated because the Ne are highly correlated. The following error appears: “there are aliased coefficients in the model.“

# vif(Mod_GainFit_16)
# vif(Mod_GainFit_17)
# vif(Mod_GainFit_18)
# vif(Mod_GainFit_20)
# vif(Mod_GainFit_21)
# vif(Mod_GainFit_23)
# vif(Mod_GainFit_26)
# vif(Mod_GainFit_27)
# vif(Mod_GainFit_29)
# vif(Mod_GainFit_32)
```

###### VIF 2 variables

**VIF:** If two explanatory variables are retained in
the model, the VIF values are all below 10 for all the models with Wi
and one Ne estimate. However, the VIF values are still high for most
models with two Ne estimates.

```
# Models with 2 explanatory variables
#-----------------------------------------------------

## Wi and one Ne estimate

Mod_GainFit_Wi_Ne_1_6 <- glm(GainFit ~ Wi*Ne_1_6, data=Data_GLM, family=binomial)
Mod_GainFit_Wi_Ne_7_10 <- glm(GainFit ~ Wi*Ne_7_10, data=Data_GLM, family=binomial)
Mod_GainFit_Wi_Ne_11_14 <- glm(GainFit ~ Wi*Ne_11_14, data=Data_GLM, family=binomial)
Mod_GainFit_Wi_Ne_15_34 <- glm(GainFit ~ Wi*Ne_15_34, data=Data_GLM, family=binomial)
Mod_GainFit_Wi_Ne_harmo <- glm(GainFit ~ Wi*Ne_harmo, data=Data_GLM, family=binomial)
Mod_GainFit_Wi_Ne_inoc <- glm(GainFit ~ Wi*Ne_inoc, data=Data_GLM, family=binomial)

## Two Ne estimates

Mod_GainFit_Ne_1_6_Ne_7_10  <- glm(GainFit ~ Ne_1_6*Ne_7_10, data=Data_GLM, family=binomial)
Mod_GainFit_Ne_1_6_Ne_11_14 <- glm(GainFit ~ Ne_1_6*Ne_11_14, data=Data_GLM, family=binomial)
Mod_GainFit_Ne_1_6_Ne_15_34 <- glm(GainFit ~ Ne_1_6*Ne_15_34, data=Data_GLM, family=binomial)
Mod_GainFit_Ne_1_6_Ne_harmo <- glm(GainFit ~ Ne_1_6*Ne_harmo, data=Data_GLM, family=binomial)
Mod_GainFit_Ne_1_6_Ne_inoc <- glm(GainFit ~ Ne_1_6*Ne_inoc, data=Data_GLM, family=binomial)
Mod_GainFit_Ne_7_10_Ne_11_14 <- glm(GainFit ~ Ne_7_10*Ne_11_14, data=Data_GLM, family=binomial)
Mod_GainFit_Ne_7_10_Ne_15_34 <- glm(GainFit ~ Ne_7_10*Ne_15_34, data=Data_GLM, family=binomial)
Mod_GainFit_Ne_7_10_Ne_harmo <- glm(GainFit ~ Ne_7_10*Ne_harmo, data=Data_GLM, family=binomial)
Mod_GainFit_Ne_7_10_Ne_inoc <- glm(GainFit ~ Ne_7_10*Ne_inoc, data=Data_GLM, family=binomial)
Mod_GainFit_Ne_11_14_Ne_15_34 <- glm(GainFit ~ Ne_11_14*Ne_15_34, data=Data_GLM, family=binomial)
Mod_GainFit_Ne_11_14_Ne_harmo <- glm(GainFit ~ Ne_11_14*Ne_harmo, data=Data_GLM, family=binomial)
Mod_GainFit_Ne_11_14_Ne_inoc <- glm(GainFit ~ Ne_11_14*Ne_inoc, data=Data_GLM, family=binomial)
Mod_GainFit_Ne_15_34_Ne_harmo <- glm(GainFit ~ Ne_15_34*Ne_harmo, data=Data_GLM, family=binomial)
Mod_GainFit_Ne_15_34_Ne_inoc <- glm(GainFit ~ Ne_15_34*Ne_inoc, data=Data_GLM, family=binomial)
Mod_GainFit_Ne_harmo_Ne_inoc <- glm(GainFit ~ Ne_harmo*Ne_inoc, data=Data_GLM, family=binomial)
```

###### AIC

**AIC:** Model selection based on Akaike Information
Criterion (AIC). The model including Wi and Ne estimated from 7 to 10
days post inoculation is the one with the lowest AIC
(**67.11**).

```
## Wi and one Ne estimate

Mod_GainFit_Wi_Ne_1_6
```

```
## 
## Call:  glm(formula = GainFit ~ Wi * Ne_1_6, family = binomial, data = Data_GLM)
## 
## Coefficients:
## (Intercept)           Wi       Ne_1_6    Wi:Ne_1_6  
##    2.016496    -3.044263    -0.001962     0.004295  
## 
## Degrees of Freedom: 63 Total (i.e. Null);  60 Residual
## Null Deviance:       88.66 
## Residual Deviance: 78.01     AIC: 86.01
```

```
Mod_GainFit_Wi_Ne_7_10
```

```
## 
## Call:  glm(formula = GainFit ~ Wi * Ne_7_10, family = binomial, data = Data_GLM)
## 
## Coefficients:
## (Intercept)           Wi      Ne_7_10   Wi:Ne_7_10  
##    -1.64031      1.58832      0.01469     -0.01781  
## 
## Degrees of Freedom: 63 Total (i.e. Null);  60 Residual
## Null Deviance:       88.66 
## Residual Deviance: 59.11     AIC: 67.11
```

```
Mod_GainFit_Wi_Ne_11_14
```

```
## 
## Call:  glm(formula = GainFit ~ Wi * Ne_11_14, family = binomial, data = Data_GLM)
## 
## Coefficients:
## (Intercept)           Wi     Ne_11_14  Wi:Ne_11_14  
##     -2.3974       2.3081       0.0146      -0.0180  
## 
## Degrees of Freedom: 63 Total (i.e. Null);  60 Residual
## Null Deviance:       88.66 
## Residual Deviance: 69.54     AIC: 77.54
```

```
Mod_GainFit_Wi_Ne_15_34
```

```
## 
## Call:  glm(formula = GainFit ~ Wi * Ne_15_34, family = binomial, data = Data_GLM)
## 
## Coefficients:
## (Intercept)           Wi     Ne_15_34  Wi:Ne_15_34  
##   -2.173624     3.041130     0.004957    -0.006795  
## 
## Degrees of Freedom: 63 Total (i.e. Null);  60 Residual
## Null Deviance:       88.66 
## Residual Deviance: 70.39     AIC: 78.39
```

```
Mod_GainFit_Wi_Ne_harmo
```

```
## 
## Call:  glm(formula = GainFit ~ Wi * Ne_harmo, family = binomial, data = Data_GLM)
## 
## Coefficients:
## (Intercept)           Wi     Ne_harmo  Wi:Ne_harmo  
##    -2.23561      2.25180      0.01073     -0.01289  
## 
## Degrees of Freedom: 63 Total (i.e. Null);  60 Residual
## Null Deviance:       88.66 
## Residual Deviance: 64.22     AIC: 72.22
```

```
Mod_GainFit_Wi_Ne_inoc
```

```
## 
## Call:  glm(formula = GainFit ~ Wi * Ne_inoc, family = binomial, data = Data_GLM)
## 
## Coefficients:
## (Intercept)           Wi      Ne_inoc   Wi:Ne_inoc  
##     -0.7947       2.0584       0.5632      -0.9817  
## 
## Degrees of Freedom: 63 Total (i.e. Null);  60 Residual
## Null Deviance:       88.66 
## Residual Deviance: 73.06     AIC: 81.06
```

```
## Two Ne estimates

Mod_GainFit_Ne_1_6_Ne_7_10
```

```
## 
## Call:  glm(formula = GainFit ~ Ne_1_6 * Ne_7_10, family = binomial, 
##     data = Data_GLM)
## 
## Coefficients:
##    (Intercept)          Ne_1_6         Ne_7_10  Ne_1_6:Ne_7_10  
##     -3.362e-03      -1.718e-03      -1.264e-02       5.327e-05  
## 
## Degrees of Freedom: 63 Total (i.e. Null);  60 Residual
## Null Deviance:       88.66 
## Residual Deviance: 72.42     AIC: 80.42
```

```
Mod_GainFit_Ne_1_6_Ne_11_14
```

```
## 
## Call:  glm(formula = GainFit ~ Ne_1_6 * Ne_11_14, family = binomial, 
##     data = Data_GLM)
## 
## Coefficients:
##     (Intercept)           Ne_1_6         Ne_11_14  Ne_1_6:Ne_11_14  
##      -4.223e-01       -3.949e-03       -6.454e-04        2.374e-05  
## 
## Degrees of Freedom: 63 Total (i.e. Null);  60 Residual
## Null Deviance:       88.66 
## Residual Deviance: 74.12     AIC: 82.12
```

```
Mod_GainFit_Ne_1_6_Ne_15_34
```

```
## 
## Call:  glm(formula = GainFit ~ Ne_1_6 * Ne_15_34, family = binomial, 
##     data = Data_GLM)
## 
## Coefficients:
##     (Intercept)           Ne_1_6         Ne_15_34  Ne_1_6:Ne_15_34  
##       5.582e-02       -1.839e-03       -5.368e-04        4.336e-06  
## 
## Degrees of Freedom: 63 Total (i.e. Null);  60 Residual
## Null Deviance:       88.66 
## Residual Deviance: 87.89     AIC: 95.89
```

```
Mod_GainFit_Ne_1_6_Ne_harmo
```

```
## 
## Call:  glm(formula = GainFit ~ Ne_1_6 * Ne_harmo, family = binomial, 
##     data = Data_GLM)
## 
## Coefficients:
##     (Intercept)           Ne_1_6         Ne_harmo  Ne_1_6:Ne_harmo  
##      -2.359e-01       -3.821e-03       -5.577e-03        3.065e-05  
## 
## Degrees of Freedom: 63 Total (i.e. Null);  60 Residual
## Null Deviance:       88.66 
## Residual Deviance: 75.41     AIC: 83.41
```

```
Mod_GainFit_Ne_1_6_Ne_inoc
```

```
## 
## Call:  glm(formula = GainFit ~ Ne_1_6 * Ne_inoc, family = binomial, 
##     data = Data_GLM)
## 
## Coefficients:
##    (Intercept)          Ne_1_6         Ne_inoc  Ne_1_6:Ne_inoc  
##       0.947717       -0.002614       -0.706694        0.002177  
## 
## Degrees of Freedom: 63 Total (i.e. Null);  60 Residual
## Null Deviance:       88.66 
## Residual Deviance: 73.68     AIC: 81.68
```

```
Mod_GainFit_Ne_7_10_Ne_11_14
```

```
## 
## Call:  glm(formula = GainFit ~ Ne_7_10 * Ne_11_14, family = binomial, 
##     data = Data_GLM)
## 
## Coefficients:
##      (Intercept)           Ne_7_10          Ne_11_14  Ne_7_10:Ne_11_14  
##        0.7787368        -0.0232308        -0.0120729         0.0001204  
## 
## Degrees of Freedom: 63 Total (i.e. Null);  60 Residual
## Null Deviance:       88.66 
## Residual Deviance: 72.22     AIC: 80.22
```

```
Mod_GainFit_Ne_7_10_Ne_15_34
```

```
## 
## Call:  glm(formula = GainFit ~ Ne_7_10 * Ne_15_34, family = binomial, 
##     data = Data_GLM)
## 
## Coefficients:
##      (Intercept)           Ne_7_10          Ne_15_34  Ne_7_10:Ne_15_34  
##       -2.585e+00         4.012e-02         3.032e-03        -3.845e-05  
## 
## Degrees of Freedom: 63 Total (i.e. Null);  60 Residual
## Null Deviance:       88.66 
## Residual Deviance: 71.96     AIC: 79.96
```

```
Mod_GainFit_Ne_7_10_Ne_harmo
```

```
## 
## Call:  glm(formula = GainFit ~ Ne_7_10 * Ne_harmo, family = binomial, 
##     data = Data_GLM)
## 
## Coefficients:
##      (Intercept)           Ne_7_10          Ne_harmo  Ne_7_10:Ne_harmo  
##       -5.6229327         0.1161428         0.0187876        -0.0002521  
## 
## Degrees of Freedom: 63 Total (i.e. Null);  60 Residual
## Null Deviance:       88.66 
## Residual Deviance: 73.69     AIC: 81.69
```

```
Mod_GainFit_Ne_7_10_Ne_inoc
```

```
## 
## Call:  glm(formula = GainFit ~ Ne_7_10 * Ne_inoc, family = binomial, 
##     data = Data_GLM)
## 
## Coefficients:
##     (Intercept)          Ne_7_10          Ne_inoc  Ne_7_10:Ne_inoc  
##       -1.504236         0.006125         0.493406        -0.001419  
## 
## Degrees of Freedom: 63 Total (i.e. Null);  60 Residual
## Null Deviance:       88.66 
## Residual Deviance: 79.42     AIC: 87.42
```

```
Mod_GainFit_Ne_11_14_Ne_15_34
```

```
## 
## Call:  glm(formula = GainFit ~ Ne_11_14 * Ne_15_34, family = binomial, 
##     data = Data_GLM)
## 
## Coefficients:
##       (Intercept)           Ne_11_14           Ne_15_34  Ne_11_14:Ne_15_34  
##        -1.250e+00          1.036e-02         -2.493e-04         -4.105e-06  
## 
## Degrees of Freedom: 63 Total (i.e. Null);  60 Residual
## Null Deviance:       88.66 
## Residual Deviance: 76.09     AIC: 84.09
```

```
Mod_GainFit_Ne_11_14_Ne_harmo
```

```
## 
## Call:  glm(formula = GainFit ~ Ne_11_14 * Ne_harmo, family = binomial, 
##     data = Data_GLM)
## 
## Coefficients:
##       (Intercept)           Ne_11_14           Ne_harmo  Ne_11_14:Ne_harmo  
##        -3.529e-01         -2.413e-03         -4.626e-03          2.669e-05  
## 
## Degrees of Freedom: 63 Total (i.e. Null);  60 Residual
## Null Deviance:       88.66 
## Residual Deviance: 75.32     AIC: 83.32
```

```
Mod_GainFit_Ne_11_14_Ne_inoc
```

```
## 
## Call:  glm(formula = GainFit ~ Ne_11_14 * Ne_inoc, family = binomial, 
##     data = Data_GLM)
## 
## Coefficients:
##      (Intercept)          Ne_11_14           Ne_inoc  Ne_11_14:Ne_inoc  
##       -0.6162534         0.0064643        -0.4477617         0.0009079  
## 
## Degrees of Freedom: 63 Total (i.e. Null);  60 Residual
## Null Deviance:       88.66 
## Residual Deviance: 71.62     AIC: 79.62
```

```
Mod_GainFit_Ne_15_34_Ne_harmo
```

```
## 
## Call:  glm(formula = GainFit ~ Ne_15_34 * Ne_harmo, family = binomial, 
##     data = Data_GLM)
## 
## Coefficients:
##       (Intercept)           Ne_15_34           Ne_harmo  Ne_15_34:Ne_harmo  
##        -1.155e+00         -1.485e-03          1.570e-02         -9.513e-06  
## 
## Degrees of Freedom: 63 Total (i.e. Null);  60 Residual
## Null Deviance:       88.66 
## Residual Deviance: 74.74     AIC: 82.74
```

```
Mod_GainFit_Ne_15_34_Ne_inoc
```

```
## 
## Call:  glm(formula = GainFit ~ Ne_15_34 * Ne_inoc, family = binomial, 
##     data = Data_GLM)
## 
## Coefficients:
##      (Intercept)          Ne_15_34           Ne_inoc  Ne_15_34:Ne_inoc  
##        -2.781144          0.003985          1.292357         -0.001504  
## 
## Degrees of Freedom: 63 Total (i.e. Null);  60 Residual
## Null Deviance:       88.66 
## Residual Deviance: 64.77     AIC: 72.77
```

```
Mod_GainFit_Ne_harmo_Ne_inoc
```

```
## 
## Call:  glm(formula = GainFit ~ Ne_harmo * Ne_inoc, family = binomial, 
##     data = Data_GLM)
## 
## Coefficients:
##      (Intercept)          Ne_harmo           Ne_inoc  Ne_harmo:Ne_inoc  
##        -2.254904          0.007517          0.936374         -0.002234  
## 
## Degrees of Freedom: 63 Total (i.e. Null);  60 Residual
## Null Deviance:       88.66 
## Residual Deviance: 79.01     AIC: 87.01
```

```
# VIF
#-----------------------------------------------------

## Wi and one Ne estimate

vif(Mod_GainFit_Wi_Ne_1_6)
```

```
##        Wi    Ne_1_6 Wi:Ne_1_6 
##  2.595276  1.629835  1.780865
```

```
vif(Mod_GainFit_Wi_Ne_7_10)
```

```
##         Wi    Ne_7_10 Wi:Ne_7_10 
##   1.748848   5.354663   6.647752
```

```
vif(Mod_GainFit_Wi_Ne_11_14)
```

```
##          Wi    Ne_11_14 Wi:Ne_11_14 
##    4.457415    3.697381    5.993043
```

```
vif(Mod_GainFit_Wi_Ne_15_34)
```

```
##          Wi    Ne_15_34 Wi:Ne_15_34 
##    3.895287    4.194234    7.994127
```

```
vif(Mod_GainFit_Wi_Ne_harmo)
```

```
##          Wi    Ne_harmo Wi:Ne_harmo 
##    2.365392    3.722981    5.160847
```

```
vif(Mod_GainFit_Wi_Ne_inoc)
```

```
##         Wi    Ne_inoc Wi:Ne_inoc 
##   3.957098   5.137906  10.043245
```

```
## Two Ne estimates

vif(Mod_GainFit_Ne_1_6_Ne_7_10)
```

```
##         Ne_1_6        Ne_7_10 Ne_1_6:Ne_7_10 
##       1.761645      18.494489      17.717219
```

```
vif(Mod_GainFit_Ne_1_6_Ne_11_14)
```

```
##          Ne_1_6        Ne_11_14 Ne_1_6:Ne_11_14 
##       10.134219        5.229622       17.079652
```

```
vif(Mod_GainFit_Ne_1_6_Ne_15_34)
```

```
##          Ne_1_6        Ne_15_34 Ne_1_6:Ne_15_34 
##       25.420748        9.095166       32.192368
```

```
vif(Mod_GainFit_Ne_1_6_Ne_harmo)
```

```
##          Ne_1_6        Ne_harmo Ne_1_6:Ne_harmo 
##        4.718638        6.136987        9.668501
```

```
vif(Mod_GainFit_Ne_1_6_Ne_inoc)
```

```
##         Ne_1_6        Ne_inoc Ne_1_6:Ne_inoc 
##       2.002779       7.071381       7.497709
```

```
vif(Mod_GainFit_Ne_7_10_Ne_11_14)
```

```
##          Ne_7_10         Ne_11_14 Ne_7_10:Ne_11_14 
##        183.64616         32.37956        295.95764
```

```
vif(Mod_GainFit_Ne_7_10_Ne_15_34)
```

```
##          Ne_7_10         Ne_15_34 Ne_7_10:Ne_15_34 
##       488.376939         8.483313       498.780761
```

```
vif(Mod_GainFit_Ne_7_10_Ne_harmo)
```

```
##          Ne_7_10         Ne_harmo Ne_7_10:Ne_harmo 
##       1127.32523         26.10994       1419.19111
```

```
vif(Mod_GainFit_Ne_7_10_Ne_inoc)
```

```
##         Ne_7_10         Ne_inoc Ne_7_10:Ne_inoc 
##        2.944032        9.642183       14.390579
```

```
vif(Mod_GainFit_Ne_11_14_Ne_15_34)
```

```
##          Ne_11_14          Ne_15_34 Ne_11_14:Ne_15_34 
##          8.315505          8.378103         22.765026
```

```
vif(Mod_GainFit_Ne_11_14_Ne_harmo)
```

```
##          Ne_11_14          Ne_harmo Ne_11_14:Ne_harmo 
##          21.44522           5.74508          34.16677
```

```
vif(Mod_GainFit_Ne_11_14_Ne_inoc)
```

```
##         Ne_11_14          Ne_inoc Ne_11_14:Ne_inoc 
##         5.274368         6.859732        15.070106
```

```
vif(Mod_GainFit_Ne_15_34_Ne_harmo)
```

```
##          Ne_15_34          Ne_harmo Ne_15_34:Ne_harmo 
##          28.57210          24.51426          54.77507
```

```
vif(Mod_GainFit_Ne_15_34_Ne_inoc)
```

```
##         Ne_15_34          Ne_inoc Ne_15_34:Ne_inoc 
##         2.672197        17.840866        23.603225
```

```
vif(Mod_GainFit_Ne_harmo_Ne_inoc)
```

```
##         Ne_harmo          Ne_inoc Ne_harmo:Ne_inoc 
##         4.335721        38.068398        52.578877
```

###### Model fit

**Model Fit:** Among the models that retain two
explanatory variables, the one with the highest McFadden’s R-squared is
the one with Wi and Ne estimated from 7 to 10 days post inoculation
(**McFadden’s R-squared = 0.3332621**). Moreover, the
Hosmer and Lemeshow goodness of fit test indicates that the logistic
regression is a good fit to the observed data (p=0.37).

```
# McFadden's R-squared
#-----------------------------------------------------

## Wi and one Ne estimate

pR2(Mod_GainFit_Wi_Ne_1_6)
```

```
## fitting null model for pseudo-r2
```

```
##         llh     llhNull          G2    McFadden        r2ML        r2CU 
## -39.0060176 -44.3301645  10.6482938   0.1201021   0.1532752   0.2044336
```

```
pR2(Mod_GainFit_Wi_Ne_7_10)
```

```
## fitting null model for pseudo-r2
```

```
##         llh     llhNull          G2    McFadden        r2ML        r2CU 
## -29.5566000 -44.3301645  29.5471289   0.3332621   0.3697722   0.4931902
```

```
pR2(Mod_GainFit_Wi_Ne_11_14)
```

```
## fitting null model for pseudo-r2
```

```
##         llh     llhNull          G2    McFadden        r2ML        r2CU 
## -34.7684229 -44.3301645  19.1234831   0.2156938   0.2582955   0.3445063
```

```
pR2(Mod_GainFit_Wi_Ne_15_34)
```

```
## fitting null model for pseudo-r2
```

```
##         llh     llhNull          G2    McFadden        r2ML        r2CU 
## -35.1926400 -44.3301645  18.2750490   0.2061243   0.2483975   0.3313045
```

```
pR2(Mod_GainFit_Wi_Ne_harmo)
```

```
## fitting null model for pseudo-r2
```

```
##         llh     llhNull          G2    McFadden        r2ML        r2CU 
## -32.1114624 -44.3301645  24.4374041   0.2756295   0.3173919   0.4233271
```

```
pR2(Mod_GainFit_Wi_Ne_inoc)
```

```
## fitting null model for pseudo-r2
```

```
##         llh     llhNull          G2    McFadden        r2ML        r2CU 
## -36.5282639 -44.3301645  15.6038012   0.1759953   0.2163630   0.2885780
```

```
## Two Ne estimates

pR2(Mod_GainFit_Ne_1_6_Ne_7_10)
```

```
## fitting null model for pseudo-r2
```

```
##         llh     llhNull          G2    McFadden        r2ML        r2CU 
## -36.2088069 -44.3301645  16.2427151   0.1832016   0.2241472   0.2989603
```

```
pR2(Mod_GainFit_Ne_1_6_Ne_11_14)
```

```
## fitting null model for pseudo-r2
```

```
##         llh     llhNull          G2    McFadden        r2ML        r2CU 
## -37.0590536 -44.3301645  14.5422218   0.1640217   0.2032563   0.2710967
```

```
pR2(Mod_GainFit_Ne_1_6_Ne_15_34)
```

```
## fitting null model for pseudo-r2
```

```
##           llh       llhNull            G2      McFadden          r2ML 
## -43.943972071 -44.330164468   0.772384794   0.008711729   0.011995980 
##          r2CU 
##   0.015999852
```

```
pR2(Mod_GainFit_Ne_1_6_Ne_harmo)
```

```
## fitting null model for pseudo-r2
```

```
##         llh     llhNull          G2    McFadden        r2ML        r2CU 
## -37.7026620 -44.3301645  13.2550049   0.1495032   0.1870693   0.2495070
```

```
pR2(Mod_GainFit_Ne_1_6_Ne_inoc)
```

```
## fitting null model for pseudo-r2
```

```
##         llh     llhNull          G2    McFadden        r2ML        r2CU 
## -36.8389070 -44.3301645  14.9825150   0.1689878   0.2087187   0.2783823
```

```
pR2(Mod_GainFit_Ne_7_10_Ne_11_14)
```

```
## fitting null model for pseudo-r2
```

```
##         llh     llhNull          G2    McFadden        r2ML        r2CU 
## -36.1103546 -44.3301645  16.4396197   0.1854225   0.2265305   0.3021391
```

```
pR2(Mod_GainFit_Ne_7_10_Ne_15_34)
```

```
## fitting null model for pseudo-r2
```

```
##         llh     llhNull          G2    McFadden        r2ML        r2CU 
## -35.9807666 -44.3301645  16.6987958   0.1883457   0.2296564   0.3063084
```

```
pR2(Mod_GainFit_Ne_7_10_Ne_harmo)
```

```
## fitting null model for pseudo-r2
```

```
##         llh     llhNull          G2    McFadden        r2ML        r2CU 
## -36.8452141 -44.3301645  14.9699008   0.1688455   0.2085628   0.2781743
```

```
pR2(Mod_GainFit_Ne_7_10_Ne_inoc)
```

```
## fitting null model for pseudo-r2
```

```
##         llh     llhNull          G2    McFadden        r2ML        r2CU 
## -39.7092673 -44.3301645   9.2417943   0.1042382   0.1344612   0.1793400
```

```
pR2(Mod_GainFit_Ne_11_14_Ne_15_34)
```

```
## fitting null model for pseudo-r2
```

```
##         llh     llhNull          G2    McFadden        r2ML        r2CU 
## -38.0448128 -44.3301645  12.5707034   0.1417850   0.1783307   0.2378517
```

```
pR2(Mod_GainFit_Ne_11_14_Ne_harmo)
```

```
## fitting null model for pseudo-r2
```

```
##         llh     llhNull          G2    McFadden        r2ML        r2CU 
## -37.6585145 -44.3301645  13.3433000   0.1504991   0.1881901   0.2510019
```

```
pR2(Mod_GainFit_Ne_11_14_Ne_inoc)
```

```
## fitting null model for pseudo-r2
```

```
##         llh     llhNull          G2    McFadden        r2ML        r2CU 
## -35.8117857 -44.3301645  17.0367575   0.1921576   0.2337136   0.3117197
```

```
pR2(Mod_GainFit_Ne_15_34_Ne_harmo)
```

```
## fitting null model for pseudo-r2
```

```
##         llh     llhNull          G2    McFadden        r2ML        r2CU 
## -37.3686083 -44.3301645  13.9231124   0.1570388   0.1955115   0.2607670
```

```
pR2(Mod_GainFit_Ne_15_34_Ne_inoc)
```

```
## fitting null model for pseudo-r2
```

```
##         llh     llhNull          G2    McFadden        r2ML        r2CU 
## -32.3861308 -44.3301645  23.8880672   0.2694336   0.3115076   0.4154788
```

```
pR2(Mod_GainFit_Ne_harmo_Ne_inoc)
```

```
## fitting null model for pseudo-r2
```

```
##         llh     llhNull          G2    McFadden        r2ML        r2CU 
## -39.5036384 -44.3301645   9.6530521   0.1088768   0.1400052   0.1867344
```

```
# Hosmer and Lemeshow test
#-----------------------------------------------------

hoslem.test(Mod_GainFit_Wi_Ne_7_10$y,fitted(Mod_GainFit_Wi_Ne_7_10), g=8)
```

```
## 
##  Hosmer and Lemeshow goodness of fit (GOF) test
## 
## data:  Mod_GainFit_Wi_Ne_7_10$y, fitted(Mod_GainFit_Wi_Ne_7_10)
## X-squared = 6.4958, df = 6, p-value = 0.37
```

###### Model selection

**Model selection:** The model with the lowest AIC is
the full model (Wi, Ne\_7\_10 and Wi\*Ne\_7\_10).

```
# Model selection with all the lineages
#----------------------------------------------------

options(na.action = "na.fail")
dredge(Mod_GainFit_Wi_Ne_7_10)
```

```
## Global model call: glm(formula = GainFit ~ Wi * Ne_7_10, family = binomial, data = Data_GLM)
## ---
## Model selection table 
##      (Int)  Ne_7_10     Wi Ne_7_10:Wi df  logLik AICc delta weight
## 8 -1.64000 0.014690  1.588   -0.01781  4 -29.557 67.8  0.00      1
## 4  0.07657 0.003868 -1.925             3 -38.553 83.5 15.72      0
## 3  0.77370          -1.280             2 -42.022 88.2 20.45      0
## 2 -0.54190 0.002262                    2 -42.765 89.7 21.94      0
## 1  0.06252                             1 -44.330 90.7 22.93      0
## Models ranked by AICc(x)
```

```
summary(Mod_GainFit_Wi_Ne_7_10)
```

```
## 
## Call:
## glm(formula = GainFit ~ Wi * Ne_7_10, family = binomial, data = Data_GLM)
## 
## Deviance Residuals: 
##     Min       1Q   Median       3Q      Max  
## -1.6288  -0.9018   0.3226   0.7854   2.7189  
## 
## Coefficients:
##              Estimate Std. Error z value Pr(>|z|)    
## (Intercept) -1.640310   0.737152  -2.225 0.026068 *  
## Wi           1.588320   1.074592   1.478 0.139390    
## Ne_7_10      0.014688   0.003926   3.741 0.000183 ***
## Wi:Ne_7_10  -0.017812   0.005412  -3.291 0.000999 ***
## ---
## Signif. codes:  0 '***' 0.001 '**' 0.01 '*' 0.05 '.' 0.1 ' ' 1
## 
## (Dispersion parameter for binomial family taken to be 1)
## 
##     Null deviance: 88.660  on 63  degrees of freedom
## Residual deviance: 59.113  on 60  degrees of freedom
## AIC: 67.113
## 
## Number of Fisher Scoring iterations: 5
```

```
# Model selection without the 9 extincted lineages
#----------------------------------------------------

Mod_GainFit_Wi_Ne_7_10_NoExt <- glm(GainFit ~ Wi*Ne_7_10, data=DataNoExtinction, family=binomial)

options(na.action = "na.fail")
dredge(Mod_GainFit_Wi_Ne_7_10_NoExt)
```

```
## Global model call: glm(formula = GainFit ~ Wi * Ne_7_10, family = binomial, data = DataNoExtinction)
## ---
## Model selection table 
##    (Int)   Ne_7_10      Wi Ne_7_10:Wi df  logLik AICc delta weight
## 8 0.1048 0.0094840 -0.1116   -0.01238  4 -25.417 59.6  0.00  0.708
## 3 2.3430           -2.9390             2 -28.904 62.0  2.40  0.213
## 4 2.0790 0.0008586 -2.9460             3 -28.779 64.0  4.39  0.079
## 1 0.4055                               1 -37.016 76.1 16.47  0.000
## 2 0.3398 0.0002141                     2 -37.005 78.2 18.61  0.000
## Models ranked by AICc(x)
```

```
summary(Mod_GainFit_Wi_Ne_7_10_NoExt)
```

```
## 
## Call:
## glm(formula = GainFit ~ Wi * Ne_7_10, family = binomial, data = DataNoExtinction)
## 
## Deviance Residuals: 
##     Min       1Q   Median       3Q      Max  
## -1.9009  -1.1025   0.3882   0.5989   2.5673  
## 
## Coefficients:
##              Estimate Std. Error z value Pr(>|z|)  
## (Intercept)  0.104785   1.007238   0.104   0.9171  
## Wi          -0.111601   1.284905  -0.087   0.9308  
## Ne_7_10      0.009484   0.004061   2.335   0.0195 *
## Wi:Ne_7_10  -0.012379   0.005367  -2.306   0.0211 *
## ---
## Signif. codes:  0 '***' 0.001 '**' 0.01 '*' 0.05 '.' 0.1 ' ' 1
## 
## (Dispersion parameter for binomial family taken to be 1)
## 
##     Null deviance: 74.031  on 54  degrees of freedom
## Residual deviance: 50.834  on 51  degrees of freedom
## AIC: 58.834
## 
## Number of Fisher Scoring iterations: 5
```

###### Effect plot

```
# Effect plot with all the lineages
#----------------------------------------------------

visreg(Mod_GainFit_Wi_Ne_7_10, "Wi", by="Ne_7_10", breaks=c(50,500),strip.names=c("Ne=50","Ne=500"),
       points=list(cex=1,pch=19),ylab="Probability of replicative fitness gain",xlab="Replicative fitness of the initially inoculated virus (Wi)",scale = "response",rug=2,jitter=TRUE)
```

```
# Effect plot without the 9 extincted lineages
#----------------------------------------------------

visreg(Mod_GainFit_Wi_Ne_7_10_NoExt, "Wi", by="Ne_7_10", breaks=c(50,500),strip.names=c("Ne=50","Ne=500"),
       points=list(cex=1,pch=19),ylab="Probability of replicative fitness gain",xlab="Replicative fitness of the initially inoculated virus (Wi)",scale = "response",rug=2,jitter=TRUE)
```

##### *1.3.2 Final replicative fitness (Wf)*

We assumed a normal distribution for the final replicative
fitness.

###### VIF 3 variables

**VIF:** If three explanatory variables are retained in
the model, the Variance Inflation Factors (Vif) values are extremely
high, because as we have seen, several explanatory variables are highly
correlated.

```
# Models with 3 explanatory variables
#-----------------------------------------------------

## Wi and two Ne estimates

Mod_Wf_1 <- glm(Wf ~ Wi*Ne_1_6 + Wi*Ne_7_10 + Ne_1_6*Ne_7_10, data=DataNoExtinction, family=gaussian)
Mod_Wf_2 <- glm(Wf ~ Wi*Ne_1_6 + Wi*Ne_11_14 + Ne_1_6*Ne_11_14, data=DataNoExtinction, family=gaussian)
Mod_Wf_3 <- glm(Wf ~ Wi*Ne_1_6 + Wi*Ne_15_34 + Ne_1_6*Ne_15_34, data=DataNoExtinction, family=gaussian)
Mod_Wf_4 <- glm(Wf ~ Wi*Ne_1_6 + Wi*Ne_harmo + Ne_1_6*Ne_harmo, data=DataNoExtinction, family=gaussian)
Mod_Wf_5 <- glm(Wf ~ Wi*Ne_1_6 + Wi*Ne_inoc + Ne_1_6*Ne_inoc, data=DataNoExtinction, family=gaussian)
Mod_Wf_6 <- glm(Wf ~ Wi*Ne_7_10 + Wi*Ne_11_14 + Ne_7_10*Ne_11_14, data=DataNoExtinction, family=gaussian)
Mod_Wf_7 <- glm(Wf ~ Wi*Ne_7_10 + Wi*Ne_15_34 + Ne_7_10*Ne_15_34, data=DataNoExtinction, family=gaussian)
Mod_Wf_8 <- glm(Wf ~ Wi*Ne_7_10 + Wi*Ne_harmo + Ne_7_10*Ne_harmo, data=DataNoExtinction, family=gaussian)
Mod_Wf_9 <- glm(Wf ~ Wi*Ne_7_10 + Wi*Ne_inoc + Ne_7_10*Ne_inoc, data=DataNoExtinction, family=gaussian)
Mod_Wf_10 <- glm(Wf ~ Wi*Ne_11_14 + Wi*Ne_15_34 + Ne_11_14*Ne_15_34, data=DataNoExtinction, family=gaussian)
Mod_Wf_11 <- glm(Wf ~ Wi*Ne_11_14 + Wi*Ne_harmo + Ne_11_14*Ne_harmo, data=DataNoExtinction, family=gaussian)
Mod_Wf_12 <- glm(Wf ~ Wi*Ne_11_14 + Wi*Ne_inoc + Ne_11_14*Ne_inoc, data=DataNoExtinction, family=gaussian)
Mod_Wf_13 <- glm(Wf ~ Wi*Ne_15_34 + Wi*Ne_harmo + Ne_15_34*Ne_harmo, data=DataNoExtinction, family=gaussian)
Mod_Wf_14 <- glm(Wf ~ Wi*Ne_15_34 + Wi*Ne_inoc + Ne_15_34*Ne_inoc, data=DataNoExtinction, family=gaussian)
Mod_Wf_15 <- glm(Wf ~ Wi*Ne_harmo + Wi*Ne_inoc + Ne_harmo*Ne_inoc, data=DataNoExtinction, family=gaussian)

## Three Ne estimates

Mod_Wf_16 <- glm(Wf ~ Ne_1_6*Ne_7_10 + Ne_1_6*Ne_11_14 + Ne_7_10*Ne_11_14, data=DataNoExtinction, family=gaussian)
Mod_Wf_17 <- glm(Wf ~ Ne_1_6*Ne_7_10 + Ne_1_6*Ne_15_34 + Ne_7_10*Ne_15_34, data=DataNoExtinction, family=gaussian)
Mod_Wf_18 <- glm(Wf ~ Ne_1_6*Ne_7_10 + Ne_1_6*Ne_harmo + Ne_7_10*Ne_harmo, data=DataNoExtinction, family=gaussian)
Mod_Wf_19 <- glm(Wf ~ Ne_1_6*Ne_7_10 + Ne_1_6*Ne_inoc + Ne_7_10*Ne_inoc, data=DataNoExtinction, family=gaussian)
Mod_Wf_20 <- glm(Wf ~ Ne_1_6*Ne_11_14 + Ne_1_6*Ne_15_34 + Ne_11_14*Ne_15_34, data=DataNoExtinction, family=gaussian)
Mod_Wf_21 <- glm(Wf ~ Ne_1_6*Ne_11_14 + Ne_1_6*Ne_harmo + Ne_11_14*Ne_harmo, data=DataNoExtinction, family=gaussian)
Mod_Wf_22 <- glm(Wf ~ Ne_1_6*Ne_11_14 + Ne_1_6*Ne_inoc + Ne_11_14*Ne_inoc, data=DataNoExtinction, family=gaussian)
Mod_Wf_23 <- glm(Wf ~ Ne_1_6*Ne_15_34 + Ne_1_6*Ne_harmo + Ne_15_34*Ne_harmo, data=DataNoExtinction, family=gaussian)
Mod_Wf_24 <- glm(Wf ~ Ne_1_6*Ne_15_34 + Ne_1_6*Ne_inoc + Ne_15_34*Ne_inoc, data=DataNoExtinction, family=gaussian)
Mod_Wf_25 <- glm(Wf ~ Ne_1_6*Ne_harmo + Ne_1_6*Ne_inoc + Ne_harmo*Ne_inoc, data=DataNoExtinction, family=gaussian)
Mod_Wf_26 <- glm(Wf ~ Ne_7_10*Ne_11_14 + Ne_7_10*Ne_15_34 + Ne_11_14*Ne_15_34, data=DataNoExtinction, family=gaussian)
Mod_Wf_27 <- glm(Wf ~ Ne_7_10*Ne_11_14 + Ne_7_10*Ne_harmo + Ne_11_14*Ne_harmo, data=DataNoExtinction, family=gaussian)
Mod_Wf_28 <- glm(Wf ~ Ne_7_10*Ne_11_14 + Ne_7_10*Ne_inoc + Ne_11_14*Ne_inoc, data=DataNoExtinction, family=gaussian)
Mod_Wf_29 <- glm(Wf ~ Ne_7_10*Ne_15_34 + Ne_7_10*Ne_harmo + Ne_15_34*Ne_harmo, data=DataNoExtinction, family=gaussian)
Mod_Wf_30 <- glm(Wf ~ Ne_7_10*Ne_15_34 + Ne_7_10*Ne_inoc + Ne_15_34*Ne_inoc, data=DataNoExtinction, family=gaussian)
Mod_Wf_31 <- glm(Wf ~ Ne_7_10*Ne_harmo + Ne_7_10*Ne_inoc + Ne_harmo*Ne_inoc, data=DataNoExtinction, family=gaussian)
Mod_Wf_32 <- glm(Wf ~ Ne_11_14*Ne_15_34 + Ne_11_14*Ne_harmo + Ne_15_34*Ne_harmo, data=DataNoExtinction, family=gaussian)
Mod_Wf_33 <- glm(Wf ~ Ne_11_14*Ne_15_34 + Ne_11_14*Ne_inoc + Ne_15_34*Ne_inoc, data=DataNoExtinction, family=gaussian)
Mod_Wf_34 <- glm(Wf ~ Ne_11_14*Ne_harmo + Ne_11_14*Ne_inoc + Ne_harmo*Ne_inoc, data=DataNoExtinction, family=gaussian)
Mod_Wf_35 <- glm(Wf ~ Ne_15_34*Ne_harmo + Ne_15_34*Ne_inoc + Ne_harmo*Ne_inoc, data=DataNoExtinction, family=gaussian)
```

```
# VIF
#-----------------------------------------------------

## Wi and two Ne estimates

vif(Mod_Wf_1)
```

```
##             Wi         Ne_1_6        Ne_7_10      Wi:Ne_1_6     Wi:Ne_7_10 
##      11.361535       4.550733     117.728274     497.868000     610.565271 
## Ne_1_6:Ne_7_10 
##     173.843304
```

```
vif(Mod_Wf_2)
```

```
##              Wi          Ne_1_6        Ne_11_14       Wi:Ne_1_6     Wi:Ne_11_14 
##        19.50706        29.66073        57.85672       984.24119      1057.33355 
## Ne_1_6:Ne_11_14 
##       136.95721
```

```
vif(Mod_Wf_3)
```

```
##              Wi          Ne_1_6        Ne_15_34       Wi:Ne_1_6     Wi:Ne_15_34 
##       13.567738       14.148406       14.662123        5.806862       26.618268 
## Ne_1_6:Ne_15_34 
##       31.518910
```

```
vif(Mod_Wf_4)
```

```
##              Wi          Ne_1_6        Ne_harmo       Wi:Ne_1_6     Wi:Ne_harmo 
##        8.119410        6.606865       23.021869       43.102251       66.143749 
## Ne_1_6:Ne_harmo 
##       31.673923
```

```
vif(Mod_Wf_5)
```

```
##             Wi         Ne_1_6        Ne_inoc      Wi:Ne_1_6     Wi:Ne_inoc 
##       8.121835       3.116057      84.839906       8.761171      47.550470 
## Ne_1_6:Ne_inoc 
##      56.978864
```

```
vif(Mod_Wf_6)
```

```
##               Wi          Ne_7_10         Ne_11_14       Wi:Ne_7_10 
##         77.96378        655.65314         28.15955       3684.81192 
##      Wi:Ne_11_14 Ne_7_10:Ne_11_14 
##       4367.28025       1273.35401
```

```
vif(Mod_Wf_7)
```

```
##               Wi          Ne_7_10         Ne_15_34       Wi:Ne_7_10 
##         678494.1        1369624.7         562633.7        9399645.8 
##      Wi:Ne_15_34 Ne_7_10:Ne_15_34 
##       14842092.7         348998.0
```

```
vif(Mod_Wf_8)
```

```
##               Wi          Ne_7_10         Ne_harmo       Wi:Ne_7_10 
##         14.12828       1796.68351        193.10723      10571.83653 
##      Wi:Ne_harmo Ne_7_10:Ne_harmo 
##      10736.94044       1250.92428
```

```
vif(Mod_Wf_9)
```

```
##              Wi         Ne_7_10         Ne_inoc      Wi:Ne_7_10      Wi:Ne_inoc 
##        9.124803        5.721879        9.401542        9.842180       27.653775 
## Ne_7_10:Ne_inoc 
##       22.324056
```

```
vif(Mod_Wf_10)
```

```
##                Wi          Ne_11_14          Ne_15_34       Wi:Ne_11_14 
##          7.930458         92.732279         13.424578        106.994809 
##       Wi:Ne_15_34 Ne_11_14:Ne_15_34 
##        150.053824         60.741815
```

```
vif(Mod_Wf_11)
```

```
##                Wi          Ne_11_14          Ne_harmo       Wi:Ne_11_14 
##          10.10624         160.23870          20.48103          62.19351 
##       Wi:Ne_harmo Ne_11_14:Ne_harmo 
##          58.85866         145.74898
```

```
vif(Mod_Wf_12)
```

```
##               Wi         Ne_11_14          Ne_inoc      Wi:Ne_11_14 
##         6.391021        47.894871       275.588415        85.155869 
##       Wi:Ne_inoc Ne_11_14:Ne_inoc 
##       236.595985        89.830917
```

```
vif(Mod_Wf_13)
```

```
##                Wi          Ne_15_34          Ne_harmo       Wi:Ne_15_34 
##          37.53053         121.61945          90.61331         961.08349 
##       Wi:Ne_harmo Ne_15_34:Ne_harmo 
##         597.32545          82.84286
```

```
vif(Mod_Wf_14)
```

```
##               Wi         Ne_15_34          Ne_inoc      Wi:Ne_15_34 
##         8.656724         5.385342         8.418001        16.317311 
##       Wi:Ne_inoc Ne_15_34:Ne_inoc 
##        21.381575        10.626063
```

```
vif(Mod_Wf_15)
```

```
##               Wi         Ne_harmo          Ne_inoc      Wi:Ne_harmo 
##        18.894530         6.537792       160.885369        31.008953 
##       Wi:Ne_inoc Ne_harmo:Ne_inoc 
##        20.946993       182.033446
```

```
## Three Ne estimates

vif(Mod_Wf_19)
```

```
##          Ne_1_6         Ne_7_10         Ne_inoc  Ne_1_6:Ne_7_10  Ne_1_6:Ne_inoc 
##        2.177826       20.531067       46.374404       45.630292       56.458311 
## Ne_7_10:Ne_inoc 
##       16.546737
```

```
vif(Mod_Wf_22)
```

```
##           Ne_1_6         Ne_11_14          Ne_inoc  Ne_1_6:Ne_11_14 
##        282.22928         10.89279         48.02611        389.02603 
##   Ne_1_6:Ne_inoc Ne_11_14:Ne_inoc 
##       3776.65916       5102.69542
```

```
vif(Mod_Wf_24)
```

```
##           Ne_1_6         Ne_15_34          Ne_inoc  Ne_1_6:Ne_15_34 
##         19.58665         13.75167         91.00283         57.35546 
##   Ne_1_6:Ne_inoc Ne_15_34:Ne_inoc 
##         39.56230         34.45361
```

```
vif(Mod_Wf_25)
```

```
##           Ne_1_6         Ne_harmo          Ne_inoc  Ne_1_6:Ne_harmo 
##         5.512883        25.774124        48.878170        72.578018 
##   Ne_1_6:Ne_inoc Ne_harmo:Ne_inoc 
##        63.290094        59.293490
```

```
vif(Mod_Wf_28)
```

```
##          Ne_7_10         Ne_11_14          Ne_inoc Ne_7_10:Ne_11_14 
##         62.35563         67.21470         58.08944         67.77602 
##  Ne_7_10:Ne_inoc Ne_11_14:Ne_inoc 
##         45.52443        266.61798
```

```
vif(Mod_Wf_30)
```

```
##          Ne_7_10         Ne_15_34          Ne_inoc Ne_7_10:Ne_15_34 
##        111.49388         54.68275         83.19200        127.99548 
##  Ne_7_10:Ne_inoc Ne_15_34:Ne_inoc 
##         96.38532         87.19964
```

```
vif(Mod_Wf_31)
```

```
##          Ne_7_10         Ne_harmo          Ne_inoc Ne_7_10:Ne_harmo 
##        655.49243        169.72574         45.93162        729.59805 
##  Ne_7_10:Ne_inoc Ne_harmo:Ne_inoc 
##        239.22904        303.89171
```

```
vif(Mod_Wf_33)
```

```
##          Ne_11_14          Ne_15_34           Ne_inoc Ne_11_14:Ne_15_34 
##         291.06473          45.31664         154.86867          34.26211 
##  Ne_11_14:Ne_inoc  Ne_15_34:Ne_inoc 
##         575.51654          40.01854
```

```
vif(Mod_Wf_34)
```

```
##          Ne_11_14          Ne_harmo           Ne_inoc Ne_11_14:Ne_harmo 
##         138.67665          31.43675          56.57527          85.47093 
##  Ne_11_14:Ne_inoc  Ne_harmo:Ne_inoc 
##         153.23372          59.40206
```

```
vif(Mod_Wf_35)
```

```
##          Ne_15_34          Ne_harmo           Ne_inoc Ne_15_34:Ne_harmo 
##         1663.0365         9083.3013          267.9544         2433.1644 
##  Ne_15_34:Ne_inoc  Ne_harmo:Ne_inoc 
##         7193.6515        13614.3854
```

```
## For some models with only Ne explanatory variables, VIF cannot be calculated because the Ne are highly correlated. The following error appears: “there are aliased coefficients in the model.“

# vif(Mod_Wf_16)
# vif(Mod_Wf_17)
# vif(Mod_Wf_18)
# vif(Mod_Wf_20)
# vif(Mod_Wf_21)
# vif(Mod_Wf_23)
# vif(Mod_Wf_26)
# vif(Mod_Wf_27)
# vif(Mod_Wf_29)
# vif(Mod_Wf_32)
```

###### VIF 2 variables

**VIF:** If two explanatory variables are retained in
the model, the VIF values are all below 11 for all the models with Wi
and one Ne estimate. However, the VIF values are still high for most
models with two Ne estimates.

```
# Models with 2 explanatory variables
#-----------------------------------------------------

## Wi and one Ne estimate

Mod_Wf_Wi_Ne_1_6 <- glm(Wf ~ Wi*Ne_1_6, data=DataNoExtinction, family=gaussian)
Mod_Wf_Wi_Ne_7_10 <- glm(Wf ~ Wi*Ne_7_10, data=DataNoExtinction, family=gaussian)
Mod_Wf_Wi_Ne_11_14 <- glm(Wf ~ Wi*Ne_11_14, data=DataNoExtinction, family=gaussian)
Mod_Wf_Wi_Ne_15_34 <- glm(Wf ~ Wi*Ne_15_34, data=DataNoExtinction, family=gaussian)
Mod_Wf_Wi_Ne_harmo <- glm(Wf ~ Wi*Ne_harmo, data=DataNoExtinction, family=gaussian)
Mod_Wf_Wi_Ne_inoc <- glm(Wf ~ Wi*Ne_inoc, data=DataNoExtinction, family=gaussian)

## Two Ne estimates

Mod_Wf_Ne_1_6_Ne_7_10  <- glm(Wf ~ Ne_1_6*Ne_7_10, data=DataNoExtinction, family=gaussian)
Mod_Wf_Ne_1_6_Ne_11_14 <- glm(Wf ~ Ne_1_6*Ne_11_14, data=DataNoExtinction, family=gaussian)
Mod_Wf_Ne_1_6_Ne_15_34 <- glm(Wf ~ Ne_1_6*Ne_15_34, data=DataNoExtinction, family=gaussian)
Mod_Wf_Ne_1_6_Ne_harmo <- glm(Wf ~ Ne_1_6*Ne_harmo, data=DataNoExtinction, family=gaussian)
Mod_Wf_Ne_1_6_Ne_inoc <- glm(Wf ~ Ne_1_6*Ne_inoc, data=DataNoExtinction, family=gaussian)
Mod_Wf_Ne_7_10_Ne_11_14 <- glm(Wf ~ Ne_7_10*Ne_11_14, data=DataNoExtinction, family=gaussian)
Mod_Wf_Ne_7_10_Ne_15_34 <- glm(Wf ~ Ne_7_10*Ne_15_34, data=DataNoExtinction, family=gaussian)
Mod_Wf_Ne_7_10_Ne_harmo <- glm(Wf ~ Ne_7_10*Ne_harmo, data=DataNoExtinction, family=gaussian)
Mod_Wf_Ne_7_10_Ne_inoc <- glm(Wf ~ Ne_7_10*Ne_inoc, data=DataNoExtinction, family=gaussian)
Mod_Wf_Ne_11_14_Ne_15_34 <- glm(Wf ~ Ne_11_14*Ne_15_34, data=DataNoExtinction, family=gaussian)
Mod_Wf_Ne_11_14_Ne_harmo <- glm(Wf ~ Ne_11_14*Ne_harmo, data=DataNoExtinction, family=gaussian)
Mod_Wf_Ne_11_14_Ne_inoc <- glm(Wf ~ Ne_11_14*Ne_inoc, data=DataNoExtinction, family=gaussian)
Mod_Wf_Ne_15_34_Ne_harmo <- glm(Wf ~ Ne_15_34*Ne_harmo, data=DataNoExtinction, family=gaussian)
Mod_Wf_Ne_15_34_Ne_inoc <- glm(Wf ~ Ne_15_34*Ne_inoc, data=DataNoExtinction, family=gaussian)
Mod_Wf_Ne_harmo_Ne_inoc <- glm(Wf ~ Ne_harmo*Ne_inoc, data=DataNoExtinction, family=gaussian)
```

```
# VIF
#-----------------------------------------------------

## Wi and one Ne estimate

vif(Mod_Wf_Wi_Ne_1_6)
```

```
##        Wi    Ne_1_6 Wi:Ne_1_6 
##  2.400937  1.694926  1.800650
```

```
vif(Mod_Wf_Wi_Ne_7_10)
```

```
##         Wi    Ne_7_10 Wi:Ne_7_10 
##   3.539570   3.643027   6.779657
```

```
vif(Mod_Wf_Wi_Ne_11_14)
```

```
##          Wi    Ne_11_14 Wi:Ne_11_14 
##    5.586938    4.052625    5.054043
```

```
vif(Mod_Wf_Wi_Ne_15_34)
```

```
##          Wi    Ne_15_34 Wi:Ne_15_34 
##    6.397554    4.063236   10.366987
```

```
vif(Mod_Wf_Wi_Ne_harmo)
```

```
##          Wi    Ne_harmo Wi:Ne_harmo 
##    5.141378    3.950014    7.099874
```

```
vif(Mod_Wf_Wi_Ne_inoc)
```

```
##         Wi    Ne_inoc Wi:Ne_inoc 
##   5.521332   4.420703   9.266002
```

```
## Two Ne estimates

vif(Mod_Wf_Ne_1_6_Ne_7_10)
```

```
##         Ne_1_6        Ne_7_10 Ne_1_6:Ne_7_10 
##       1.338406       4.406722       4.962773
```

```
vif(Mod_Wf_Ne_1_6_Ne_11_14)
```

```
##          Ne_1_6        Ne_11_14 Ne_1_6:Ne_11_14 
##        4.224852        8.850310       16.076328
```

```
vif(Mod_Wf_Ne_1_6_Ne_15_34)
```

```
##          Ne_1_6        Ne_15_34 Ne_1_6:Ne_15_34 
##        11.14041         7.95200        20.22761
```

```
vif(Mod_Wf_Ne_1_6_Ne_harmo)
```

```
##          Ne_1_6        Ne_harmo Ne_1_6:Ne_harmo 
##        2.770304        4.226623        7.162953
```

```
vif(Mod_Wf_Ne_1_6_Ne_inoc)
```

```
##         Ne_1_6        Ne_inoc Ne_1_6:Ne_inoc 
##       1.752092       6.334755       7.816375
```

```
vif(Mod_Wf_Ne_7_10_Ne_11_14)
```

```
##          Ne_7_10         Ne_11_14 Ne_7_10:Ne_11_14 
##         4.128044         4.575958        10.045557
```

```
vif(Mod_Wf_Ne_7_10_Ne_15_34)
```

```
##          Ne_7_10         Ne_15_34 Ne_7_10:Ne_15_34 
##         5.629158         7.148413        15.575840
```

```
vif(Mod_Wf_Ne_7_10_Ne_harmo)
```

```
##          Ne_7_10         Ne_harmo Ne_7_10:Ne_harmo 
##        413.29759         11.96311        518.61690
```

```
vif(Mod_Wf_Ne_7_10_Ne_inoc)
```

```
##         Ne_7_10         Ne_inoc Ne_7_10:Ne_inoc 
##        2.605683        8.741011       12.701101
```

```
vif(Mod_Wf_Ne_11_14_Ne_15_34)
```

```
##          Ne_11_14          Ne_15_34 Ne_11_14:Ne_15_34 
##          6.427917          6.855876         15.856141
```

```
vif(Mod_Wf_Ne_11_14_Ne_harmo)
```

```
##          Ne_11_14          Ne_harmo Ne_11_14:Ne_harmo 
##         62.661302          5.047132         83.048980
```

```
vif(Mod_Wf_Ne_11_14_Ne_inoc)
```

```
##         Ne_11_14          Ne_inoc Ne_11_14:Ne_inoc 
##         7.729139         6.690494        18.949822
```

```
vif(Mod_Wf_Ne_15_34_Ne_harmo)
```

```
##          Ne_15_34          Ne_harmo Ne_15_34:Ne_harmo 
##         46.856026          6.994341         67.839494
```

```
vif(Mod_Wf_Ne_15_34_Ne_inoc)
```

```
##         Ne_15_34          Ne_inoc Ne_15_34:Ne_inoc 
##         2.175085         5.727682         7.989201
```

```
vif(Mod_Wf_Ne_harmo_Ne_inoc)
```

```
##         Ne_harmo          Ne_inoc Ne_harmo:Ne_inoc 
##         3.676032        39.528203        51.547575
```

###### AIC

**AIC:** Model selection based on Akaike Information
Criterion (AIC). The model including Wi and Ne estimated from 7 to 10
days post inoculation is the one with the lowest AIC
(**47.94**).

```
## Wi and one Ne estimate

Mod_Wf_Wi_Ne_1_6
```

```
## 
## Call:  glm(formula = Wf ~ Wi * Ne_1_6, family = gaussian, data = DataNoExtinction)
## 
## Coefficients:
## (Intercept)           Wi       Ne_1_6    Wi:Ne_1_6  
##   0.4234421    0.4784932    0.0001573    0.0005652  
## 
## Degrees of Freedom: 54 Total (i.e. Null);  51 Residual
## Null Deviance:       11.01 
## Residual Deviance: 8.144     AIC: 61.03
```

```
Mod_Wf_Wi_Ne_7_10
```

```
## 
## Call:  glm(formula = Wf ~ Wi * Ne_7_10, family = gaussian, data = DataNoExtinction)
## 
## Coefficients:
## (Intercept)           Wi      Ne_7_10   Wi:Ne_7_10  
##   -0.019578     1.033098     0.001949    -0.001913  
## 
## Degrees of Freedom: 54 Total (i.e. Null);  51 Residual
## Null Deviance:       11.01 
## Residual Deviance: 6.419     AIC: 47.94
```

```
Mod_Wf_Wi_Ne_11_14
```

```
## 
## Call:  glm(formula = Wf ~ Wi * Ne_11_14, family = gaussian, data = DataNoExtinction)
## 
## Coefficients:
## (Intercept)           Wi     Ne_11_14  Wi:Ne_11_14  
##   0.2178801    0.6756476    0.0009228   -0.0001892  
## 
## Degrees of Freedom: 54 Total (i.e. Null);  51 Residual
## Null Deviance:       11.01 
## Residual Deviance: 7.799     AIC: 58.65
```

```
Mod_Wf_Wi_Ne_15_34
```

```
## 
## Call:  glm(formula = Wf ~ Wi * Ne_15_34, family = gaussian, data = DataNoExtinction)
## 
## Coefficients:
## (Intercept)           Wi     Ne_15_34  Wi:Ne_15_34  
##   0.4352815    0.7034400    0.0001030   -0.0002328  
## 
## Degrees of Freedom: 54 Total (i.e. Null);  51 Residual
## Null Deviance:       11.01 
## Residual Deviance: 8.36  AIC: 62.47
```

```
Mod_Wf_Wi_Ne_harmo
```

```
## 
## Call:  glm(formula = Wf ~ Wi * Ne_harmo, family = gaussian, data = DataNoExtinction)
## 
## Coefficients:
## (Intercept)           Wi     Ne_harmo  Wi:Ne_harmo  
##   0.1844148    0.8477070    0.0008350   -0.0008393  
## 
## Degrees of Freedom: 54 Total (i.e. Null);  51 Residual
## Null Deviance:       11.01 
## Residual Deviance: 8.069     AIC: 60.52
```

```
Mod_Wf_Wi_Ne_inoc
```

```
## 
## Call:  glm(formula = Wf ~ Wi * Ne_inoc, family = gaussian, data = DataNoExtinction)
## 
## Coefficients:
## (Intercept)           Wi      Ne_inoc   Wi:Ne_inoc  
##     0.40058      0.83140      0.03024     -0.07444  
## 
## Degrees of Freedom: 54 Total (i.e. Null);  51 Residual
## Null Deviance:       11.01 
## Residual Deviance: 8.207     AIC: 61.45
```

```
## Two Ne estimates

Mod_Wf_Ne_1_6_Ne_7_10
```

```
## 
## Call:  glm(formula = Wf ~ Ne_1_6 * Ne_7_10, family = gaussian, data = DataNoExtinction)
## 
## Coefficients:
##    (Intercept)          Ne_1_6         Ne_7_10  Ne_1_6:Ne_7_10  
##      7.092e-01      -4.579e-04       1.344e-04       2.360e-06  
## 
## Degrees of Freedom: 54 Total (i.e. Null);  51 Residual
## Null Deviance:       11.01 
## Residual Deviance: 9.231     AIC: 67.92
```

```
Mod_Wf_Ne_1_6_Ne_11_14
```

```
## 
## Call:  glm(formula = Wf ~ Ne_1_6 * Ne_11_14, family = gaussian, data = DataNoExtinction)
## 
## Coefficients:
##     (Intercept)           Ne_1_6         Ne_11_14  Ne_1_6:Ne_11_14  
##       8.380e-01       -2.249e-04        5.147e-04       -6.417e-07  
## 
## Degrees of Freedom: 54 Total (i.e. Null);  51 Residual
## Null Deviance:       11.01 
## Residual Deviance: 10.75     AIC: 76.29
```

```
Mod_Wf_Ne_1_6_Ne_15_34
```

```
## 
## Call:  glm(formula = Wf ~ Ne_1_6 * Ne_15_34, family = gaussian, data = DataNoExtinction)
## 
## Coefficients:
##     (Intercept)           Ne_1_6         Ne_15_34  Ne_1_6:Ne_15_34  
##       4.912e-01        3.190e-03        1.912e-03       -8.793e-06  
## 
## Degrees of Freedom: 54 Total (i.e. Null);  51 Residual
## Null Deviance:       11.01 
## Residual Deviance: 6.678     AIC: 50.11
```

```
Mod_Wf_Ne_1_6_Ne_harmo
```

```
## 
## Call:  glm(formula = Wf ~ Ne_1_6 * Ne_harmo, family = gaussian, data = DataNoExtinction)
## 
## Coefficients:
##     (Intercept)           Ne_1_6         Ne_harmo  Ne_1_6:Ne_harmo  
##       8.206e-01       -7.564e-05        5.524e-04       -1.340e-06  
## 
## Degrees of Freedom: 54 Total (i.e. Null);  51 Residual
## Null Deviance:       11.01 
## Residual Deviance: 10.66     AIC: 75.85
```

```
Mod_Wf_Ne_1_6_Ne_inoc
```

```
## 
## Call:  glm(formula = Wf ~ Ne_1_6 * Ne_inoc, family = gaussian, data = DataNoExtinction)
## 
## Coefficients:
##    (Intercept)          Ne_1_6         Ne_inoc  Ne_1_6:Ne_inoc  
##      9.109e-01      -2.549e-04      -4.054e-03       1.451e-05  
## 
## Degrees of Freedom: 54 Total (i.e. Null);  51 Residual
## Null Deviance:       11.01 
## Residual Deviance: 10.82     AIC: 76.66
```

```
Mod_Wf_Ne_7_10_Ne_11_14
```

```
## 
## Call:  glm(formula = Wf ~ Ne_7_10 * Ne_11_14, family = gaussian, data = DataNoExtinction)
## 
## Coefficients:
##      (Intercept)           Ne_7_10          Ne_11_14  Ne_7_10:Ne_11_14  
##        9.681e-01        -6.511e-04        -3.202e-03         9.511e-06  
## 
## Degrees of Freedom: 54 Total (i.e. Null);  51 Residual
## Null Deviance:       11.01 
## Residual Deviance: 7.161     AIC: 53.95
```

```
Mod_Wf_Ne_7_10_Ne_15_34
```

```
## 
## Call:  glm(formula = Wf ~ Ne_7_10 * Ne_15_34, family = gaussian, data = DataNoExtinction)
## 
## Coefficients:
##      (Intercept)           Ne_7_10          Ne_15_34  Ne_7_10:Ne_15_34  
##        8.575e-01         1.168e-03        -8.561e-04         9.153e-07  
## 
## Degrees of Freedom: 54 Total (i.e. Null);  51 Residual
## Null Deviance:       11.01 
## Residual Deviance: 8.449     AIC: 63.06
```

```
Mod_Wf_Ne_7_10_Ne_harmo
```

```
## 
## Call:  glm(formula = Wf ~ Ne_7_10 * Ne_harmo, family = gaussian, data = DataNoExtinction)
## 
## Coefficients:
##      (Intercept)           Ne_7_10          Ne_harmo  Ne_7_10:Ne_harmo  
##        4.375e-01         1.052e-02        -8.408e-04        -1.732e-05  
## 
## Degrees of Freedom: 54 Total (i.e. Null);  51 Residual
## Null Deviance:       11.01 
## Residual Deviance: 7.318     AIC: 55.15
```

```
Mod_Wf_Ne_7_10_Ne_inoc
```

```
## 
## Call:  glm(formula = Wf ~ Ne_7_10 * Ne_inoc, family = gaussian, data = DataNoExtinction)
## 
## Coefficients:
##     (Intercept)          Ne_7_10          Ne_inoc  Ne_7_10:Ne_inoc  
##        0.769887         0.000690        -0.071815         0.000114  
## 
## Degrees of Freedom: 54 Total (i.e. Null);  51 Residual
## Null Deviance:       11.01 
## Residual Deviance: 9.286     AIC: 68.25
```

```
Mod_Wf_Ne_11_14_Ne_15_34
```

```
## 
## Call:  glm(formula = Wf ~ Ne_11_14 * Ne_15_34, family = gaussian, data = DataNoExtinction)
## 
## Coefficients:
##       (Intercept)           Ne_11_14           Ne_15_34  Ne_11_14:Ne_15_34  
##         3.011e-01          4.318e-03          1.711e-03         -8.938e-06  
## 
## Degrees of Freedom: 54 Total (i.e. Null);  51 Residual
## Null Deviance:       11.01 
## Residual Deviance: 6.91  AIC: 51.99
```

```
Mod_Wf_Ne_11_14_Ne_harmo
```

```
## 
## Call:  glm(formula = Wf ~ Ne_11_14 * Ne_harmo, family = gaussian, data = DataNoExtinction)
## 
## Coefficients:
##       (Intercept)           Ne_11_14           Ne_harmo  Ne_11_14:Ne_harmo  
##         4.902e-01          5.565e-03          1.110e-03         -1.258e-05  
## 
## Degrees of Freedom: 54 Total (i.e. Null);  51 Residual
## Null Deviance:       11.01 
## Residual Deviance: 10.32     AIC: 74.06
```

```
Mod_Wf_Ne_11_14_Ne_inoc
```

```
## 
## Call:  glm(formula = Wf ~ Ne_11_14 * Ne_inoc, family = gaussian, data = DataNoExtinction)
## 
## Coefficients:
##      (Intercept)          Ne_11_14           Ne_inoc  Ne_11_14:Ne_inoc  
##        7.973e-01         3.883e-04         6.151e-03        -5.426e-05  
## 
## Degrees of Freedom: 54 Total (i.e. Null);  51 Residual
## Null Deviance:       11.01 
## Residual Deviance: 10.98     AIC: 77.48
```

```
Mod_Wf_Ne_15_34_Ne_harmo
```

```
## 
## Call:  glm(formula = Wf ~ Ne_15_34 * Ne_harmo, family = gaussian, data = DataNoExtinction)
## 
## Coefficients:
##       (Intercept)           Ne_15_34           Ne_harmo  Ne_15_34:Ne_harmo  
##         1.180e-01          3.114e-03          2.017e-03         -6.859e-06  
## 
## Degrees of Freedom: 54 Total (i.e. Null);  51 Residual
## Null Deviance:       11.01 
## Residual Deviance: 9.012     AIC: 66.6
```

```
Mod_Wf_Ne_15_34_Ne_inoc
```

```
## 
## Call:  glm(formula = Wf ~ Ne_15_34 * Ne_inoc, family = gaussian, data = DataNoExtinction)
## 
## Coefficients:
##      (Intercept)          Ne_15_34           Ne_inoc  Ne_15_34:Ne_inoc  
##        0.4188214         0.0006482         0.1477169        -0.0001965  
## 
## Degrees of Freedom: 54 Total (i.e. Null);  51 Residual
## Null Deviance:       11.01 
## Residual Deviance: 8.069     AIC: 60.52
```

```
Mod_Wf_Ne_harmo_Ne_inoc
```

```
## 
## Call:  glm(formula = Wf ~ Ne_harmo * Ne_inoc, family = gaussian, data = DataNoExtinction)
## 
## Coefficients:
##      (Intercept)          Ne_harmo           Ne_inoc  Ne_harmo:Ne_inoc  
##        -0.069539          0.002316          0.519360         -0.001135  
## 
## Degrees of Freedom: 54 Total (i.e. Null);  51 Residual
## Null Deviance:       11.01 
## Residual Deviance: 6.598     AIC: 49.45
```

###### Model fit

**Model Fit:** Among the models that retain two
explanatory variables, the one with the highest R-squared is the one
with Wi and Ne estimated from 7 to 10 days post inoculation
(**R-squared = 0.4169404**). Moreover, the Chi-squared
goodness of fit test indicates that the Gaussian function is a good fit
to the observed data (p=1).

```
# McFadden's R-squared
#-----------------------------------------------------

## Wi and one Ne estimate

pR2(Mod_Wf_Wi_Ne_1_6)
```

```
## fitting null model for pseudo-r2
```

```
##         llh     llhNull          G2    McFadden        r2ML        r2CU 
## -25.5151311 -33.8062826  16.5823030   0.2452548   0.2602895   0.3678960
```

```
pR2(Mod_Wf_Wi_Ne_7_10)
```

```
## fitting null model for pseudo-r2
```

```
##         llh     llhNull          G2    McFadden        r2ML        r2CU 
## -18.9709727 -33.8062826  29.6706198   0.4388329   0.4169404   0.5893080
```

```
pR2(Mod_Wf_Wi_Ne_11_14)
```

```
## fitting null model for pseudo-r2
```

```
##         llh     llhNull          G2    McFadden        r2ML        r2CU 
## -24.3246620 -33.8062826  18.9632412   0.2804692   0.2916282   0.4121905
```

```
pR2(Mod_Wf_Wi_Ne_15_34)
```

```
## fitting null model for pseudo-r2
```

```
##         llh     llhNull          G2    McFadden        r2ML        r2CU 
## -26.2358125 -33.8062826  15.1409403   0.2239368   0.2406480   0.3401345
```

```
pR2(Mod_Wf_Wi_Ne_harmo)
```

```
## fitting null model for pseudo-r2
```

```
##         llh     llhNull          G2    McFadden        r2ML        r2CU 
## -25.2601576 -33.8062826  17.0922501   0.2527969   0.2671162   0.3775450
```

```
pR2(Mod_Wf_Wi_Ne_inoc)
```

```
## fitting null model for pseudo-r2
```

```
##         llh     llhNull          G2    McFadden        r2ML        r2CU 
## -25.7258064 -33.8062826  16.1609524   0.2390229   0.2546009   0.3598557
```

```
## Two Ne estimates

pR2(Mod_Wf_Ne_1_6_Ne_7_10)
```

```
## fitting null model for pseudo-r2
```

```
##         llh     llhNull          G2    McFadden        r2ML        r2CU 
## -28.9595600 -33.8062826   9.6934453   0.1433675   0.1615870   0.2283888
```

```
pR2(Mod_Wf_Ne_1_6_Ne_11_14)
```

```
## fitting null model for pseudo-r2
```

```
##          llh      llhNull           G2     McFadden         r2ML         r2CU 
## -33.14404381 -33.80628262   1.32447764   0.01958922   0.02379377   0.03363037
```

```
pR2(Mod_Wf_Ne_1_6_Ne_15_34)
```

```
## fitting null model for pseudo-r2
```

```
##         llh     llhNull          G2    McFadden        r2ML        r2CU 
## -20.0567877 -33.8062826  27.4989899   0.4067142   0.3934582   0.5561181
```

```
pR2(Mod_Wf_Ne_1_6_Ne_harmo)
```

```
## fitting null model for pseudo-r2
```

```
##          llh      llhNull           G2     McFadden         r2ML         r2CU 
## -32.92508047 -33.80628262   1.76240431   0.02606622   0.03153575   0.04457298
```

```
pR2(Mod_Wf_Ne_1_6_Ne_inoc)
```

```
## fitting null model for pseudo-r2
```

```
##          llh      llhNull           G2     McFadden         r2ML         r2CU 
## -33.33023613 -33.80628262   0.95209299   0.01408160   0.01716181   0.02425669
```

```
pR2(Mod_Wf_Ne_7_10_Ne_11_14)
```

```
## fitting null model for pseudo-r2
```

```
##         llh     llhNull          G2    McFadden        r2ML        r2CU 
## -21.9766046 -33.8062826  23.6593560   0.3499254   0.3496016   0.4941306
```

```
pR2(Mod_Wf_Ne_7_10_Ne_15_34)
```

```
## fitting null model for pseudo-r2
```

```
##         llh     llhNull          G2    McFadden        r2ML        r2CU 
## -26.5277980 -33.8062826  14.5569693   0.2152998   0.2325425   0.3286781
```

```
pR2(Mod_Wf_Ne_7_10_Ne_harmo)
```

```
## fitting null model for pseudo-r2
```

```
##         llh     llhNull          G2    McFadden        r2ML        r2CU 
## -22.5749217 -33.8062826  22.4627218   0.3322270   0.3352958   0.4739107
```

```
pR2(Mod_Wf_Ne_7_10_Ne_inoc)
```

```
## fitting null model for pseudo-r2
```

```
##         llh     llhNull          G2    McFadden        r2ML        r2CU 
## -29.1243678 -33.8062826   9.3638296   0.1384924   0.1565473   0.2212656
```

```
pR2(Mod_Wf_Ne_11_14_Ne_15_34)
```

```
## fitting null model for pseudo-r2
```

```
##         llh     llhNull          G2    McFadden        r2ML        r2CU 
## -20.9964081 -33.8062826  25.6197490   0.3789199   0.3723757   0.5263199
```

```
pR2(Mod_Wf_Ne_11_14_Ne_harmo)
```

```
## fitting null model for pseudo-r2
```

```
##          llh      llhNull           G2     McFadden         r2ML         r2CU 
## -32.03222268 -33.80628262   3.54811988   0.05247723   0.06247445   0.08830206
```

```
pR2(Mod_Wf_Ne_11_14_Ne_inoc)
```

```
## fitting null model for pseudo-r2
```

```
##           llh       llhNull            G2      McFadden          r2ML 
## -33.738411948 -33.806282625   0.135741354   0.002007635   0.002464982 
##          r2CU 
##   0.003484031
```

```
pR2(Mod_Wf_Ne_15_34_Ne_harmo)
```

```
## fitting null model for pseudo-r2
```

```
##         llh     llhNull          G2    McFadden        r2ML        r2CU 
## -28.2995199 -33.8062826  11.0135255   0.1628917   0.1814706   0.2564924
```

```
pR2(Mod_Wf_Ne_15_34_Ne_inoc)
```

```
## fitting null model for pseudo-r2
```

```
##         llh     llhNull          G2    McFadden        r2ML        r2CU 
## -25.2621085 -33.8062826  17.0883483   0.2527392   0.2670643   0.3774715
```

```
pR2(Mod_Wf_Ne_harmo_Ne_inoc)
```

```
## fitting null model for pseudo-r2
```

```
##         llh     llhNull          G2    McFadden        r2ML        r2CU 
## -19.7266899 -33.8062826  28.1591855   0.4164786   0.4006953   0.5663471
```

```
# Chi-squared test 
#-----------------------------------------------------

Mod_Wf_Wi_Ne_7_10
```

```
## 
## Call:  glm(formula = Wf ~ Wi * Ne_7_10, family = gaussian, data = DataNoExtinction)
## 
## Coefficients:
## (Intercept)           Wi      Ne_7_10   Wi:Ne_7_10  
##   -0.019578     1.033098     0.001949    -0.001913  
## 
## Degrees of Freedom: 54 Total (i.e. Null);  51 Residual
## Null Deviance:       11.01 
## Residual Deviance: 6.419     AIC: 47.94
```

```
LLR = -2 * ( 11.01 - 6.419)
pchisq(LLR, 2, lower.tail = FALSE)
```

```
## [1] 1
```

###### Model selection

**Model selection:** The model with the lowest AIC is
the full model (Wi, Ne\_7\_10 and Wi\*Ne\_7\_10).

```
options(na.action = "na.fail")
dredge(Mod_Wf_Wi_Ne_7_10)
```

```
## Global model call: glm(formula = Wf ~ Wi * Ne_7_10, family = gaussian, data = DataNoExtinction)
## ---
## Model selection table 
##      (Int)   Ne_7_10     Wi Ne_7_10:Wi df  logLik AICc delta weight
## 8 -0.01958 0.0019490 1.0330  -0.001913  5 -18.971 49.2  0.00  0.967
## 4  0.34070 0.0006566 0.4749             4 -23.741 56.3  7.12  0.028
## 3  0.52050           0.5101             3 -26.520 59.5 10.34  0.005
## 2  0.59850 0.0007878                    3 -30.671 67.8 18.65  0.000
## 1  0.84070                              2 -33.806 71.8 22.68  0.000
## Models ranked by AICc(x)
```

```
summary(Mod_Wf_Wi_Ne_7_10)
```

```
## 
## Call:
## glm(formula = Wf ~ Wi * Ne_7_10, family = gaussian, data = DataNoExtinction)
## 
## Deviance Residuals: 
##      Min        1Q    Median        3Q       Max  
## -0.71222  -0.20800  -0.02558   0.16120   0.78876  
## 
## Coefficients:
##               Estimate Std. Error t value Pr(>|t|)    
## (Intercept) -0.0195784  0.1605441  -0.122 0.903417    
## Wi           1.0330979  0.2126802   4.858 1.17e-05 ***
## Ne_7_10      0.0019494  0.0004898   3.980 0.000218 ***
## Wi:Ne_7_10  -0.0019132  0.0006156  -3.108 0.003078 ** 
## ---
## Signif. codes:  0 '***' 0.001 '**' 0.01 '*' 0.05 '.' 0.1 ' ' 1
## 
## (Dispersion parameter for gaussian family taken to be 0.1258687)
## 
##     Null deviance: 11.0097  on 54  degrees of freedom
## Residual deviance:  6.4193  on 51  degrees of freedom
## AIC: 47.942
## 
## Number of Fisher Scoring iterations: 2
```

**Residuals distribution:** Residuals have a Gaussian
distribution (Shapiro test, p=0.454).

```
hist(residuals(Mod_Wf_Wi_Ne_7_10), col="grey", breaks=6, main="Residuals distribution", xlab="")
```

```
ggqqplot(residuals(Mod_Wf_Wi_Ne_7_10))
```

```
shapiro.test(residuals(Mod_Wf_Wi_Ne_7_10))
```

```
## 
##  Shapiro-Wilk normality test
## 
## data:  residuals(Mod_Wf_Wi_Ne_7_10)
## W = 0.97919, p-value = 0.454
```

###### Effect plot

```
visreg(Mod_Wf_Wi_Ne_7_10, "Wi", by="Ne_7_10", breaks=c(50,500),strip.names=c("Ne=50","Ne=500"),
       points=list(cex=1,pch=19),ylab="Final replicative fitness (Wf)",xlab="Replicative fitness of the initially inoculated virus (Wi)",scale = "response",rug=2,jitter=TRUE)
```

###### Other response variables: Wf/Wi and Wf - Wi

Same models with the replicative fitness ratio Wf/Wi and the
replicative fitness difference Wf - Wi as response variables. The
results are similar to the model with Wf as the response variable.

```
# New models
#----------------------------------------------------

V2 <- DataNoExtinction$Wf/DataNoExtinction$Wi
V2[is.infinite(V2)] <- NA
V3 <- DataNoExtinction$Wf-DataNoExtinction$Wi
DataNoExtinction<-cbind(DataNoExtinction,Wf_Div_Wi=V2,Wf_Minus_Wi=V3)

Mod_Wf_Div_Wi_Wi_Ne_7_10 <- glm(Wf_Div_Wi ~ Wi*Ne_7_10, data=DataNoExtinction, family=gaussian, na.action=na.exclude)
Mod_Wf_Minus_Wi_Wi_Ne_7_10 <- glm(Wf_Minus_Wi ~ Wi*Ne_7_10, data=DataNoExtinction, family=gaussian)


# Effect plot replicative fitness ratio Wf/Wi
#----------------------------------------------------

visreg(Mod_Wf_Div_Wi_Wi_Ne_7_10, "Wi", by="Ne_7_10", breaks=c(50,500),strip.names=c("Ne=50","Ne=500"),
       points=list(cex=1,pch=19),ylab="Replicative fitness ratio Wf/Wi",xlab="Replicative fitness of the initially inoculated virus (Wi)",scale = "response",rug=2,jitter=TRUE)
```

```
# Effect plot replicative fitness difference Wf - Wi
#----------------------------------------------------

visreg(Mod_Wf_Minus_Wi_Wi_Ne_7_10, "Wi", by="Ne_7_10", breaks=c(50,500),strip.names=c("Ne=50","Ne=500"),
       points=list(cex=1,pch=19),ylab="Replicative fitness difference Wf - Wi",xlab="Replicative fitness of the initially inoculated virus (Wi)",scale = "response",rug=2,jitter=TRUE)
```

### **2. Figures 5A and 5B**

#### **Figure 5A**

Probability of fitness gain:

```
NameNe<-c(expression(paste(italic(N[e]),"=50")),expression(paste(italic(N[e]),"=500")))
XlabNameFitness<-"Replicative fitness of the initially inoculated virus (Wi)"

visreg(Mod_GainFit_Wi_Ne_7_10, "Wi", by="Ne_7_10", breaks=c(50,500),
                           ylab="Probability of fitness gain",xlab=XlabNameFitness,
                           scale = "response",rug=2,jitter=TRUE,overlay=TRUE,gg=TRUE,legend=FALSE) +
                           ggtitle("") +
                           theme(legend.position=c(0.85,0.85),axis.text=element_text(size=12),axis.title=element_text(size=12),
                           legend.text = element_text(size=12),legend.title = element_text(size=14),
                           panel.grid.major = element_blank(), panel.grid.minor = element_blank(),panel.background = element_blank(), axis.line = element_line(colour = "black"))
```

#### **Figure 5B**

Final replicative fitness:

```
NameNe<-c(expression(paste(italic(N[e]),"=50")),expression(paste(italic(N[e]),"=500")))
XlabNameFitness<-"Replicative fitness of the initially inoculated virus (Wi)"

visreg(Mod_Wf_Wi_Ne_7_10, "Wi", by="Ne_7_10", breaks=c(50,500),
                           ylab="Final replicative fitness (Wf)",xlab=XlabNameFitness,
                           scale = "response",rug=2,jitter=TRUE,overlay=TRUE,gg=TRUE,legend=FALSE) +
                           ggtitle("") +
                           theme(legend.position=c(0.85,0.85),axis.text=element_text(size=12),axis.title=element_text(size=12),
                           legend.text = element_text(size=12),legend.title = element_text(size=14),
                           panel.grid.major = element_blank(), panel.grid.minor = element_blank(),panel.background = element_blank(), axis.line = element_line(colour = "black"))
```

### **Figure S1:** Virulence of the final PVY populations that evolved on six pepper doubled-haploid (DH) lines

The sub-sections of Figure S1 and the associated statistical analyses
are presented bellow.

#### **Figure S1A: All pepper lines (plant height)**

##### Figure S1A

```
Data_Virulence_A$Time = factor(Data_Virulence_A$Time, levels=c("Mock", "Initial","Final"))
Data_Virulence_A$PlantGeno = factor(Data_Virulence_A$PlantGeno, levels=c("HD2256","HD219","HD2321","HD2349","HD2344","HD2173_119N"))

ggplot(data = Data_Virulence_A, aes(x=Time, y=Height)) + 
  geom_boxplot(aes(fill=Time)) +
  facet_wrap( ~ PlantGeno, scales="free") +
  ylim(0,40) +
  ylab("Plant height (cm)") +
  xlab("") +
  theme_bw(base_size = 15)+
  theme(axis.text.x=element_blank()) +
  scale_fill_manual(values = c("#999999", "#E69F00", "#56B4E9"), name = "Lignage", labels = c("Mock", "Initial PVY population", "Final PVY population"))
```

```
# ggsave("./FigureS1A.svg")
```

##### Statistics

A **Kruskal-Wallis** test has been used to compare the
mean height of the mock-inoculate plants, the plants inoculated with the
initial PVY population and the plants inoculated with final PVY
populations. If the Krukal-Wallis test was significant, a
**Nemenyi’s** non-parametric all-pairs comparison test has
been performed.

**HD2256**

```
Data_virulence_HD2256 <- filter(Data_Virulence_A, PlantGeno=="HD2256")

kruskal.test(Height ~ Time, data=Data_virulence_HD2256)
```

```
## 
##  Kruskal-Wallis rank sum test
## 
## data:  Height by Time
## Kruskal-Wallis chi-squared = 8.0582, df = 2, p-value = 0.01779
```

```
kwAllPairsNemenyiTest(Height ~ Time, data=Data_virulence_HD2256)
```

```
##         Mock  Initial
## Initial 0.013 -      
## Final   0.063 0.288
```

**HD219**

```
Data_virulence_HD219 <- filter(Data_Virulence_A, PlantGeno=="HD219")

kruskal.test(Height ~ Time, data=Data_virulence_HD219)
```

```
## 
##  Kruskal-Wallis rank sum test
## 
## data:  Height by Time
## Kruskal-Wallis chi-squared = 11.834, df = 2, p-value = 0.002694
```

```
kwAllPairsNemenyiTest(Height ~ Time, data=Data_virulence_HD219)
```

```
##         Mock   Initial
## Initial 0.0018 -      
## Final   0.0137 0.1748
```

**HD2321**

```
Data_virulence_HD2321 <- filter(Data_Virulence_A, PlantGeno=="HD2321")

kruskal.test(Height ~ Time, data=Data_virulence_HD2321)
```

```
## 
##  Kruskal-Wallis rank sum test
## 
## data:  Height by Time
## Kruskal-Wallis chi-squared = 3.2868, df = 1, p-value = 0.06984
```

**HD2349**

```
Data_virulence_HD2349 <- filter(Data_Virulence_A, PlantGeno=="HD2349")

kruskal.test(Height ~ Time, data=Data_virulence_HD2349)
```

```
## 
##  Kruskal-Wallis rank sum test
## 
## data:  Height by Time
## Kruskal-Wallis chi-squared = 0.24364, df = 2, p-value = 0.8853
```

**HD2344**

```
Data_virulence_HD2344 <- filter(Data_Virulence_A, PlantGeno=="HD2344")

kruskal.test(Height ~ Time, data=Data_virulence_HD2344)
```

```
## 
##  Kruskal-Wallis rank sum test
## 
## data:  Height by Time
## Kruskal-Wallis chi-squared = 1.9907, df = 2, p-value = 0.3696
```

**HD2173**

```
Data_virulence_HD2173 <- filter(Data_Virulence_A, PlantGeno=="HD2173_119N")

kruskal.test(Height ~ Time, data=Data_virulence_HD2173)
```

```
## 
##  Kruskal-Wallis rank sum test
## 
## data:  Height by Time
## Kruskal-Wallis chi-squared = 6.2023, df = 2, p-value = 0.045
```

```
kwAllPairsNemenyiTest(Height ~ Time, data=Data_virulence_HD2173)
```

```
##         Mock  Initial
## Initial 0.039 -      
## Final   0.054 0.722
```

#### **Figure S1B: All pepper lines (plant weight)**

##### Figure S1B

```
Data_Virulence_A$Time = factor(Data_Virulence_A$Time, levels=c("Mock", "Initial","Final"))
Data_Virulence_A$PlantGeno = factor(Data_Virulence_A$PlantGeno, levels=c("HD2256","HD219","HD2321","HD2349","HD2344","HD2173_119N"))

ggplot(data = Data_Virulence_A, aes(x=Time, y=Weight)) + 
  geom_boxplot(aes(fill=Time)) +
  facet_wrap( ~ PlantGeno, scales="free") +
  ylim(0,30) +
  ylab("Plant weight (g)") +
  xlab("") +
  theme_bw(base_size = 15)+
  theme(axis.text.x=element_blank()) +
  scale_fill_manual(values = c("#999999", "#E69F00", "#56B4E9"), name = "Lignage", labels = c("Mock", "Initial PVY population", "Final PVY population"))
```

```
# ggsave("./FigureS1B.svg")
```

##### Statistics

A **Kruskal-Wallis** test has been used to compare the
mean weight of the mock-inoculate plants, the plants inoculated with the
initial PVY population and the plants inoculated with final PVY
populations. If the Krukal-Wallis test was significant, a
**Nemenyi’s** non-parametric all-pairs comparison test has
been performed.

**HD2256**

```
Data_virulence_HD2256 <- filter(Data_Virulence_A, PlantGeno=="HD2256")

kruskal.test(Weight ~ Time, data=Data_virulence_HD2256)
```

```
## 
##  Kruskal-Wallis rank sum test
## 
## data:  Weight by Time
## Kruskal-Wallis chi-squared = 2.7475, df = 2, p-value = 0.2532
```

**HD219**

```
Data_virulence_HD219 <- filter(Data_Virulence_A, PlantGeno=="HD219")

kruskal.test(Weight ~ Time, data=Data_virulence_HD219)
```

```
## 
##  Kruskal-Wallis rank sum test
## 
## data:  Weight by Time
## Kruskal-Wallis chi-squared = 6.3352, df = 2, p-value = 0.0421
```

```
kwAllPairsNemenyiTest(Weight ~ Time, data=Data_virulence_HD219)
```

```
##         Mock  Initial
## Initial 0.154 -      
## Final   0.769 0.047
```

**HD2321**

```
Data_virulence_HD2321 <- filter(Data_Virulence_A, PlantGeno=="HD2321")

kruskal.test(Weight ~ Time, data=Data_virulence_HD2321)
```

```
## 
##  Kruskal-Wallis rank sum test
## 
## data:  Weight by Time
## Kruskal-Wallis chi-squared = 0.81768, df = 1, p-value = 0.3659
```

**HD2349**

```
Data_virulence_HD2349 <- filter(Data_Virulence_A, PlantGeno=="HD2349")

kruskal.test(Weight ~ Time, data=Data_virulence_HD2349)
```

```
## 
##  Kruskal-Wallis rank sum test
## 
## data:  Weight by Time
## Kruskal-Wallis chi-squared = 1.2649, df = 2, p-value = 0.5313
```

**HD2344**

```
Data_virulence_HD2344 <- filter(Data_Virulence_A, PlantGeno=="HD2344")

kruskal.test(Weight ~ Time, data=Data_virulence_HD2344)
```

```
## 
##  Kruskal-Wallis rank sum test
## 
## data:  Weight by Time
## Kruskal-Wallis chi-squared = 5.8212, df = 2, p-value = 0.05444
```

```
kwAllPairsNemenyiTest(Weight ~ Time, data=Data_virulence_HD2344)
```

```
##         Mock  Initial
## Initial 0.136 -      
## Final   0.044 0.987
```

**HD2173**

```
Data_virulence_HD2173 <- filter(Data_Virulence_A, PlantGeno=="HD2173_119N")

kruskal.test(Weight ~ Time, data=Data_virulence_HD2173)
```

```
## 
##  Kruskal-Wallis rank sum test
## 
## data:  Weight by Time
## Kruskal-Wallis chi-squared = 7.0787, df = 2, p-value = 0.02903
```

```
kwAllPairsNemenyiTest(Weight ~ Time, data=Data_virulence_HD2173)
```

```
##         Mock  Initial
## Initial 0.023 -      
## Final   0.114 0.235
```

#### **Figure S1C: HD2173 and the 3 PVY variants (Plant height)**

##### Figure S1c (Plant height)

```
Data_Virulence_B$Time = factor(Data_Virulence_B$Time, levels=c("Mock", "Initial","Final"))
Data_Virulence_B$PlantGeno = factor(Data_Virulence_B$PlantGeno, levels=c("HD2173_119N", "HD2173_101G", "HD2173_115K"))

ggplot(data = Data_Virulence_B, aes(x=Time, y=Height)) + 
  geom_boxplot(aes(fill=Time)) +
  facet_wrap( ~ PlantGeno, scales="free") +
  ylim(10,25) +
  ylab("Plant height (cm)") +
  xlab("") +
  theme_bw(base_size = 15)+
  theme(axis.text.x=element_blank()) +
  scale_fill_manual(values = c("#999999", "#E69F00", "#56B4E9"), name = "Lignage", labels = c("Mock", "Initial PVY population", "Final PVY population"))
```

```
# ggsave("./FigureS1C_height.svg")
```

##### Statistics

A **Kruskal-Wallis** test has been used to compare the
mean weight of the mock-inoculate plants, the plants inoculated with the
initial PVY population and the plants inoculated with final PVY
populations in HD2173. If the Krukal-Wallis test was significant, a
**Nemenyi’s** non-parametric all-pairs comparison test has
been performed.

**HD173\_119N**

```
Data_virulence_HD2173_119N <- filter(Data_Virulence_B, PlantGeno=="HD2173_119N")

kruskal.test(Weight ~ Time, data=Data_virulence_HD2173_119N)
```

```
## 
##  Kruskal-Wallis rank sum test
## 
## data:  Weight by Time
## Kruskal-Wallis chi-squared = 1.7496, df = 2, p-value = 0.4169
```

**HD173\_101G**

```
Data_virulence_HD2173_101G <- filter(Data_Virulence_B, PlantGeno=="HD2173_101G")

kruskal.test(Height ~ Time, data=Data_virulence_HD2173_101G)
```

```
## 
##  Kruskal-Wallis rank sum test
## 
## data:  Height by Time
## Kruskal-Wallis chi-squared = 3.8026, df = 2, p-value = 0.1494
```

**HD173\_115K**

```
Data_virulence_HD2173_115K <- filter(Data_Virulence_B, PlantGeno=="HD2173_115K")

kruskal.test(Height ~ Time, data=Data_virulence_HD2173_115K)
```

```
## 
##  Kruskal-Wallis rank sum test
## 
## data:  Height by Time
## Kruskal-Wallis chi-squared = 4.703, df = 2, p-value = 0.09523
```

#### **Figure S1C: HD2173 and the 3 PVY variants (Plant weight)**

##### Figure S1c (Plant weight)

```
Data_Virulence_B$Time = factor(Data_Virulence_B$Time, levels=c("Mock", "Initial","Final"))
Data_Virulence_B$PlantGeno = factor(Data_Virulence_B$PlantGeno, levels=c("HD2173_119N", "HD2173_101G", "HD2173_115K"))

ggplot(data = Data_Virulence_B, aes(x=Time, y=Weight)) + 
  geom_boxplot(aes(fill=Time)) +
  facet_wrap( ~ PlantGeno, scales="free") +
  ylim(0,20) +
  ylab("Plant weight (g)") +
  xlab("") +
  theme_bw(base_size = 15)+
  theme(axis.text.x=element_blank()) +
  scale_fill_manual(values = c("#999999", "#E69F00", "#56B4E9"), name = "Lignage", labels = c("Mock", "Initial PVY population", "Final PVY population"))
```

```
# ggsave("./FigureS1C_weight.svg")
```

##### Statistics

A **Kruskal-Wallis** test has been used to compare the
mean weight of the mock-inoculate plants, the plants inoculated with the
initial PVY population and the plants inoculated with final PVY
populations in HD2173. If the Krukal-Wallis test was significant, a
**Nemenyi’s** non-parametric all-pairs comparison test has
been performed.

**HD173\_119N**

```
Data_virulence_HD2173_119N <- filter(Data_Virulence_B, PlantGeno=="HD2173_119N")

kruskal.test(Weight ~ Time, data=Data_virulence_HD2173_119N)
```

```
## 
##  Kruskal-Wallis rank sum test
## 
## data:  Weight by Time
## Kruskal-Wallis chi-squared = 1.7496, df = 2, p-value = 0.4169
```

**HD173\_101G**

```
Data_virulence_HD2173_101G <- filter(Data_Virulence_B, PlantGeno=="HD2173_101G")

kruskal.test(Weight ~ Time, data=Data_virulence_HD2173_101G)
```

```
## 
##  Kruskal-Wallis rank sum test
## 
## data:  Weight by Time
## Kruskal-Wallis chi-squared = 6.8816, df = 2, p-value = 0.03204
```

```
kwAllPairsNemenyiTest(Weight ~ Time, data=Data_virulence_HD2173_101G)
```

```
##         Mock  Initial
## Initial 0.947 -      
## Final   0.212 0.078
```

**HD173\_115K**

```
Data_virulence_HD2173_115K <- filter(Data_Virulence_B, PlantGeno=="HD2173_115K")

kruskal.test(Weight ~ Time, data=Data_virulence_HD2173_115K)
```

```
## 
##  Kruskal-Wallis rank sum test
## 
## data:  Weight by Time
## Kruskal-Wallis chi-squared = 6.7104, df = 2, p-value = 0.0349
```

```
kwAllPairsNemenyiTest(Weight ~ Time, data=Data_virulence_HD2173_115K)
```

```
##         Mock  Initial
## Initial 0.323 -      
## Final   0.029 0.782
```

### **Figure S2:** Virulence of the eight final PVY populations inoculated serially onto the pepper lines HD219 and HD2344

#### **HD219**

##### Figure

```
Data_virulence_HD219 <- filter(Data_Virulence_A, PlantGeno=="HD219")
Data_virulence_HD219_WithoutMock <- Data_virulence_HD219[Data_virulence_HD219$ViralPop != "Mock", ] 
Data_virulence_HD219_WithoutMock$ViralPop = factor(Data_virulence_HD219_WithoutMock$ViralPop, levels=c("Initial","L9","L10","L11","L12","L13","L14","L15","L16"))


# Plant height
#-----------------------------------------

ggplot(data = Data_virulence_HD219_WithoutMock, aes(x=ViralPop, y=Height)) + 
  geom_boxplot(aes(fill=ViralPop)) +
  facet_wrap( ~ PlantGeno, scales="free") +
  ylim(0,30) +
  ylab("Plant height (cm)") +
  xlab("") +
  theme_bw(base_size = 15)+
  theme(axis.text.x=element_blank()) +
  scale_fill_manual(values = c("#E69F00", rep("#56B4E9",8)))
```

```
# ggsave("./FigureS2_219_height.svg")

# Plant weight
#-----------------------------------------

ggplot(data = Data_virulence_HD219_WithoutMock, aes(x=ViralPop, y=Weight)) + 
  geom_boxplot(aes(fill=ViralPop)) +
  facet_wrap( ~ PlantGeno, scales="free") +
  ylim(0,25) +
  ylab("Plant weight (g)") +
  xlab("") +
  theme_bw(base_size = 15)+
  theme(axis.text.x=element_blank()) +
  scale_fill_manual(values = c("#E69F00", rep("#56B4E9",8)))
```

```
# ggsave("./FigureS2_219_weight.svg")
```

##### Statistics

```
# Plant height
#-----------------------------------------

dunnettTest(x=Data_virulence_HD219_WithoutMock$Height, g=Data_virulence_HD219_WithoutMock$ViralPop)
```

```
##     Initial
## L9  0.177  
## L10 0.802  
## L11 1.000  
## L12 0.475  
## L13 0.325  
## L14 0.098  
## L15 1.000  
## L16 1.000
```

```
# Plant weight
#-----------------------------------------

dunnettTest(x=Data_virulence_HD219_WithoutMock$Weight, g=Data_virulence_HD219_WithoutMock$ViralPop)
```

```
##     Initial
## L9  0.0046 
## L10 0.0673 
## L11 0.6346 
## L12 0.1682 
## L13 0.1373 
## L14 0.1014 
## L15 0.3039 
## L16 0.8353
```

#### **HD2344**

##### Figure

```
Data_virulence_HD2344 <- filter(Data_Virulence_A, PlantGeno=="HD2344")
Data_virulence_HD2344_WithoutMock <- Data_virulence_HD2344[Data_virulence_HD2344$ViralPop != "Mock", ] 
Data_virulence_HD2344_WithoutMock$ViralPop = factor(Data_virulence_HD2344_WithoutMock$ViralPop, levels=c("Initial","L33","L34","L35","L36","L37","L38","L39","L40"))


# Plant height
#-----------------------------------------

ggplot(data = Data_virulence_HD2344_WithoutMock, aes(x=ViralPop, y=Height)) + 
  geom_boxplot(aes(fill=ViralPop)) +
  facet_wrap( ~ PlantGeno, scales="free") +
  ylim(0,30) +
  ylab("Plant height (cm)") +
  xlab("") +
  theme_bw(base_size = 15)+
  theme(axis.text.x=element_blank()) +
  scale_fill_manual(values = c("#E69F00", rep("#56B4E9",8)))
```

```
# ggsave("./FigureS2_2344_height.svg")

# Plant weight
#-----------------------------------------

ggplot(data = Data_virulence_HD2344_WithoutMock, aes(x=ViralPop, y=Weight)) + 
  geom_boxplot(aes(fill=ViralPop)) +
  facet_wrap( ~ PlantGeno, scales="free") +
  ylim(0,25) +
  ylab("Plant weight (g)") +
  xlab("") +
  theme_bw(base_size = 15)+
  theme(axis.text.x=element_blank()) +
  scale_fill_manual(values = c("#E69F00", rep("#56B4E9",8)))
```

```
# ggsave("./FigureS2_2344_weight.svg")
```

##### Statistics

```
# Plant height
#-----------------------------------------

dunnettTest(x=Data_virulence_HD2344_WithoutMock$Height, g=Data_virulence_HD2344_WithoutMock$ViralPop)
```

```
##     Initial
## L33 1.00   
## L34 1.00   
## L35 1.00   
## L36 0.53   
## L37 1.00   
## L38 9e-05  
## L39 1.00   
## L40 1.00
```

```
# Plant weight
#-----------------------------------------

dunnettTest(x=Data_virulence_HD2344_WithoutMock$Weight, g=Data_virulence_HD2344_WithoutMock$ViralPop)
```

```
##     Initial
## L33 1.00   
## L34 1.00   
## L35 1.00   
## L36 0.45   
## L37 1.00   
## L38 1.8e-06
## L39 0.43   
## L40 0.61
```

### **Table S2:** Competition experiments between PVY variants in pepper DH lines

The p-values may vary slightly from that published due to Monte Carlo
simulations.

#### **SON41-119N vs. SON41-115K-119N**

**Bonferroni correction** for multiple testing:

- p = 0.05 –> 0.008333333
- p = 0.01 –> 0.001666666
- p = 0.001 –> 0.0001666666

**HD2173**

```
chisq.test(c(10,1), p=c(0.33, 0.67), simulate.p.value = T, B = 2000000)
```

```
## 
##  Chi-squared test for given probabilities with simulated p-value (based
##  on 2e+06 replicates)
## 
## data:  c(10, 1)
## X-squared = 16.684, df = NA, p-value = 0.000111
```

**HD219**

```
chisq.test(c(19,1), p=c(0.33, 0.67), simulate.p.value = T, B = 2000000)
```

```
## 
##  Chi-squared test for given probabilities with simulated p-value (based
##  on 2e+06 replicates)
## 
## data:  c(19, 1)
## X-squared = 34.772, df = NA, p-value = 5e-07
```

**HD2256**

```
chisq.test(c(8,2), p=c(0.33, 0.67), simulate.p.value = T, B = 2000000)
```

```
## 
##  Chi-squared test for given probabilities with simulated p-value (based
##  on 2e+06 replicates)
## 
## data:  c(8, 2)
## X-squared = 9.991, df = NA, p-value = 0.003209
```

**HD2321**

```
chisq.test(c(21,0), p=c(0.33, 0.67), simulate.p.value = T, B = 2000000)
```

```
## 
##  Chi-squared test for given probabilities with simulated p-value (based
##  on 2e+06 replicates)
## 
## data:  c(21, 0)
## X-squared = 42.636, df = NA, p-value = 5e-07
```

**HD2344**

```
chisq.test(c(19,1), p=c(0.33, 0.67), simulate.p.value = T, B = 2000000)
```

```
## 
##  Chi-squared test for given probabilities with simulated p-value (based
##  on 2e+06 replicates)
## 
## data:  c(19, 1)
## X-squared = 34.772, df = NA, p-value = 5e-07
```

**HD2349**

```
chisq.test(c(19,0), p=c(0.33, 0.67), simulate.p.value = T, B = 2000000)
```

```
## 
##  Chi-squared test for given probabilities with simulated p-value (based
##  on 2e+06 replicates)
## 
## data:  c(19, 0)
## X-squared = 38.576, df = NA, p-value = 5e-07
```

#### **SON41-119N vs. SON41-115M-119N**

**Bonferroni correction** for multiple testing:

- p = 0.05 –> 0.008333333
- p = 0.01 –> 0.001666666
- p = 0.001 –> 0.0001666666

**HD2173**

```
chisq.test(c(17,0), p=c(0.33, 0.67), simulate.p.value = T, B = 2000000)
```

```
## 
##  Chi-squared test for given probabilities with simulated p-value (based
##  on 2e+06 replicates)
## 
## data:  c(17, 0)
## X-squared = 34.515, df = NA, p-value = 5e-07
```

**HD219**

```
chisq.test(c(20,7), p=c(0.33, 0.67), simulate.p.value = T, B = 2000000)
```

```
## 
##  Chi-squared test for given probabilities with simulated p-value (based
##  on 2e+06 replicates)
## 
## data:  c(20, 7)
## X-squared = 20.602, df = NA, p-value = 1.35e-05
```

**HD2256**

```
chisq.test(c(7,7), p=c(0.33, 0.67), simulate.p.value = T, B = 2000000)
```

```
## 
##  Chi-squared test for given probabilities with simulated p-value (based
##  on 2e+06 replicates)
## 
## data:  c(7, 7)
## X-squared = 1.8299, df = NA, p-value = 0.2533
```

**HD2321**

```
chisq.test(c(17,1), p=c(0.33, 0.67), simulate.p.value = T, B = 2000000)
```

```
## 
##  Chi-squared test for given probabilities with simulated p-value (based
##  on 2e+06 replicates)
## 
## data:  c(17, 1)
## X-squared = 30.736, df = NA, p-value = 1e-06
```

**HD2344**

```
chisq.test(c(25,1), p=c(0.33, 0.67), simulate.p.value = T, B = 2000000)
```

```
## 
##  Chi-squared test for given probabilities with simulated p-value (based
##  on 2e+06 replicates)
## 
## data:  c(25, 1)
## X-squared = 46.901, df = NA, p-value = 5e-07
```

**HD2349**

```
chisq.test(c(30,0), p=c(0.33, 0.67), simulate.p.value = T, B = 2000000)
```

```
## 
##  Chi-squared test for given probabilities with simulated p-value (based
##  on 2e+06 replicates)
## 
## data:  c(30, 0)
## X-squared = 60.909, df = NA, p-value = 5e-07
```

### **Table S3:** Comparison of virus variant distributions in the plants after one month of competition

**Bonferroni correction** for multiple testing:

- p = 0.05 –> 0.0023809523 (\*)
- p = 0.01 –> 0.0004761905 (\*\*)
- p = 0.001 –> 0.00004761905 (\*\*\*)

**HD2173 vs H0**

```
chisq.test(c(27,1), p=c(0.33, 0.67), simulate.p.value = T, B = 2000000)
```

```
## 
##  Chi-squared test for given probabilities with simulated p-value (based
##  on 2e+06 replicates)
## 
## data:  c(27, 1)
## X-squared = 50.949, df = NA, p-value = 5e-07
```

**HD219 vs H0**

```
chisq.test(c(39,8), p=c(0.33, 0.67), simulate.p.value = T, B = 2000000)
```

```
## 
##  Chi-squared test for given probabilities with simulated p-value (based
##  on 2e+06 replicates)
## 
## data:  c(39, 8)
## X-squared = 53.098, df = NA, p-value = 5e-07
```

**HD2256 vs H0**

```
chisq.test(c(15,9), p=c(0.33, 0.67), simulate.p.value = T, B = 2000000)
```

```
## 
##  Chi-squared test for given probabilities with simulated p-value (based
##  on 2e+06 replicates)
## 
## data:  c(15, 9)
## X-squared = 9.4464, df = NA, p-value = 0.002986
```

**HD2321 vs H0**

```
chisq.test(c(38,1), p=c(0.33, 0.67), simulate.p.value = T, B = 2000000)
```

```
## 
##  Chi-squared test for given probabilities with simulated p-value (based
##  on 2e+06 replicates)
## 
## data:  c(38, 1)
## X-squared = 73.237, df = NA, p-value = 5e-07
```

**HD2344 vs H0**

```
chisq.test(c(44,2), p=c(0.33, 0.67), simulate.p.value = T, B = 2000000)
```

```
## 
##  Chi-squared test for given probabilities with simulated p-value (based
##  on 2e+06 replicates)
## 
## data:  c(44, 2)
## X-squared = 81.666, df = NA, p-value = 5e-07
```

**HD2349 vs H0**

```
chisq.test(c(49,0), p=c(0.33, 0.67), simulate.p.value = T, B = 2000000)
```

```
## 
##  Chi-squared test for given probabilities with simulated p-value (based
##  on 2e+06 replicates)
## 
## data:  c(49, 0)
## X-squared = 99.485, df = NA, p-value = 5e-07
```

**HD2173 vs HD219**

```
HD2173_219.chi = matrix(c(27,1,39,8), nrow = 2)
colnames(HD2173_219.chi) <- c("HD2173","HD219")
rownames(HD2173_219.chi) <- c("Double mutant predominant","Simple mutant predominant")
HD2173_219.chi
```

```
##                           HD2173 HD219
## Double mutant predominant     27    39
## Simple mutant predominant      1     8
```

```
chisq.test(HD2173_219.chi, simulate.p.value = T, B = 2000000)
```

```
## 
##  Pearson's Chi-squared test with simulated p-value (based on 2e+06
##  replicates)
## 
## data:  HD2173_219.chi
## X-squared = 3.0058, df = NA, p-value = 0.1415
```

**HD2173 vs HD2256**

```
HD2173_2256.chi = matrix(c(27,1,15,9), nrow = 2)
colnames(HD2173_2256.chi) <- c("HD2173","HD2256")
rownames(HD2173_2256.chi) <- c("Double mutant predominant","Simple mutant predominant")
HD2173_2256.chi
```

```
##                           HD2173 HD2256
## Double mutant predominant     27     15
## Simple mutant predominant      1      9
```

```
chisq.test(HD2173_2256.chi, simulate.p.value = T, B = 2000000)
```

```
## 
##  Pearson's Chi-squared test with simulated p-value (based on 2e+06
##  replicates)
## 
## data:  HD2173_2256.chi
## X-squared = 9.5776, df = NA, p-value = 0.003238
```

**HD2173 vs HD2321**

```
HD2173_2321.chi = matrix(c(27,1,38,1), nrow = 2)
colnames(HD2173_2321.chi) <- c("HD2173","HD2321")
rownames(HD2173_2321.chi) <- c("Double mutant predominant","Simple mutant predominant")
HD2173_2321.chi
```

```
##                           HD2173 HD2321
## Double mutant predominant     27     38
## Simple mutant predominant      1      1
```

```
chisq.test(HD2173_2321.chi, simulate.p.value = T, B = 2000000)
```

```
## 
##  Pearson's Chi-squared test with simulated p-value (based on 2e+06
##  replicates)
## 
## data:  HD2173_2321.chi
## X-squared = 0.057108, df = NA, p-value = 1
```

**HD2173 vs HD2344**

```
HD2173_2344.chi = matrix(c(27,1,44,2), nrow = 2)
colnames(HD2173_2344.chi) <- c("HD2173","HD2344")
rownames(HD2173_2344.chi) <- c("Double mutant predominant","Simple mutant predominant")
HD2173_2344.chi
```

```
##                           HD2173 HD2344
## Double mutant predominant     27     44
## Simple mutant predominant      1      2
```

```
chisq.test(HD2173_2344.chi, simulate.p.value = T, B = 2000000)
```

```
## 
##  Pearson's Chi-squared test with simulated p-value (based on 2e+06
##  replicates)
## 
## data:  HD2173_2344.chi
## X-squared = 0.026973, df = NA, p-value = 1
```

**HD2173 vs HD2349**

```
HD2173_2349.chi = matrix(c(27,1,49,0), nrow = 2)
colnames(HD2173_2349.chi) <- c("HD2173","HD2349")
rownames(HD2173_2349.chi) <- c("Double mutant predominant","Simple mutant predominant")
HD2173_2349.chi
```

```
##                           HD2173 HD2349
## Double mutant predominant     27     49
## Simple mutant predominant      1      0
```

```
chisq.test(HD2173_2349.chi, simulate.p.value = T, B = 2000000)
```

```
## 
##  Pearson's Chi-squared test with simulated p-value (based on 2e+06
##  replicates)
## 
## data:  HD2173_2349.chi
## X-squared = 1.773, df = NA, p-value = 0.3638
```

**HD219 vs HD2256**

```
HD219_2256.chi = matrix(c(39,8,15,9), nrow = 2)
colnames(HD219_2256.chi) <- c("HD219","HD2256")
rownames(HD219_2256.chi) <- c("Double mutant predominant","Simple mutant predominant")
HD219_2256.chi
```

```
##                           HD219 HD2256
## Double mutant predominant    39     15
## Simple mutant predominant     8      9
```

```
chisq.test(HD219_2256.chi, simulate.p.value = T, B = 2000000)
```

```
## 
##  Pearson's Chi-squared test with simulated p-value (based on 2e+06
##  replicates)
## 
## data:  HD219_2256.chi
## X-squared = 3.6587, df = NA, p-value = 0.0782
```

**HD219 vs HD2321**

```
HD219_2321.chi = matrix(c(39,8,38,1), nrow = 2)
colnames(HD219_2321.chi) <- c("HD219","HD2321")
rownames(HD219_2321.chi) <- c("Double mutant predominant","Simple mutant predominant")
HD219_2321.chi
```

```
##                           HD219 HD2321
## Double mutant predominant    39     38
## Simple mutant predominant     8      1
```

```
chisq.test(HD219_2321.chi, simulate.p.value = T, B = 2000000)
```

```
## 
##  Pearson's Chi-squared test with simulated p-value (based on 2e+06
##  replicates)
## 
## data:  HD219_2321.chi
## X-squared = 4.7544, df = NA, p-value = 0.03638
```

**HD219 vs HD2344**

```
HD219_2344.chi = matrix(c(39,8,44,2), nrow = 2)
colnames(HD219_2344.chi) <- c("HD219","HD2344")
rownames(HD219_2344.chi) <- c("Double mutant predominant","Simple mutant predominant")
HD219_2344.chi
```

```
##                           HD219 HD2344
## Double mutant predominant    39     44
## Simple mutant predominant     8      2
```

```
chisq.test(HD219_2344.chi, simulate.p.value = T, B = 2000000)
```

```
## 
##  Pearson's Chi-squared test with simulated p-value (based on 2e+06
##  replicates)
## 
## data:  HD219_2344.chi
## X-squared = 3.8909, df = NA, p-value = 0.09039
```

**HD219 vs HD2349**

```
HD219_2349.chi = matrix(c(39,8,49,0), nrow = 2)
colnames(HD219_2349.chi) <- c("HD219","HD2349")
rownames(HD219_2349.chi) <- c("Double mutant predominant","Simple mutant predominant")
HD219_2349.chi
```

```
##                           HD219 HD2349
## Double mutant predominant    39     49
## Simple mutant predominant     8      0
```

```
chisq.test(HD219_2349.chi, simulate.p.value = T, B = 2000000)
```

```
## 
##  Pearson's Chi-squared test with simulated p-value (based on 2e+06
##  replicates)
## 
## data:  HD219_2349.chi
## X-squared = 9.0986, df = NA, p-value = 0.002386
```

**HD2256 vs HD2321**

```
HD2256_2321.chi = matrix(c(15,9,38,1), nrow = 2)
colnames(HD2256_2321.chi) <- c("HD2256","HD2321")
rownames(HD2256_2321.chi) <- c("Double mutant predominant","Simple mutant predominant")
HD2256_2321.chi
```

```
##                           HD2256 HD2321
## Double mutant predominant     15     38
## Simple mutant predominant      9      1
```

```
chisq.test(HD2256_2321.chi, simulate.p.value = T, B = 2000000)
```

```
## 
##  Pearson's Chi-squared test with simulated p-value (based on 2e+06
##  replicates)
## 
## data:  HD2256_2321.chi
## X-squared = 13.58, df = NA, p-value = 0.000392
```

**HD2256 vs HD2344**

```
HD2256_2344.chi = matrix(c(15,9,44,2), nrow = 2)
colnames(HD2256_2344.chi) <- c("HD2256","HD2344")
rownames(HD2256_2344.chi) <- c("Double mutant predominant","Simple mutant predominant")
HD2256_2344.chi
```

```
##                           HD2256 HD2344
## Double mutant predominant     15     44
## Simple mutant predominant      9      2
```

```
chisq.test(HD2256_2344.chi, simulate.p.value = T, B = 2000000)
```

```
## 
##  Pearson's Chi-squared test with simulated p-value (based on 2e+06
##  replicates)
## 
## data:  HD2256_2344.chi
## X-squared = 13.087, df = NA, p-value = 0.0007025
```

**HD2256 vs HD2349**

```
HD2256_2349.chi = matrix(c(15,9,49,0), nrow = 2)
colnames(HD2256_2349.chi) <- c("HD2256","HD2349")
rownames(HD2256_2349.chi) <- c("Double mutant predominant","Simple mutant predominant")
HD2256_2349.chi
```

```
##                           HD2256 HD2349
## Double mutant predominant     15     49
## Simple mutant predominant      9      0
```

```
chisq.test(HD2256_2349.chi, simulate.p.value = T, B = 2000000)
```

```
## 
##  Pearson's Chi-squared test with simulated p-value (based on 2e+06
##  replicates)
## 
## data:  HD2256_2349.chi
## X-squared = 20.959, df = NA, p-value = 1.65e-05
```

**HD2321 vs HD2344**

```
HD2321_2344.chi = matrix(c(38,1,44,2), nrow = 2)
colnames(HD2321_2344.chi) <- c("HD2321","HD2344")
rownames(HD2321_2344.chi) <- c("Double mutant predominant","Simple mutant predominant")
HD2321_2344.chi
```

```
##                           HD2321 HD2344
## Double mutant predominant     38     44
## Simple mutant predominant      1      2
```

```
chisq.test(HD2321_2344.chi, simulate.p.value = T, B = 2000000)
```

```
## 
##  Pearson's Chi-squared test with simulated p-value (based on 2e+06
##  replicates)
## 
## data:  HD2321_2344.chi
## X-squared = 0.19722, df = NA, p-value = 1
```

**HD2321 vs HD2349**

```
HD2321_2349.chi = matrix(c(38,1,49,0), nrow = 2)
colnames(HD2321_2349.chi) <- c("HD2321","HD2349")
rownames(HD2321_2349.chi) <- c("Double mutant predominant","Simple mutant predominant")
HD2321_2349.chi
```

```
##                           HD2321 HD2349
## Double mutant predominant     38     49
## Simple mutant predominant      1      0
```

```
chisq.test(HD2321_2349.chi, simulate.p.value = T, B = 2000000)
```

```
## 
##  Pearson's Chi-squared test with simulated p-value (based on 2e+06
##  replicates)
## 
## data:  HD2321_2349.chi
## X-squared = 1.2709, df = NA, p-value = 0.443
```

**HD2344 vs HD2349**

```
HD2344_2349.chi = matrix(c(44,2,49,0), nrow = 2)
colnames(HD2344_2349.chi) <- c("HD2344","HD2349")
rownames(HD2344_2349.chi) <- c("Double mutant predominant","Simple mutant predominant")
HD2344_2349.chi
```

```
##                           HD2344 HD2349
## Double mutant predominant     44     49
## Simple mutant predominant      2      0
```

```
chisq.test(HD2344_2349.chi, simulate.p.value = T, B = 2000000)
```

```
## 
##  Pearson's Chi-squared test with simulated p-value (based on 2e+06
##  replicates)
## 
## data:  HD2344_2349.chi
## X-squared = 2.1763, df = NA, p-value = 0.2318
```
