# Supplementary material for: Within-plant genetic drift to control virus adaptation to host resistance genes
Source: PLoS Pathog. 2024 Aug 5;20(8):e1012424. doi: 10.1371/journal.ppat.1012424 (PMC11326801; doi:10.1371/journal.ppat.1012424)
Supplement: Attachment — Submitted filename: Response Reviewers.docx [file ppat.1012424.s011.docx]

We would like to thank both reviewers for their constructive criticism, which helped us to improve the manuscript. Please find below our responses to reviews received on June 10, 2022 (PPATHOGENS-D-22-00753).

**Major Issues**

**Reviewer #1:**

**-Results. It was difficult for me to reconcile the data presented in figures 4 and 5. In figure 4, differences between initial and final viral fitness are observed only in plant genotypes in which viruses have low initial virulence (except for HD2256), and regardless they have low (HD2321) or high (HD2173) Ne. In addition, the three hosts in which the virus have higher initial fitness show similar values after passages; again regardless Ne is low (HD219) or high (HD2173). In my view, results in figure 4 may indicate that those isolates with “more room” for improving fitness are those that show bigger changes after passages. Even at low Ne, the likelihood of generating and randomly passing a variant with some more fitness than the original one is high. The paradigmatic case would be HD2321: with such extremely low fitness, it is likely that a variant with higher fitness arises. On the other hand, those viruses already quite optimized in their fitness remain more or less unchanged. Although an interpretation opposite to the one provided by the authors, this is an equally interesting result.**

**In contrast, the prediction of the multivariate model in figure 5 is exactly the opposite: high Ne and initial fitness results in higher probability of fitness gain. Can the authors please clarify this point and why they chose to use the results of Figure 5 for their conclusions and not those in Figure 4 that have a lower degree of data transformation?**

In Figure 4, only the replicative fitness variables are analysed. It is only the change in fitness between the beginning and end of the experiment that is statistically tested, not the effect of genetic drift and selection. Indeed, as reviewer 1 points out, we can see that in general in our experiment: "isolates with "more room" for improving fitness are those that show bigger changes after passages". Thus the majority of lineages that evolved on HD2349, HD2344 and HD2173 (inoculated with SON41-101G) showed adaptation, while they initially had low replicative fitness. We agree with this interpretation since we then show statistically with the GLM that the initial replicative fitness explains, in part, the changes in fitness observed.

However, we also see that this is not a universal rule, as it is not the case for the lineages evolved in two DH lines. In HD2256, none of the 8 lineages increased in fitness, while their initial fitness was low. Furthermore, in HD2321, where the initial fitness is extremely low, 8 lineages became extinct, and only 2 adapted. The initial fitness variable alone cannot therefore fully explain the observed evolutionary trajectories.

For this reason, we performed GLM to statistically test the effect of 3 variables (initial replicative fitness, selection and genetic drift) on the observed changes in fitness, and we showed the result on Figure 5. These analyses allowed us to demonstrate that both the level of genetic drift, the initial fitness and the interaction between drift and initial fitness have a significant effect on the observed changes in fitness. Thus, there is almost no adaptation in lines HD2256 and HD2321 because the initial fitness was low and the level of genetic drift was high (while in HD2349, HD2344 and HD2173 the level of genetic drift was low).

Reviewer 1 says that in figure 5 "high Ne and initial fitness results in higher probability of fitness gain", but this is not what figure 5 and our model show. Indeed, a high Ne (Ne = 500) and a low initial fitness (Wi) result in a higher probability of fitness gain (Figure 5A). Conversely, when Ne and the initial fitness are high, the probability of fitness gain is lower since the lineages have already reached their maximum adaptation level. Figures 4 and 5 are therefore perfectly consistent.

**I think that, as formulated here, the idea of using genetic drift to increase the durability of resistance is quite a long shot. First, according to the authors rationale, it requires a virus with initially low fitness in the host. However, the composition of the virus population in the location where the crop would be planted is a factor that can be hardly controlled by the human intervention. Second, the success of this strategy requires an specialist virus that is allowed to evolve only in the crop. This is not the case for most viruses, and there is ample evidence of the role of alternative hosts in generating virus population genetic diversity that allows virus emergence (of which resistance breaking is a particular case). Thus, I think that the authors should be more cautious with this part of the discussion.**

We do not argue that using genetic drift will provide a systematically durable resistance but instead we argue that pyramiding major resistance gene(s) with QTLs imposing a strong genetic drift within a plant would best reduce virus adaptation and would be preferable in terms of durability potential. We agree with the reviewer about being more nuanced in this part of the discussion. These points are now addressed at the end of the discussion.

The two objections formulated by the reviewer hold regardless of the resistance considered. Nevertheless, the use of resistant varieties has proved effective and relatively durable in many situations, although not systematically. We agree that the composition of the virus population in the field can hardly be controlled by human intervention. However, thanks to epidemiological surveillance and field trials, we can get an idea of the viral isolates present in a field and check if some of these isolates are already adapted to the plant resistance. Then, the most effective resistance gene(s) against these isolates can be deployed. It is also true that alternative hosts could allow virus emergence (again, it holds for every resistance, not only those exerting a strong genetic drift). But it is worth noting that fitness trade-off following local adaptation on one host are also common. Therefore, virus evolved on alternative hosts will not always show a high fitness level on cultivated ones.

**Reviewer #2:**

**1.	In the results section, values for the effective population size (n_e), selection (s) and initial fitness (w_I) are simply stated as a given, without any further comments about how these values were measured, and limitations or caveats. As a reader, I find this very unsatisfying: these estimates are essential to the narrative the authors are building, and these are parameters (especially s and w_i), for which there is not a standard way to measure them and the informed reader will want to know what is being compared. Furthermore, the limitations/caveats of these measurements and how this affects interpretation of the results are not discussed meaningfully. Finally, there are serious shortcomings with how these different measurements have been made (see points 2-4).**

We agree with Reviewer 2 that presenting more extensively the measures of *s* and *N_e_* and discussing the limitations of our measurements would improve the manuscript. We added a new sub-section in the "Results" section called “Relevance and accuracy of parameters used to explain the virus evolutionary trajectories”. We present how we estimate the variables and their limitations in more details.

**2.	Concerning n_e, the estimate was made based on experimental data for a pool of genotypes (i.e., carrying different beneficial mutations) from a previous study. But this means that the estimate of n_e is also made for the pool of genotypes, and hence differences in infectivity for the different genotypes in different hosts are not considered. This is a critical shortcoming, as the expectation is that it will be harder for low fitness genotypes to infect, and therefore n_e estimates for that genotype should be lower (as measured with other, more direct methods). The effects of the interactions between virus and host genotypes on n_e are therefore not considered, and since that it precisely what the study purports to be about, I see this is a major weakness.**

Two main types of empirical approaches exist to measure *N_e_*: direct and indirect methods. The direct estimation method follows directly the demographic changes occurring in the population, while the indirect estimation method uses the genetic changes occurring in the population over time to estimate *N_e_*. For our experiment, we used the indirect method, which requires the use of a mixture of viral variants in order to follow the evolution of the frequency of each variant over time. We used *N_e_* estimated from 7 to 10 dpi which corresponds to the onset of plant systemic infection by PVY. This measure will be called “*N_e_*_systemic”, and it cannot be assessed for each DH line – PVY variant combination.

To follow the recommendations of Reviewer 2, we performed additional experiments implementing a more direct method in order of estimate a *N_e_* value for each DH line – PVY variant combination. We estimated *N_e_* at the inoculation stage using SON41p variants carrying a green fluorescent protein (GFP) reporter gene and a single amino acid substitution in VPg (101G, 115K or 119N mutation). We measured the mean number of PVY primary infection foci visualized by GFP fluorescence 7 days after mechanical inoculation of 8 to 20 pepper cotyledons per DH line – PVY variant combination. This number represents the size of the bottleneck at the inoculation stage, and therefore the strength of genetic drift. This measure will be called “*N_e_*_inoc”.

The two measures of *N_e_* (systemic and inoc) show a positive and significant correlation (Pearson’s r=0.82, p-value = 2.477e-14). Due to multicollinearity, they could not be used together in the generalized linear models (GLM). To choose which variable to retain, we performed GLM using either (i) the probability of PVY replicative fitness gain or (ii) the PVY final replicative fitness as response variables; and Wi and either *N_e_*_systemic or *N_e_*_inoc as explanatory variables (as well as their interaction). Firstly, the GLM results were globally similar whether we used *N_e_*_systemic or *N_e_*_inoc (same significant explanatory variables). Secondly, we used McFadden pseudo R2 as a measure of the proportion of variation explained by each model. We obtained McFadden pseudo R2 of 0.33 and 0.44 using *N_e_*_systemic in the model, for the probability of PVY replicative fitness gain and the PVY final replicative fitness, respectively. We obtained McFadden pseudo R2 of 0.26 using *N_e_*_inoc in the model, for both variables to be explained. We therefore retained the *N_e_*_systemic variable in our models since the McFadden R2 was always higher.

**3.	Concerning s, the estimate is made with respect to a variant with a beneficial VPg mutation, responsible for overcoming resistance. But how do we know that these variants are representative for high fitness variants in the different DH lines? And how do we know what the potential contribution of other mutations (in VPg and the rest of the genome is)? The “fitness” data clearly illustrate this complication, as some lineages have significant decreases or increases in fitness without the detection of any mutations in VPg (Figure 3). This approach at least gives a rough but hopefully useful indication, but at the very least it needs to be presented much more carefully. Finally, although this is unfortunately common practice, there are much better ways to measure the frequency of polymorphisms than measuring height of a peak in a chromatogram (Lines 361-363). (See** [**https://pubmed.ncbi.nlm.nih.gov/19819885/**](https://pubmed.ncbi.nlm.nih.gov/19819885/) **and for a recent review** [**https://www.mdpi.com/2073-4425/12/2/283**](https://www.mdpi.com/2073-4425/12/2/283)**).**

Our data provides an initial answer to the question of whether our variants are representative of variants with high fitness. Indeed, the lineages showing the greatest fitness gains have fixed mutations 115K and 119N or mutations 101G and 115K (lineages 26, 28, 27, 38 and 52) (Figure 4). We can therefore conclude that these double mutants have higher fitness levels than other mutants within the 3 HD lines considered. These two double mutants (115K-119N and 101G-115K) are among the 5 variants that were used to estimate *s*.

This being said, in our experiment comprising a limited number of evolutionary lineages, we do not exclude the fact that selection may have had an effect on the PVY evolutionary trajectories. However, all our models showed that *N_e_* explains better these evolutionary trajectories and the *s* variable was not retained by model selection. It is possible that the *s* variable, estimated in the frame of an independent experiment with different conditions, does not evaluate properly the selection intensity exerted by the plant on the virus populations in the frame of the present experiment. If *s* would have been estimated with other PVY variants and/or different initial variant frequencies, more representative of our experiment, it might have explained better the data. We now emphasize this point further in the article (L206 - 223 and L 265 - 268).

We agree that some mutations elsewhere in the genome could contribute to the observed fitness changes. However, only one lineage shows a significant increase in fitness without the detection of a mutation in the VPg (L56). Otherwise, fitness increases are always associated with the presence of one or two fixed mutations in the VPg. Moreover, we validated by reverse genetics that the substitutions 101G, 115K, 115M and 119N (84% of the *de novo* mutations) were responsible for PVY fitness gains in the pepper lines (the other, minor, *de novo* mutations have not been tested). The major resistance gene *pvr2^3^* present in the DH lines imposes a strong selection pressure on PVY populations, and the adaptation of these populations via mutations in VPg is well known. Our results are therefore sufficient to explain the huge majority of the observed changes in fitness.

Finally, to measure virus competitiveness, we assessed virus frequencies using the peak size of a chromatogram. We agree that more accurate methods exist. Nevertheless, in our experiment, we observed clear-cut differences in viral accumulation. Indeed, the highest peak was on average 3.5 times higher than the smallest one. As a consequence, the corresponding variant represents 78% of the virus population, a very strong difference compared to the minor variant. Moreover, in 15/22 co-infection cases (68%), the highest peak was at least 2 times higher and the major variant represented therefore 66% of the population. This shows that the method we used was sufficient, given the strong effects we measured.

**4.	Concerning w_i, it cannot be stressed enough that virus accumulation is not fitness. There are many reports (also for plant viruses) that make this case very clearly. In this setup (passaging in multiple hosts per evolutionary line) accumulation probably is a fitness component, but without further evidence it is not credible to simply treat it as fitness. Moreover, how does one know what fitness can be achieved in the different DH lines, to scale the distance from the optimum?**

We added sentences explaining that viral load is just one component of fitness, which we call "replicative fitness" as proposed in other articles (L 144 - 146). We now use the expression “replicative fitness” in the rest of the article.

We don't know the maximum fitness level that a viral population can reach in each DH line and we cannot exclude that the optimum replicative fitness was not reached at the end of the experimental evolution. That’s why we compared the final viral load with that of the starting clone in the same DH line. With this method, we are able to measure significant increases in viral load, and therefore in replicative fitness, which allows us to conclude about the adaptation of the viral population in this DH line.

**5.	These problematic measurements of key parameters are simply presented as fact, in effect not recognizing the difference between our models and reality. I would encourage the authors to give a much more modest presentation of their results, in which they acknowledge the shortcomings/caveats of their methods. In a way, this would not detract from the results, but make it much clearer to the reader that – surprisingly – from quite limited data that do require making some key assumptions, the authors can account for many of the observed patterns.**

The new sub-section in the "Results" section called “Relevance and accuracy of parameters used to explain the virus evolutionary trajectories” present our parameters and their limits.

The selection coefficient *s* was measured in each DH line following their inoculation with an equimolar mixture of 5 PVY variants (SON41-101G, -115K, -119N, -101G-115K and -115K-119N). The selection coefficient *s* was calculated as the difference between the growth rates of the fittest and the weakest variants in each DH line. Taken together, the 3 mutations 101G, 115K and 119N represent 48% (13/27) of the *de novo* mutations observed in the experimental evolution, and the 5 single and double mutations 101G, 115K, 119N, 101G-115K and 115K-119N represent 76% (42/55) of the mutations fixed in the final lineages. Therefore, similar variants were present in both experiments. However, both experimental contexts were not identical, the equimolar mixture being more complex (5 variants) than what we observed in our experimental evolution (maximum 2 VPg variants in a plant). Moreover, several mutations were present during the experimental evolution (especially the -115M) but absent in the experiment used to assess the parameter *s*. Therefore, the fact that *s* does not explain the observed evolutionary trajectories does not mean that selection has no effect, but rather that the estimation of *s* used is probably not accurate enough.

Regarding *N_e_*, we used *N_e_* estimated from 7 to 10 dpi which corresponds to the onset of plant systemic infection by PVY because it was never associated to multicollinearity issues between the explanatory variables. On the contrary, Ne estimated from 1 to 6 dpi, 11 to 14 dpi and 15 to 34 dpi showed multicollinearity issues with other explanatory variables and should be excluded from the GLMs. Even if this remains to be proven, we can hypothesize that the stochastic nature of *N_e_* makes it less sensitive than *s* to the composition of the viral population. This might explain why *N_e_* explains our data better than *s*.

**6.	The shortcomings for the different measurements raise another important issue: do we expect that n_e and s are two parameters that can be disentangled over hosts in practice? As the host becomes more resistant and it becomes more difficult for the pathogen to infect and spread, bottlenecks are likely to become more narrow. The authors appear to share this expectation (lines 262-265). Framing the study around the expectation that these two parameters can be disassociated, further adds unnecessary complexity and likely will lead to confusion with many readers (it sure did for me). Moreover, given the problems raised with the different parameter estimates, I wonder whether in reality these two parameters may be tightly associated.**

The reviewer wonders if *s* and *N_e_* can be dissociated in the studied hosts, and also if *N_e_* and the host resistance level (opposite to replicative viral load) can be dissociated. Our results show that these 3 parameters can be completely uncorrelated, at least for the 6 pepper DH lines used in our experiment (Figures 1 and 3). *Ne* and host resistance (or *Ne* and *s*) can be uncorrelated for example if narrow bottlenecks at inoculation or at the onset of systemic infection are followed by a great regrowth of the virus population at the systemic level, like during a foundation effect.

It is indeed challenging to unravel the effects of selection and genetic drift separately, and a growing number of models have been developed to answer this issue. Rousseau et al., (2017) estimated both parameters using a multi-allelic Wright-Fisher model developed for our pathosystem. They showed that it was possible to estimate both parameters independently in 15 doubled-haploid pepper lines (this result being validated by numerical simulations), and that both parameters were not correlated. Indeed, the 6 pepper lines that we selected show different selection and genetic drift regimes.

**Minor Issues**

**Reviewer #1:**

**-Experimental design. Perhaps this is more clearly explained in Rousseau et al 2017, but I think I would be helpful for the reader if the authors add a brief explanation on why some isolates are passaged in various hosts and others just in one. I initially thought that 101G and 115K were not infective in most pepper genotypes, but according to the M&M this is not the case.**

We agree with Reviewer 1, the explanation has been added at the beginning of the “Results and discussion” section.

**- Variables to measure. It is intriguing for me how despite big changes in virus multiplication effects in plant weight and height are very small, even in some cases the evolved viruses show lower virulence than the initial one basically having no effect on growth as compared to the mock plants. According to this, virus fitness gain would come at no cost for the plant. Then, is virus evolution a risk for the plant? How this relates to the effect of resistance breaking strains in field conditions? Is it possible that the effects of fitness gain come at the cost of reduction in other plant traits such as fruit production?**

In this experiment, we measured only two traits related to virulence: fresh weight and height of one-month-old plants. As Reviewer 1 points out, it is likely that the increase in viral accumulation has a cost for other plant traits that we did not measure, and/or that this cost will appear later in the plant development. We also know that the pepper DH lines show different levels of tolerance to the virus, so it is also possible that these costs are lower or absent in the more tolerant DH lines.

We added a sentence about it in the discussion.

**-Statistics. The authors use generalized linear models, could they provide information on the distribution fitted to the data and the method utilized to select the best-fitted distribution?**

We assumed a binomial distribution for the probability of fitness gain and a Gaussian distribution for the final fitness W_f_. This sentence has been added in the “Materials and Methods” section. The best-fitted distributions were selected using the Hosmer and Lemeshow goodness of fit test and the Chi-squared goodness of fit test for the probability of fitness gain and the final fitness W_f_, respectively.

**Reviewer #2**:

**- No motivation or even any explanation is given for why different ancestral virus genotypes were used. I’m sure the reader can piece it together from previous work, but why don’t they just brienfly explain this?**

It is now explained at the beginning of the “Results and discussion” section (L 111 – 120).

**- The parameter n_e is dose dependent and therefore subject to changes during the experiment (especially in a setup where the number of infected plants can vary), and likely to change as virus variants become more infectious and/or reach higher accumulation. Ideally one would account for these caveats, but as a bare minimum they should be mentioned.**

We added in the « Relevance and accuracy of parameters used to explain the virus evolutionary trajectories » sub-section that both *s* and *N_e_* can indeed vary according to the change of the composition of the virus populations. We make the hypothesis that *N_e_* may be less impacted by these changes due to its stochastic nature, even if it remains to be proven.

**- I am missing some context in the discussion. First, there is plenty of literature showing that small population sizes can be beneficial for populations evolving on rugged fitness landscapes (e.g.,** [**https://www.pnas.org/doi/10.1073/pnas.1410631112**](https://www.pnas.org/doi/10.1073/pnas.1410631112) **and many others), because they can lead to escape from local fitness optima.**

In our experiment, we have shown that strong genetic drift is favourable to the durability of resistance. Obviously, in other situations (such as the escape from local fitness optima), this might not be the case. We simply interpret our data, and demonstrate that despite few explanatory variables, genetic drift clearly has a positive effect on resistance durability in our case.

We now mention in the discussion that in other situations, genetic drift could also have beneficial effects for the virus population (L 324 - 328).

**- Second, there are interesting studies that consider the effect of mutational supply on plant virus evolution, for example what conditions favor the breaking of resistance against microRNAs (e.g., https://journals.asm.org/doi/10.1128/JVI.05326-11). Seems like a relevant example, since the different plant lineages help control mutational supply and strength of selection (although in this case strongly favoring the impact of mutational supply IIRC).**

We agree that this study in which within-plant viral census population sizes are controlled through the use of transgenic plants expressing different level of microRNAs is a relevant example, the frequency of adaptive mutations being correlated to the viral census population size. This study is now cited as an example (L 302 - 308).

**- Lines 14-15: As an evolutionary biologist, I hope we can one day claim the first line of the abstract, but that is a grand statement to make in the present.**

We use the conditional tense (“could avoid”) instead.

**- Lines 25-27: This line can easily be misconstrued to mean that most mutations occurred in VPg, so I would simply state that only VPg was analyzed.**

Done.

**- Lines 29-32: Rewrite, I find this summary here very confusing. “Fitness […] remained high” – how can it remain high if it was initially low?**

The sentence has been rewritten.

**- Line 68: “viral variants frequencies”**

Done.

**- Line 123: “that WENT extinct”**

Done.

**- Line 166: “[…] significant effect on the number of VPg mutations that occurred […]”**

Done.
