# Supplementary material for: Within-plant genetic drift to control virus adaptation to host resistance genes
Source: PLoS Pathog. 2024 Aug 5;20(8):e1012424. doi: 10.1371/journal.ppat.1012424 (PMC11326801; doi:10.1371/journal.ppat.1012424)
Supplement: Attachment — Submitted filename: Response Reviewers.docx [file ppat.1012424.s012.docx]

We sincerely appreciate the valuable feedback provided by the reviewers, which has greatly contributed to enhancing the quality of our manuscript. Below, we have detailed our responses.

**Part I – Summary**

**Please use this section to discuss strengths/weaknesses of study, novelty/significance, general execution and scholarship.**

**Reviewer #1:**

**The authors performed experimental evolution using Potato virus Y (PVY) on different pepper lines. The main aim of the study was to investigate whether virus adaptation could be controlled by favorably breeding certain plant genotypes over others. More specifically the authors asked the question whether “manipulation” of selection and genetic drift could help control virus adaptation and prevent a resistance breakdown. The question asked and the results presented are relevant to the field. However, the study also has some major weaknesses, as already pointed out by previous Reviewers #1 and #2. Most of these weaknesses could/were not addressed experimentally, but the authors have provided additional explanations to their results and acknowledged the shortcomings of their methods in the manuscript. These changes were crucial for the reader to better understand the study. Nevertheless, I also agree that the implementation of manipulating selection and genetic drift in the wild is quite a long shot.**

**Reviewer #2:**

**I have now read the manuscript “Within-plant genetic drift to control virus adaptation”. In this revised version, the authors have integrated a large part of the comments made by the reviewers of the first round of revisions and the quality and clarity of the manuscript has improved substantially. The question tackled in interesting and timely. The experiments are well conducted.**

**Reviewer #3:**

**Plant viruses pose a significant threat to crop yields. To mitigate these losses, there have been efforts to introduce genes that confer resistance to these viruses. However, these viruses can evolve to overcome such resistance. The authors have employed experimental evolution in an exceptionally well-designed experiment to assess how host-imposed conditions affect virus evolution. They discovered that host genotypes enforcing low effective population size (Ne) and reduced replicative fitness in the virus hinder the virus's ability to adapt to the host, evidenced by numerous virus lineages going extinct under these conditions.**

**While the authors primarily frame their findings within an agricultural context, the implications extend beyond this scope. The insights are also valuable for understanding virus evolution more broadly.**

**The manuscript has undergone prior review before my evaluation. The preceding reviewers have conducted a thorough examination of this manuscript and have raised pertinent comments. Based on the authors' responses and the revised manuscript, it appears that the authors have satisfactorily addressed these comments; the manuscript now includes more detailed and/or clarified text in response to reviewers' suggestions, enhancing both the manuscript's comprehensibility and the precision of its discussions.**

**Part II – Major Issues: Key Experiments Required for Acceptance**

*Please use this section to detail the key new experiments or modifications of existing experiments that should be absolutely required to validate study conclusions.*

*Generally, there should be no more than 3 such required experiments or major modifications for a "Major Revision" recommendation. If more than 3 experiments are necessary to validate the study conclusions, then you are encouraged to recommend "Reject".*

**Reviewer #1:**

**1. The authors promote that the best solution to an increased resistance durability is to use plant genotypes/lines on which the virus has a low effective population size (Ne) and a low initial viral replicative fitness (Wi). However, in nature it is unlikely that only one virus variant circulates, and therefore it is most likely hard to find a suitable plant genotype for all viral variants. Indeed their results demonstrate that the outcome can be highly variable depending on the pepper line and virus variant used, where a high Ne and a high Wi appears to also prevent virus adaptation (pepper HD2173 and virus SON41-115K). The same accounts for the combination of a high Ne and intermediate Wi (pepper HD2173 and virus SON41 119N).**

The challenges posed by diverse viral variants are inherent to any resistance strategy, as there will always be the possibility of a viral variant emerging that can potentially overcome a given resistance. However, for resistance to be as durable as possible, our results show that, for our pathosystem, it is preferable to combine resistance (low Wi) with high genetic drift (low Ne). It could be done by pyramiding resistance gene(s) and QTL(s) controlling genetic drift. We do not promote our solution as “absolute” (i.e. always avoiding PVY adaptation whatever the situation or the epidemiological context) but less risky compared to other plant genotype choices. We are also aware that the plant genotype choice is not the sole lever against pathogen adaptation and should be complemented with other levers in adequate strategies (but this is not the scope of our study).

The pepper-PVY variant combinations cited by reviewer 1 do not prevent getting adapted PVY variants. This is because there was no adaptation during the experiment since the virus was already well adapted (Wi is relatively high for both examples). Consequently, these pepper genotypes cannot be considered resistant and are therefore not suitable for field deployment. On the contrary, the only pepper genotypes capable of preventing adaptation in our study are those combining low Wi (indicating resistance) with high genetic drift.

**2. In a previous study the authors had already estimated the selection coefficient (s) and the effective population size (Ne) of PVY on the pepper lines used in this study. As also pointed out by previous Reviewer #2, these estimates were done using a more complex mixture of PVY variants (5 to be precise). Whereas in this study 3 of these variants have been evolved in isolation in different pepper lines. This can have a large effect on the s and Ne estimates. In their answer to previous Reviewer #2, the authors explain they have performed additional experiments to measure Ne with a more direct method (Ne_inoc instead of Ne_systemic). Even though this method may also not be an ideal way to measure Ne (the presence of GFP might also have an effect on viral replication success), I think it is a valuable addition to the manuscript. Therefore, it is not clear to me why the authors decided not to include the results of this additional measurement in the manuscript/supplementary material.**

We have now added this additional measure of Ne to the article. We discuss it in the choice of explanatory variables as well as in the Materials and Methods section. The data are also available in the supplementary material (S2 Dataset, S1 and S2 files, which respectively contain the table with Ne estimates and the R Markdown script with all the statistical analyses).

We had initially chosen not to include the Ne_inoc variable in the paper because it is strongly correlated with other Ne estimates, and it was not retained in the GLM (see our response to reviewer 2).

**3. There is only information about Ne for the first passage. Ne_systemic and Ne_inoc were both estimated from 7 to 10 days post infection. There is no information on Ne in the following passages. As only a very small sample of the plant was taken for the following passages, I assume that the bottleneck size was quite strong, selecting only for those variants that are present at a high frequency in the virus population. It is important to consider this effect as well.**

We cannot estimate Ne for the following passages as it is technically not possible. Indeed, with the method used by Rousseau *et al.,* it would be necessary to develop new genetic markers for each population at each cycle, because viral populations evolve constantly, which is not realistic. With the GFP-tagged variant method, it would be necessary to tag all populations and their variants with GFP, which is also impossible.

Nevertheless, we are convinced that there are quite narrow bottlenecks occurring. At the sampling stage, we take 1g of leaf, which is quite substantial, so in our opinion, this does not cause significant bottlenecks. However, during inoculation and systemic infection, there are certainly strong bottlenecks. We believe that our Ne estimates are quite robust to the composition of the viral population, and therefore can account for these bottlenecks as well.

**Reviewer #2:**

**The point that remains problematic is the use of the variables Ne and s, derived from experimental data in a previous study by the same group and published in PLoS Pathogens. Regarding Ne, I find problematic that the authors use the Ne evaluated from 7 to 10 dpi to explain evolutionary dynamics occurring in an experimental set up where viruses are passaged every 28 days. It means that they account for only part of the story. In particular, the infection bottleneck is likely to have a quite important role and it is not accounted for. This should at least be discussed and ideally a more time-integrative measure of Ne should be used.**

As we indicated to reviewer 1, all Ne estimates are now available (S2 Dataset). We also try to include a more time-integrative measure of Ne by calculating the harmonic mean of all Ne estimates at each date post inoculation (spanning from 0 to 34 dpi). Regarding the choice of the Ne estimate, we are now justifying it in the manuscript and have added the analysis scripts.

Ne estimates are generally highly correlated (this includes Ne_inoc, Ne_1_6, Ne_7_10, Ne_11_14, Ne_15_34 and the harmonic mean of the Ne estimates at different dates post inoculation). We conducted all possible GLMs with three explanatory variables (2 Ne estimates and Wi, or 3 Ne estimates). The 35 models incorporating all possible combinations of three explanatory variables displayed extremely high Variance Inflation Factors (VIFs) exceeding 100 (S1 and S2 Files). This is mainly because we only have 8 pepper-PVY variant combinations. Therefore, correlations between explanatory variables are based on 8 points, and collinearity issues quickly arise. We can therefore only retain two explanatory variables in the models. We conducted all possible GLMs with two explanatory variables (1 Ne estimate and Wi, or 2 Ne estimates). The Akaike Information Criterion (AIC) was used to select the best model. The models exhibiting the lowest AIC always included Wi and Ne estimated from 7 to 10 dpi, for both response variables. Moreover, models including these explanatory variables always have the highest McFadden's R-squared and R-squared (0.33 and 0.42). As a result, this Ne estimate was retained in our analyses. With our data, we can't precisely estimate the role of genetic drift at each stage of infection, but we can estimate an overall effect of genetic drift. Everything is presented in the section “Relevance and accuracy of parameters used to explain the virus evolutionary trajectories”, and also in the R Markdown script.

**Regarding s, I find it very difficult to understand what s represents. It seems to be the opportunity for selection among 5 viral variants in a plant genotype. First, I find the name “s” confusing as it is usually used for the selective coefficient characteristic of a variant (in competition with a reference strain) and not of a host or environment. Then it is measured as “the difference between the growth rates of the fittest and the weakest variants” and the identity of these two variants is not the same from one host genotype to another and also potentially different from the two variants in competition in each of the experimentally evolving lines (i.e. the variant used to start experimental evolution and the main variant arising by mutation in this lineage), such that the potential explanatory link between this “s” and the evolution of a resistance breakdown is not clear from the beginning. And this variable actually never has a significant effect in the models presented, which is actually not surprising. My suggestion is actually to remove totally this variable from the analysis, as both the introduction and the interpretation are focus of Ne and drift effects. It would make the whole story a lot clearer and more straightforward.**

We have followed your recommendation and have completely removed 's' from the manuscript.

**Reviewer #3:**

**There are two issues with the wording that need clarification or revision for the manuscript to be considered accepted (persuasive explanations may also suffice):**

**L236-237 "Combining a strong within-plant genetic drift and a low PVY initial replicative fitness can prevent virus evolution"**

**The authors note the emergence of new mutations and alterations in replicative fitness. They found this even without investigating mutations in other genes, shifts in the virus quasispecies, or different virus fitness phenotypes. Therefore, asserting that virus evolution is entirely prevented is misleading (is it possible to entirely halt evolution?). I strongly suggest rephrasing this to "Combining a strong within-plant genetic drift and a low PVY initial replicative fitness can prevent virus adaptation to host-resistance genes" or similar.**

We agree, the sentence has been modified.

**Similarly, I propose refining** **the title of the manuscript for greater specificity, perhaps to "Within-plant genetic drift to control virus adaptation** **to host-resistance genes", since the authors cannot confirm if the viruses are undergoing other forms of adaptation.**

The title of the manuscript has been modified.

**Part III – Minor Issues: Editorial and Data Presentation Modifications**

*Please use this section for editorial suggestions as well as relatively minor modifications of existing data that would enhance clarity.*

**Reviewer #1:**

**1. Something that is unclear: If the authors went through the effort of doing a 7 month long evolution experiment, why didn’t they use Illumina sequencing to sequence the entire genome of the ancestral and evolved virus populations? I am not asking the authors to do this. But with the low sequencing costs nowadays, especially of a small viral genome, this only makes sense to me. This would have given a more quantitative estimation of the variants present in the VPg cistron, show what happens in the rest of the genome, and possibly give explanations of fitness gains/losses that can not be explained with the current data.**

We agree that high-throughput sequencing could have allowed for a finer estimation of adaptation by identifying potential candidate mutations outside of VPg. However, sequencing only the VPg gene remains sufficient to meet the objectives of our study for two reasons.

Firstly, the primary aim of our study was to investigate the impact of the evolutionary forces imposed by the plant on viral populations. Therefore, our main objective was not to identify all determinants of viral adaptation, but rather to identify plant characteristics influencing the fate of the viral population.

Secondly, we know that VPg is a strong candidate gene for explaining adaptation, as we have already demonstrated that numerous viral mutations conferring adaptation to pvr23 are found in this gene. Sequencing of VPg was therefore sufficient to provide an initial insight into candidate mutations explaining PVY adaptation to the major pvr23 resistance gene. Indeed, among the 16 adapted viral populations, 15 have fixed mutations known to confer adaptation to pvr23 between positions 101 and 119 of the VPg. Only the adaptation of population L56 is not explained by mutations in the VPg. Additionally, 7 other viral populations have fixed mutations known to confer adaptation to pvr23 between positions 101 and 119 of the VPg, without showing a significant increase in fitness. However, for 6 of them, replicative fitness is at least two times higher than that of the initial viral population. The change is not significant due to a statistical power issue but would certainly be with testing more plants.

Finally, we agree that conducting high-throughput sequencing could have identified mutations explaining decreases in fitness, potentially responsible for the Muller's ratchet, which is one of our hypotheses to explain extinctions.

**2. Make all raw data/scripts available (Zenodo repository) so that the analyses can be reproduced.**

All input files, including the raw data, the R Markdown script covering all statistical analyses performed in this article, the generated figures and tables, and an HTML-formatted R Markdown report containing comprehensive statistical analyses, are now available in the supplementary material (S1, S2, S3 datasets and S1 and S2 files).

**3. Along the whole manuscript correct phrases like in line 214: “..that were fixed during the experimental evolution..” to “..that were fixed during the evolution experiment..”**

All the sentences have been corrected.

**4. Line 455: correct the figure number: “..experimental evolution results(Figs 2A, 2B ..)”**

It has been corrected.

**Reviewer #2:**

***Minor comments:***

**L311-315 + l333: Muller’s ratchet can indeed explain extinctions but here, the authors haven’t detected any mutations in the populations which got extinct, so the accumulation of deleterious mutations do not seem to be the reason why they got extinct. A purely demographic effect seems more likely.**

While we did not identify mutations in the extinct populations (except for L22, which acquired an adaptive mutation), it is worth noting that we only sequenced the VPg. Deleterious mutations may have accumulated elsewhere in the genome. Regarding demographic effects, extinctions occurred only during the third and fourth passages, not during the first ones. Therefore, it seems unlikely that only demographic effects are at play.

**In their response to one of the previous reviews the authors write “we argue that** **pyramiding major resistance gene(s) with QTLs imposing a strong genetic drift within a plant would best reduce virus adaptation and would be preferable in terms of durability potential”. The authors actually do not say this in the paper. The conclusion remains very vague on the strategies to reduce Ne and thus adaptation potential and would strongly benefit from adding this more “mechanistic” strategy.**

This has been added to the discussion.

**L177: no lack of adaptive mutations but can there be a difference in mutation supply (linked to the number of viral replications)?**

We assume that the number of mutations is correlated with Wi (the within-plant viral concentration). For HD219, Wi is high, so it is unlikely that the mutation supply explains the absence of adaptive mutations. However, for HD2256, Wi is low. The mutation supply could therefore also explain the absence of adaptive mutations, in addition to the effect of genetic drift. We have added this hypothesis to the article

***Typos and formulation:***

**L25: repetition of « contrasted ».**

The second “contrasted” has been replaced.

**L27: I guess the authors mean “linking” and not “inking”.**

Thank you, it has been corrected.

**L29: replace “evolutionary forces” by “drift and selection”**

It has been replaced by “genetic drift”, since we do not study the effect of selection anymore in this version of the article.

**L33: replace “and constant whatever” by “independently of”**

It has been replaced.

**L105: “divergent” is not the best word choice in this context as it suggests divergent evolution.**

**“different” might be better.**

It has been replaced by “Different”.

**L112: clarify “initial viral load”: is it the viral load at x dpi for the ancestral population or the viral load just after inoculation?**

It has been modified.

**L117: remove “and” at the end of the line**

It has been removed.

**L121: adaptation to what? To which plant genotype?**

Adaptation to the resistance allele. It has been added.

**L185-187: phrase mal foutue**

The sentence has been simplified.

**L259: final replicative fitness remains is high independently of the replicative fitness of the initially inoculated virus.**

It has been modified.

**L344: remove ‘viral”**

It has been removed.

**L345: “favouring” instead of “favoured”? Otherwise I do not get this sentence.**

The sentence has been simplified.

**L594: Rousseau et al is reference 11 and not 10**

It has been changed.

***Reviewer #3:***

**1) The are numerous studies showing how in different potyviruses the VPg is where more adaptive mutations are accumulated. I suggest to add some of these references to strength the decision of only sequencing the VPg gene. This could go after L126.**

We added several references showing that, in different potyviruses, the VPg is where adaptive mutations are likely to accumulate.

**Furthermore, the authors might consider citing instances of potyviruses adapting to hosts through mutations in other genomic regions and explicitly acknowledging that mutations in the non-sequenced portions of the genome could contribute to their findings. For instance, the statement in L177 about the “lack of adaptive mutations for PVY in these plant genotypes” should be more concise and refer only to l” “****lack of adaptive mutations for VPg protein of PVY TO these plant genotypes”.**

A sentence explaining that other mutations elsewhere in unsequenced regions of the genome could also impact PVY replicative fitness has been added with several references. The second sentence has been modified.

**2) L248 "Similar results were obtained after excluding the nine lineages that became extinct before the experiment concluded." The primary analysis was conducted without omitting the nine extinct lineages, which I believe is accurate, and reanalyzing without these lineages serves as a solid control. However, in Figure 4A, it's unclear (due to the absence of data points on the bars) whether the replicative fitness includes the extinct lineages. Considering these extinct lineages as having a fitness value of zero might provide a more balanced representation of replicative fitness. If only surviving lineages are displayed, it appears that viruses in HD2331 experience a significant fitness boost, despite most lineages evolving there having gone extinct.**

**Please specify whether the extinct lineages were factored into this figure and, if not, please annotate and discuss this in the manuscript.**

In Figure 4A, only lineages for which Wf could be measured were considered. Therefore, extinct lineages are not included in this figure. We have added this information for clarity in the caption. The objective of this figure and the associated statistics was to identified significant increases or decreases in replicative fitness among non-extinct lineages. For those that are extinct, it is evident that there has been a significant decrease in fitness, so they were not included.

**3) In responses to earlier reviews, the authors indicated that DH lines exhibit varying degrees of virus tolerance. This variation should be acknowledged in the manuscript, as the level of tolerance could influence adaptation to host-resistance genes significantly.**

**Note that host tolerance can significantly impact virus evolution
(**[**https://doi.org/10.1111/nph.17466**](https://doi.org/10.1111/nph.17466)**)**

We agree that tolerance could influence the evolution of viral populations. However, we did not quantify the level of tolerance for the DH lines used in the study and therefore do not have this data. We added in the discussion that the part of variance of PVY adaptation remaining unexplained could be explained in part by the tolerance of the DH lines:

“Although our GLMs explained much of the variation in PVY adaptation, additional explanatory variables could be estimated to further explain PVY adaptation. Among these variables, plant tolerance to virus infection could significantly influence virus adaptation to host resistance, as shown by Montes et al (25). The pepper DH lines used in the present study showed varying degrees of symptom, which could be related to different levels of tolerance. However, we did not carry out a thorough quantitative estimation of these tolerance differences, which is necessary to incorporate them into GLMs.”

**4) L27 I recommend to clarify also in the abstract that the study sequences the PVY VPg cistron because here is where most mutations responsible for PVY adaptation to pvr23 are likely to occur.**

Is has been added in the abstract.

**5) To enhance the interpretability of figures 1 and 4 (and potentially 3), consider using a unique color to denote pepper genotypes where viruses exhibit both low Ne and low fitness**

The two pepper genotypes with both low Ne and Wi are now highlighted in blue in all three figures.

**6) Some typographical errors:**

**L27 "inking" should presumably be "linking."**

**L594 Rousseau et al. (10), the citation should be 11, not 10.**

It has been changed.
